# Structural tuning of β-enamino diketones: exploration of solution and crystalline state photochromism

**DOI:** 10.3389/fchem.2023.1295347

**Published:** 2023-11-07

**Authors:** Kiran B. Manjappa, Sheng-Chieh Fan, Ding-Yah Yang

**Affiliations:** ^1^ Graduate Program for Biomedical and Materials Science, Tunghai University, Taichung, Taiwan; ^2^ Department of Chemistry, Tunghai University, Taichung, Taiwan

**Keywords:** β-enamino diketones, thiophene, photochromism, piezochromism, multi-responsive, photosensitive, microwave, functional property

## Abstract

A library of β-enamino diketones was prepared via base-mediated, three-component reaction of 4-hydroxycoumarins with various aromatic/aliphatic amines and β-nitrostyrenes under microwave irradiation conditions to investigate their photochemical properties. Among the prepared compounds, a thiophene derived β-enamino diketone was found to be light-sensitive and to exhibit unique photochromic behavior, that is, positive photochromism in solution and negative photochromism in crystalline phase. In addition, this prepared photochromic compound was further covalently linked to a structure-related, piezochromic β-enamino diketone moiety to explore its potential multi-stimuli responsive properties.

## Graphical Abstract

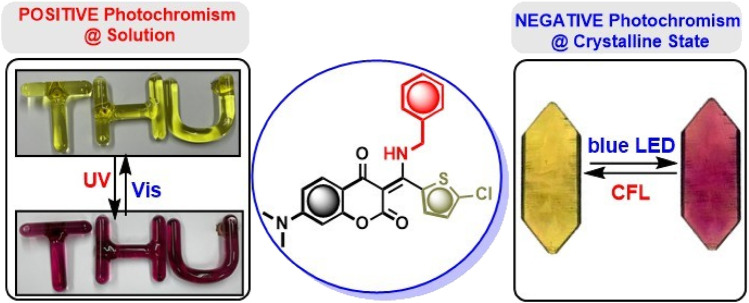

## 1 Introduction

**FIGURE 1:**
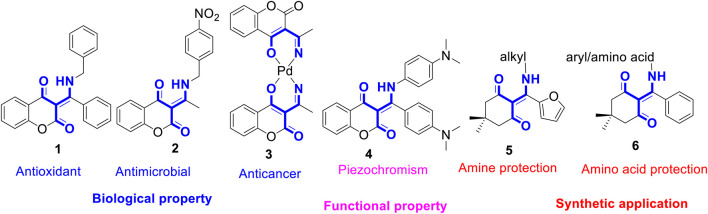
Structures of representative β-enamino diketones with biological/functional properties and synthetic applications.

The β-enamino diketones represent important and versatile synthons for the synthesis of natural products and other heterocycles (Michael et al., 2001; Valduga et al., 1998; Ferraz et al., 1995; White and Lhle, 2006; Li et al., 2007). Compounds bearing β-enamino diketone moiety, especially six-membered cyclic ones, were found to possess many biological properties. For instance, as shown in Figure 1, β-enamino diketone **1** is an antioxidant agent (Mladenovic et al., 2009). Compound **2** possesses strong inhibitory activity against fungus *C. albicans* (Vukovic et al., 2010). Compound **3** exhibits potent cytotoxicity against A546, HeLa, and K562 cells (Budzisz et al., 2004). Owing to their diverse biological activities, a plethora of multi-component reaction methodologies for the synthesis of β-enamino diketones have been reported in the literature (Kuo et al., 2009; Ghabraie et al., 2011; Ye et al., 2015). While most efforts were focused on the biological activities of the prepared compounds, their potential photochemical and functional properties were much less explored. Recently, we have reported that coumarin-based *N*-aryl-β-enamino diketone **4** (Figure 1) exhibits piezochromic properties (Hsieh et al., 2019); that is, upon grinding, compound **4** changes from yellow to red and can be swiftly reverted to the original color when exposed to methylene chloride vapor. Further, the furan-derived enaminones **5** serve as amine-protected compounds for primary alkyl amines protection. These acid/base stable amine-protected **5** can be readily deprotected by treating with ethylene diamine under reflux conditions (Chithanna and Yang, 2019). Similarly, the phenyl-derived enaminones **6** function as amine-protected products for aryl amines and amino acids in both solution and solid phase peptide synthesis. The free aryl amines and amino acids can be regenerated by treating with hydrazine hydrate in ethanol under mild conditions (Chithanna et al., 2020). These successful examples prompted us to speculate that new or unique functional behavior of β-enamino diketones via structural tuning of their three major molecular scaffolds, namely, cyclic diketone, aryl/alkyl amine, and aryl group. Further, we envisioned that the β-enamino diketones are prone to undergo the excited state intramolecular proton transfer (ESIPT) process upon photoirradiation by means of proton transfer from amine to nearby keto-group of coumarin. Thus, in our continuous efforts to unearth novel functional properties from the heterocycles synthesized via multi-component reaction, herein we reported the preparation of a library of β-enamino diketones from a microwave-assisted, three-component reaction of 4-hydroxycoumarins with various aromatic/aliphatic amines and β-nitrostyrenes in toluene under basic conditions. Their stimuli responses towards UV and visible light in solution, thin film, and crystalline state were examined. Further, the photochromic compound prepared from 7-*N*,*N*-dimethylamino-4-hydroxycoumarin, benzylamine, and 5-chlorothiophene-2-carbaldehyde was further linked to a structure-related, pressure-sensitive β-enamino diketone scaffold to investigate its potential multi-stimuli responsive properties.

## 2 Results and discussion

**SCHEME 1:**
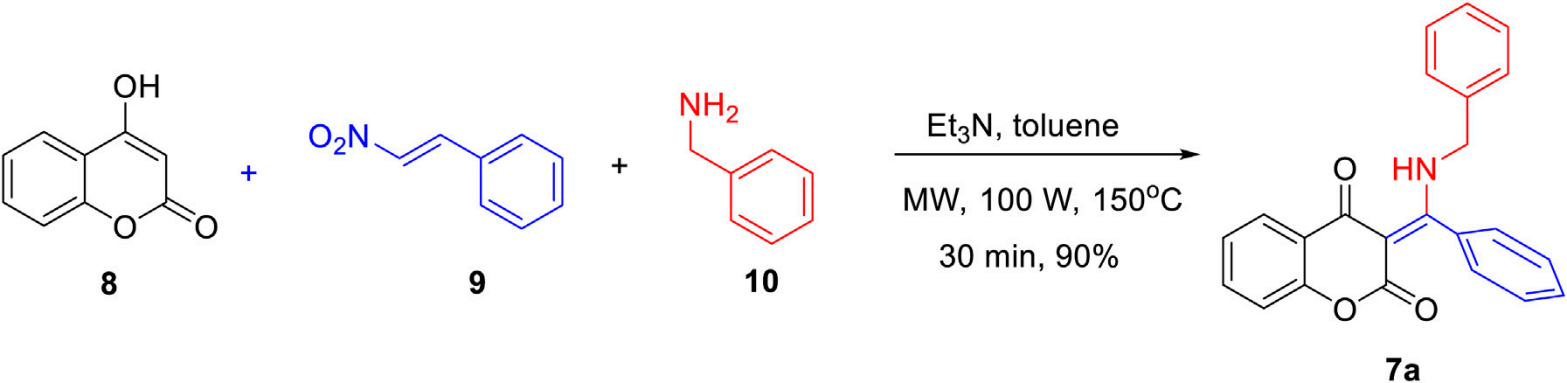
Three-component synthesis of β-enaminone diketone **7a**.

Scheme 1 outlines the microwave-assisted, three-component preparation of β-enamino diketone **7a**. With slight modifications from the literature reported procedure (Manjappa et al., 2018; Hsieh et al., 2019), it could be easily obtained via triethylamine-mediated coupling of 4-hydroxycoumarin (**8**), β-nitrostyrene (**9**), and benzylamine (**10**) in toluene under microwave conditions for 30 min in 90% yield.

**FIGURE 2:**
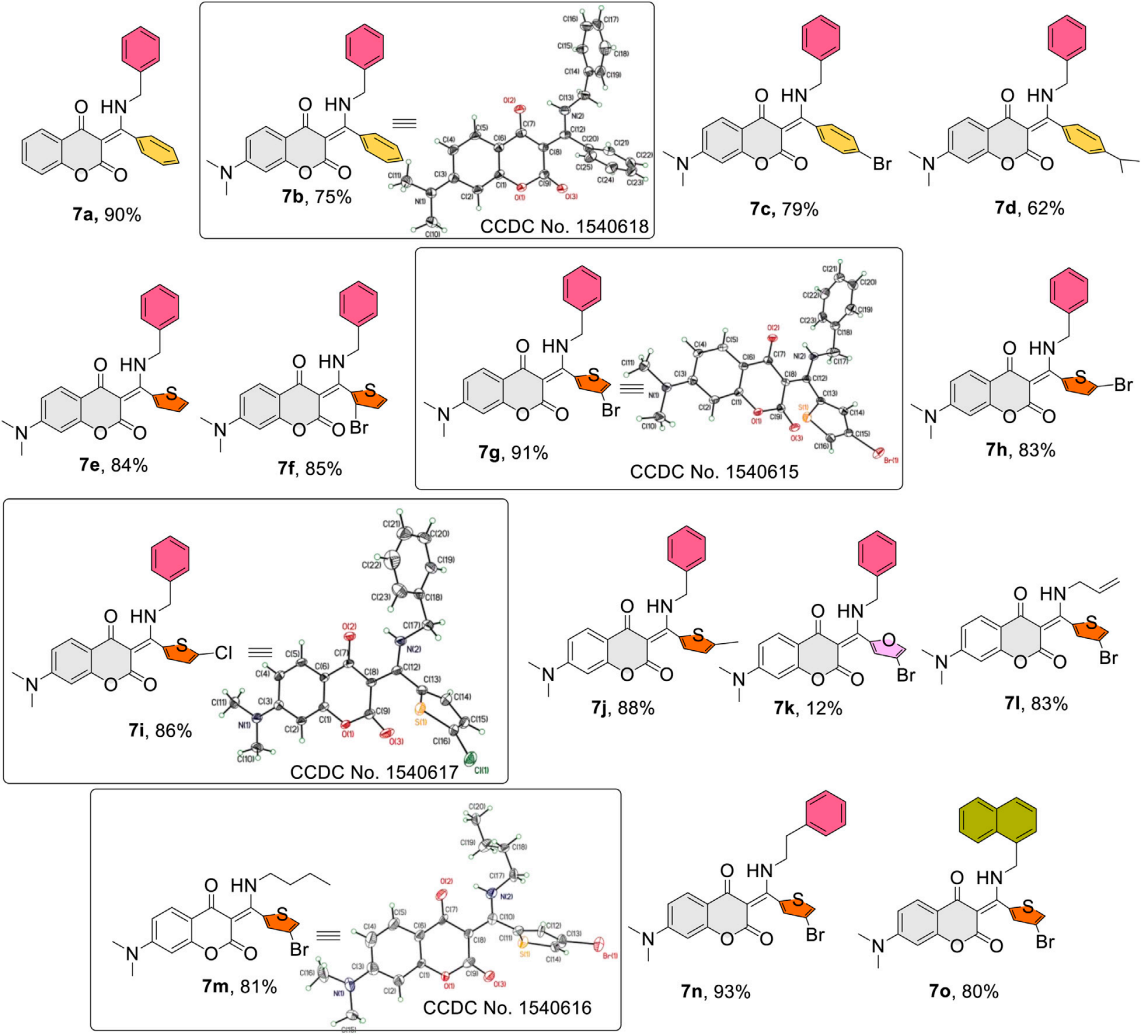
Structures and yields of the prepared β-enamino diketones **(7a–o)** and ORTEP crystal structures of **(7b, 7g, 7i, 7m)** with atomic displacement shown at 50% probability.

Figure 2 lists the structures and yields of the prepared **7a**–**o**. Most of the reactions gave good to excellent yields, except for compound **7k** in which 4-bromofuran-2-carbaldehyde was used as the aldehyde source. This observation could be attributed to the furan’s high propensity to be attacked by a nucleophilic amine (Helmy et al., 2014; Chithanna and Yang, 2019). In the present investigation, we focused mainly on 7-*N*,*N*-dimethylamino-4-hydroxycoumarin, benzylamine, and thiophene aldehydes derived β-enaminone diketones. The molecular structures of diketones **7a**–**o** were elucidated by spectroscopic data along with X-ray crystal analysis (**7b**, **7g**, **7i**, and **7m**) (CCDC No, 2023). A broad signal appearing around 13–14 ppm in the proton NMR spectrum was detected for all prepared compounds, indicating the presence of an intramolecular hydrogen bond in the molecular scaffold. Indeed, an intramolecular hydrogen bonding between the amine hydrogen (*N*-H) and carbonyl oxygen (C=O) of coumarin was distinctly observed in the ORTEP diagrams shown in Figure 2. Generally, changes of amines (aryl or aliphatic) or aromatic/heterocyclic aldehydes had little effect on product yields.

**FIGURE 3:**
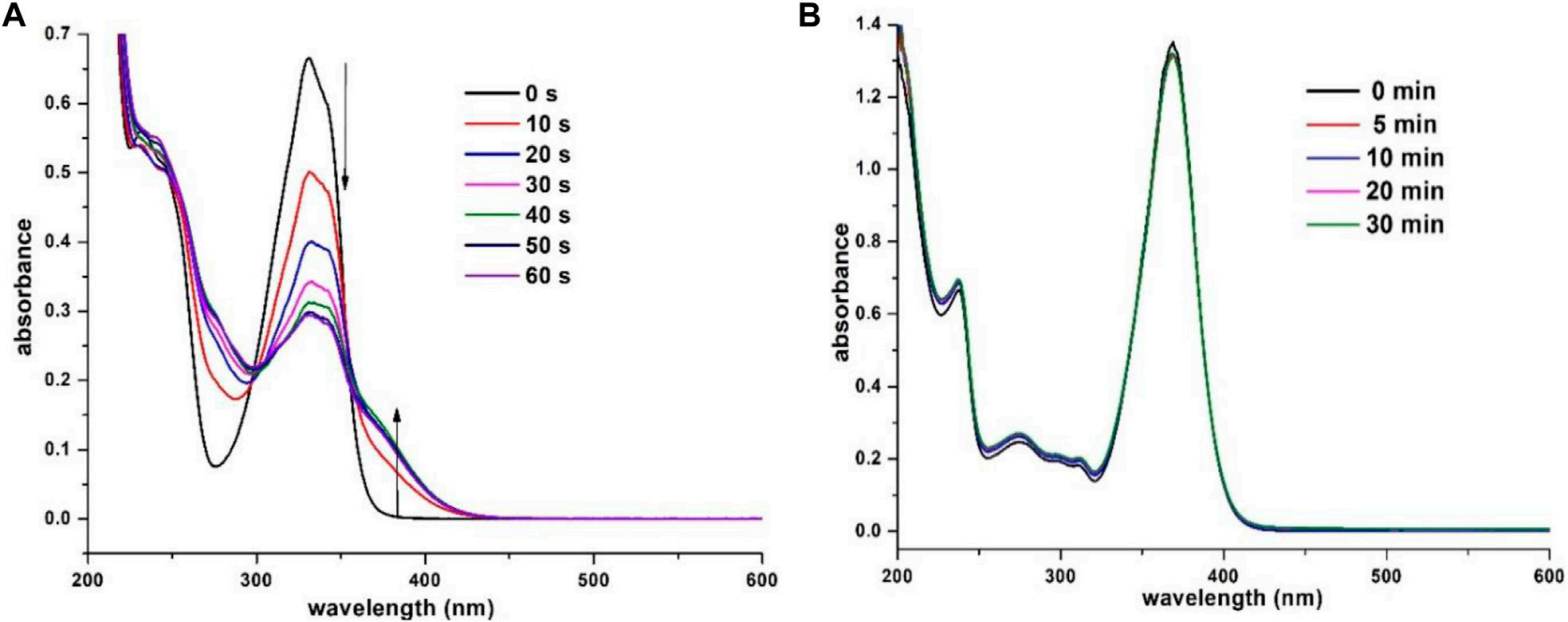
Absorption spectra of **(A) 7a** (3.0 × 10^−5^ M in CH_3_CN) and **(B) 7b** (3.0 × 10^−5^ M in CH_3_CN) obtained with different exposure times (352 nm).

**SCHEME 2:**
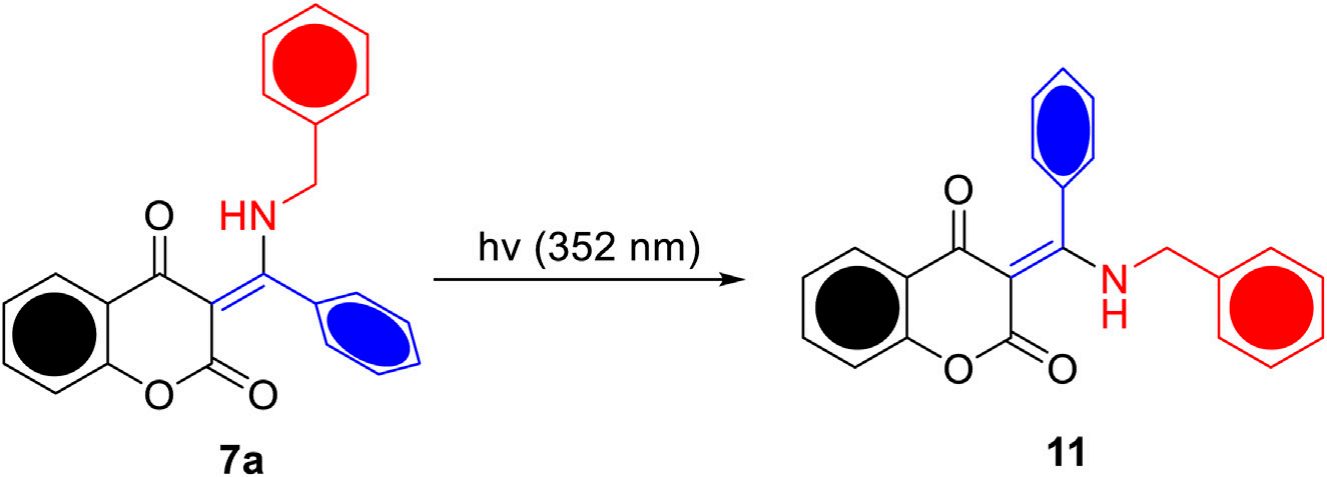
Proposed *E*-*Z* isomerization between **7a** and **11**.

To investigate the potential ESIPT behavior, the prepared β-enaminone diketones **7a**–**o** were subjected to photoirradiation and their photochemical properties were explored. Compound **7a**, in which benzaldehyde was used as the aldehyde substrate, was found to be sensitive to UV light. Figure 3 shows the absorption spectra of **7a** in acetonitrile when exposed to UV irradiation (352 nm) for 60 s. With the increase of exposure time, the absorbance at wavelength of 330 and 342 nm gradually decreased, along with the emergence of broad shoulder at around 380 nm. Also, two isosbestic points located at 305 and 355 nm were clearly observed. Presumably, upon UV irradiation, compound **7a** undergoes *E*-*Z* isomerization of the central carbon-carbon double bond to generate the isomer **11**, as shown in Scheme 2. To further support this hypothesis, a time-dependent proton NMR study was carried out. Prior to irradiation, a small doublet appeared at 4.42 ppm and a broad singlet appeared at 14.34 ppm which correspond to the respective benzylic -CH_2_ and the intramolecular hydrogen bonding absorptions were observed in the proton NMR spectrum of **7a**. During the process of UV irradiation (up to 60 min), these peaks gradually diminished and the concomitant emergence of two new peaks at 4.83 and 9.03 ppm which correspond to the benzylic -CH_2_ and intramolecular hydrogen bonding absorptions of **11** was witnessed (see the Supporting Information). These observations suggest that indeed compound **7a** undergoes *E*-Z isomerization to give the compound **11** upon UV irradiation. On the other hand, when an *N*,*N*-dimethylamino group was introduced onto the 7-position of the coumarin moiety, the resulting compound **7b** became light-insensitive. Prolonged irradiation of **7b** did not exhibit considerable changes in the UV-vis absorption profiles (Figure 3B). This observation implies that the photochemical property of the β-enaminone diketones can be influenced by the substituents on the coumarin moiety.

**FIGURE 4:**
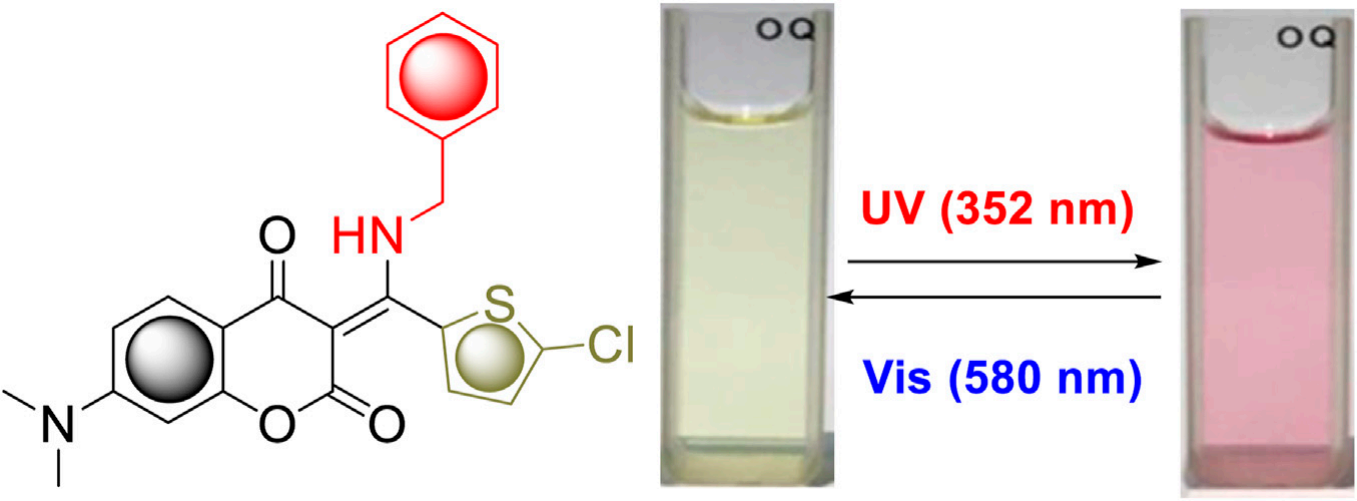
Photochromic behavior of **7i** in acetonitrile.

**FIGURE 5:**
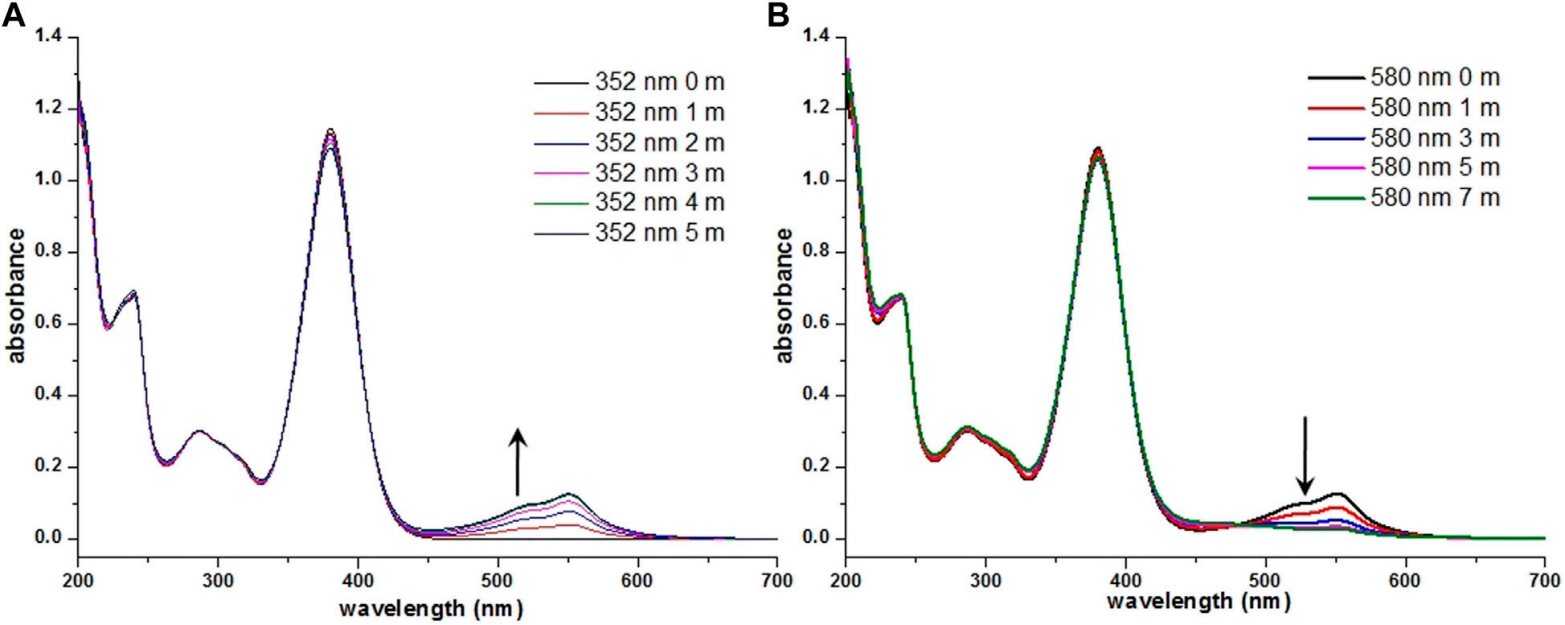
Absorption spectra of **(A) 7i** (3.0 × 10^−5^ M in CH_3_CN) obtained with different exposure times (352 nm), 0–5 min, in increments of 1 min **(B)** the photogenerated product obtained with different exposure times (580 nm), 0–7 min, in increments of 2 min.

Interestingly, compound **7i** bearing a 5-chlorothiophene moiety was found to be highly sensitive to UV light and exhibit unique photochemical properties. As shown in Figure 4, it turned from light yellow to red within seconds when exposed to UV irradiation in solution. Figure 5A displays the time course of the UV–vis absorption spectra of **7i** in acetonitrile under continuous irradiation (352 nm) for 5 min. With the increase of exposure time, two broad absorbance peaks which centered at 527 and 551 nm exhibited smooth continuous growth. This process could be reverted by visible light irradiation (580 nm), indicating that compound **7i** possesses photochromic property (Figure 5B).

**FIGURE 6:**
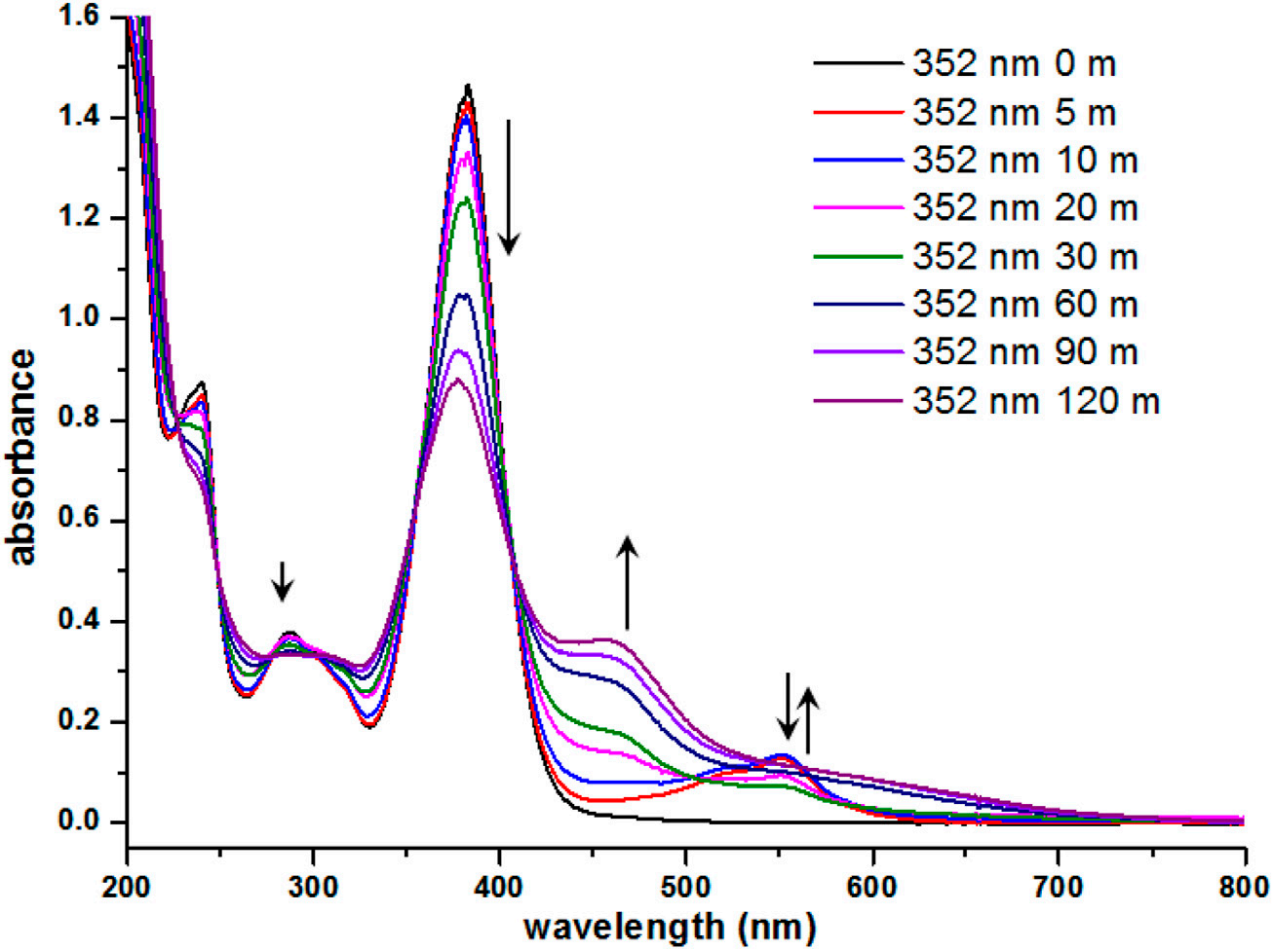
UV-vis spectra of **7i** (3.0 × 10^−5^ M in CH_3_CN) after continuous irradiation at 352 nm for 120 min.

Isolation of the photogenerated product and subsequent characterization of its molecular structure proved to be difficult since prolonged UV irradiation of **7i** resulted in the *E*-*Z* isomerization to be the dominant process. Figure 6 depicts the evolution of absorbance profile of **7i** during the prolonged UV irradiation. Starting from the initial 5 min of UV irradiation, there was a surge in a broad absorption band around 520–560 nm, indicating the formation of photogenerated products. However, as the irradiation time was further extended (up to 120 min), the decrease in absorption at 372 nm and the appearance of a broad shoulder near 455 nm were also recorded. This observation suggests that prolonged irradiation of compound **7i** renders the *E-Z* isomerization to be a dominant process over the formation of photogenerated product. Therefore, current efforts to characterize the photogenerated product resulting from this photochromic behavior were futile.

**FIGURE 7:**
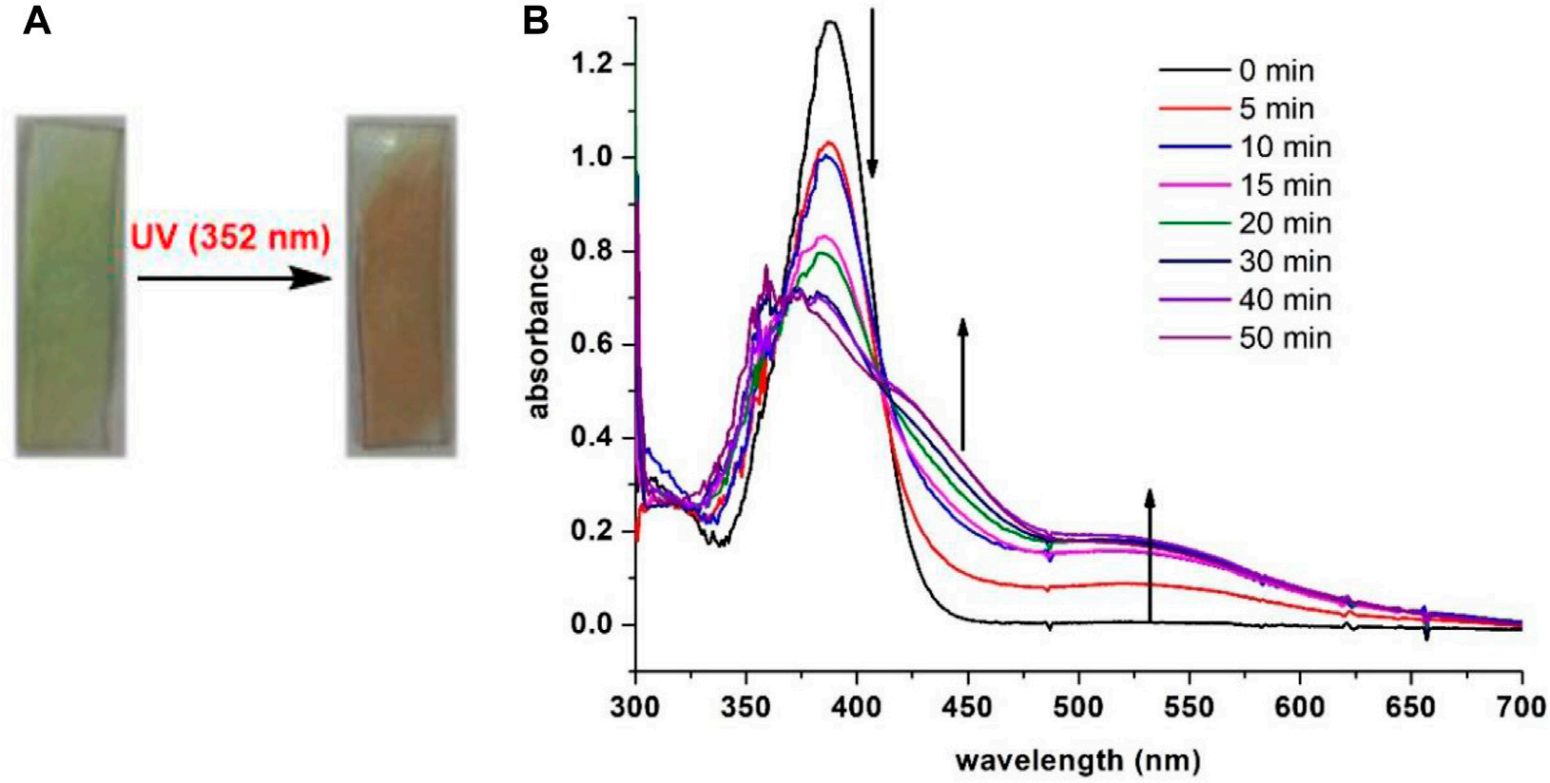
**(A)** Color change prior to and after UV irradiation of **7i** (thin film). **(B)** UV-vis spectra of **7i** (spin-coated on quartz plate) after continuous irradiation at 352 nm for 50 min.

Inspired by the photochromic behavior in solution, we chose to investigate the photosensitivity of **7i** in solid phase. The thin film of **7i** was prepared by spin coating on a quartz plate and then irradiated with light. When exposed to blue LED for 60 min, no apparent color change of **7i** was observed. Conversely, compound **7i** (thin film) changed from colorless to red in 5 min upon UV (352 nm) irradiation. Figure 7 shows the color change and absorption profile of **7i** (thin film) prior to and after irradiation. As UV exposure increased, the absorbance centered at 388 nm decreased along with a smooth enhancement of the shoulders near 450 nm and 530 nm. This evolution of the absorbance resulted in the formation of two isosbestic points at 366 and 412 nm, which indicates the presence of two distinguishable species in the film. The fact that UV-vis spectra of **7i** in thin film (solid state) exhibited strong resemblance to that of **7i** in solution phase (Figure 6) implies that similar photochromic mechanisms may be involved for **7i** in solution and solid state.

**FIGURE 8:**
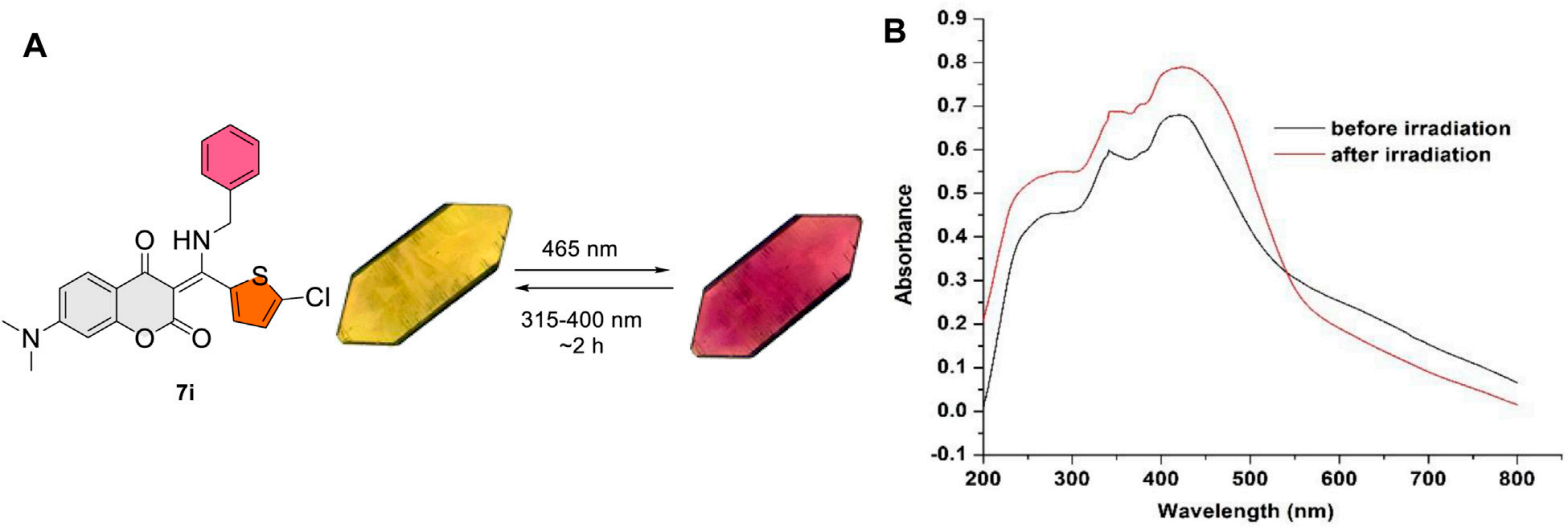
**(A)** Reversible color change in crystalline state for **7i**. **(B)** UV-vis absorption profiles of **7i** prior to and after blue LED irradiation (40 min).

After discovering the photochromism of **7i** in both solution and solid state, we embarked upon our investigations on its photochromic behavior in crystalline state (Avadanei et al., 2014; Irie et al., 2014; Funasako et al., 2019; Zhang et al., 2021; Sun et al., 2022; Loan et al., 2023). A fine, moderate-sized crystal of **7i** was grown and subjected to photoirradiation. To our surprise, **7i** was found to be highly sensitive to visible light in crystalline state. When exposed to blue LED (465 nm), the crystal of **7i** changed from yellow to violet within 15 s. Gratifyingly, this process was reversible upon exposure to the compact fluorescent lamp (CFL) for 2 h. Figure 8 shows the color transition of **7i** at crystalline state as well as UV-vis absorption profiles of **7i** prior to and after the blue LED irradiation. Before the exposure, **7i** displayed a maximum absorbance (λ_max_) around 416 nm. After blue LED exposure (40 min), the λ_max_ was red-shifted to 425 nm along with the broad absorbance increase from 425 to 550 nm. This change in the absorbance was visible to the naked eye as blue LED imparted dark violet color to the **7i** crystal. The violet crystal of **7i** gradually returned to yellow upon exposure to CFL light (315–400 nm for UVA), suggesting possible reversibility. In the case of color change of **7i** in solution photochromism, the number of cycles is limited to 3–4 times only. On the other hand, the number of photochromic cycles for **7i** in crystalline state is up to 25 times (see the Supplementary Material for details), which is comparatively higher than that of in solution state.

**FIGURE 9:**
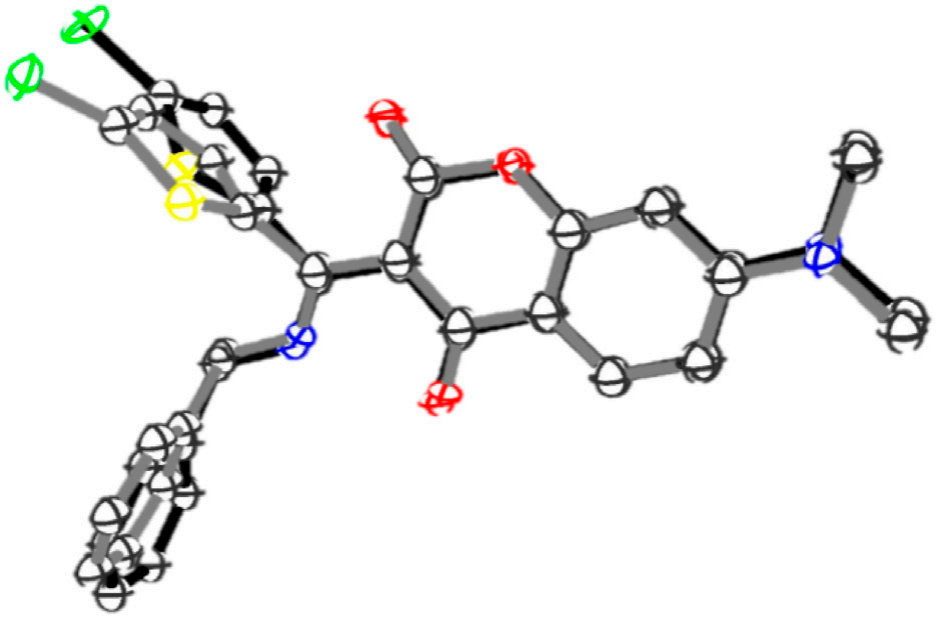
Superimposition of the ORTEP diagrams of **7i** before (black) and after (grey) blue LED irradiation. Hydrogens are omitted for clarity.

In order to gain more insights into the photosensitivity of **7i** towards blue LED, the X-ray crystallography was sought to examine the possible structural changes at crystalline state during irradiation. A crystal of **7i** was irradiated with blue LED for 24 h and its X-ray ORTEP diagram was obtained (CCDC No 2300435). Figure 9 depicts the superimposition of the ORTEP diagrams of **7i** before and after blue LED irradiation. Some conformational differences in the crystals were noticed, that is, the benzylamine and thiophene moieties were found to be tilted to certain degrees after irradiation. For instance, prior to the blue LED irradiation, the dihedral angle between coumarin and thiophene moieties in **7i** was found to be −81.82^o^. After irradiation, however, it changed to −70.79^o^. Similarly, the torsion angle between coumarin and benzylamine moieties switched from 34.35^o^ to 56.09^o^ after irradiation. These subtle yet significant conformational variations during blue LED irradiation suggested that the compound **7i** is indeed sensitive to visible light and is capable of undergoing reversible changes upon irradiation with suitable wavelength. Although the mechanistic details for the photochromic switch of **7i** in solution, thin film, and crystalline state remain to be investigated, compound **7i** represents one of the rare examples that exhibit opposite photochromic property under different states (Funasako et al., 2020), that is, positive photochromism in solution and thin film as well as negative photochromism in crystalline state.

**SCHEME 3:**
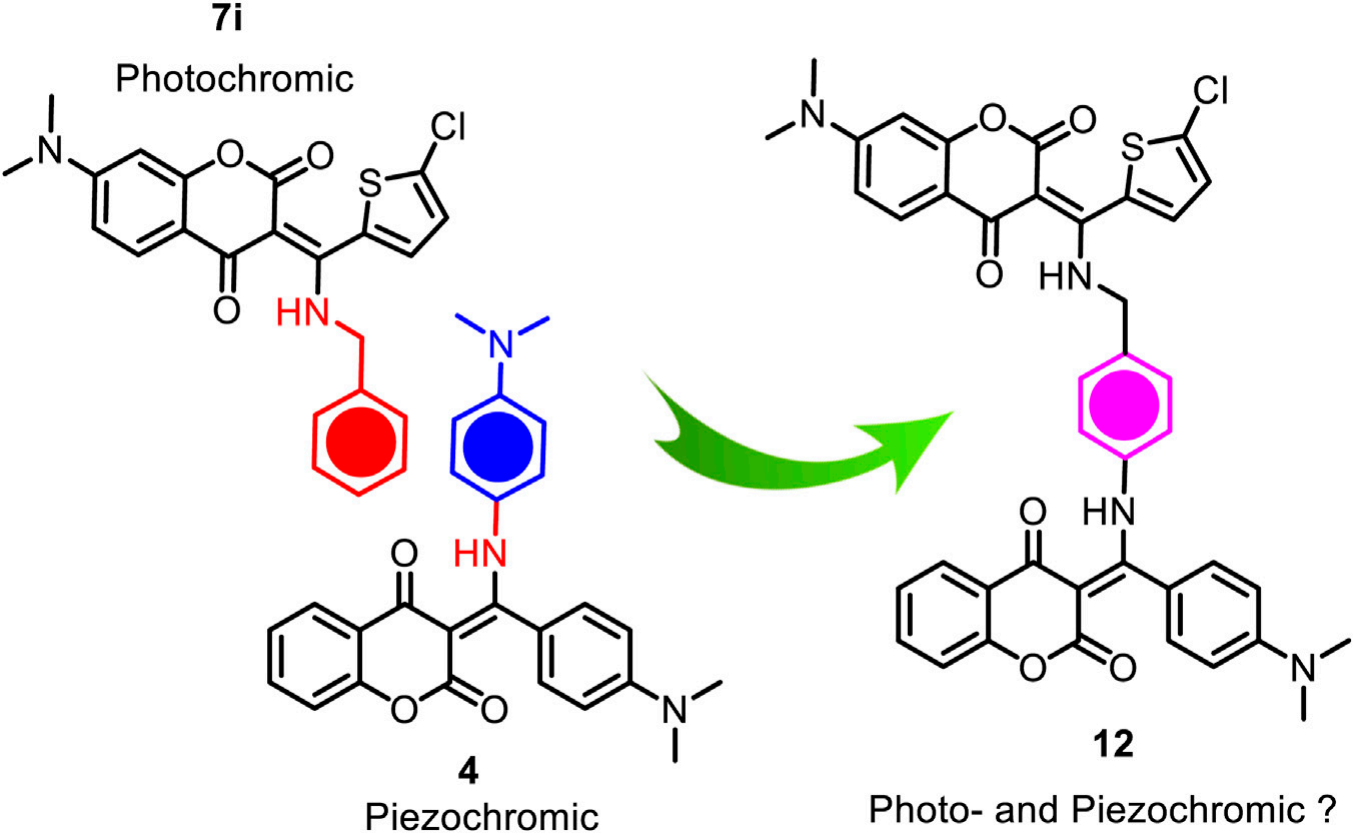
Conceptual design of the hybrid **12**.

As illustrated in Figure 1, our previous study has demonstrated that coumarin-based *N*-aryl-β-enamino diketone **4** exhibits piezochromic behavior (Figure 1) (Hsieh et al., 2019). We speculate that by linking this pressure-sensitive molecular scaffold (Sagara et al., 2007; Sagara and Kato, 2009; Ma et al., 2015; Naumov et al., 2015; Wang et al., 2015; Li et al., 2021; Wei et al., 2023) of **4** with the present light-sensitive moiety of **7i** into one molecule, the resulting hybrid compound may exhibit multi-stimuli responsive properties in solid state (Zhang et al., 2011; Chiu and Yang, 2020; Vaidya et al., 2021; Martinez-Junquera et al., 2022; Wang et al., 2022). Scheme 3 depicts the conceptual design of the potential dual responsive *N*-aryl-β-enamino diketone **12**. Since both piezochromic **4** and photochromic **7i** share a phenyl group on the *N*-substituent, this common phenyl group was then used to connect the two molecular scaffolds to form the hybrid **12**.

**SCHEME 4:**
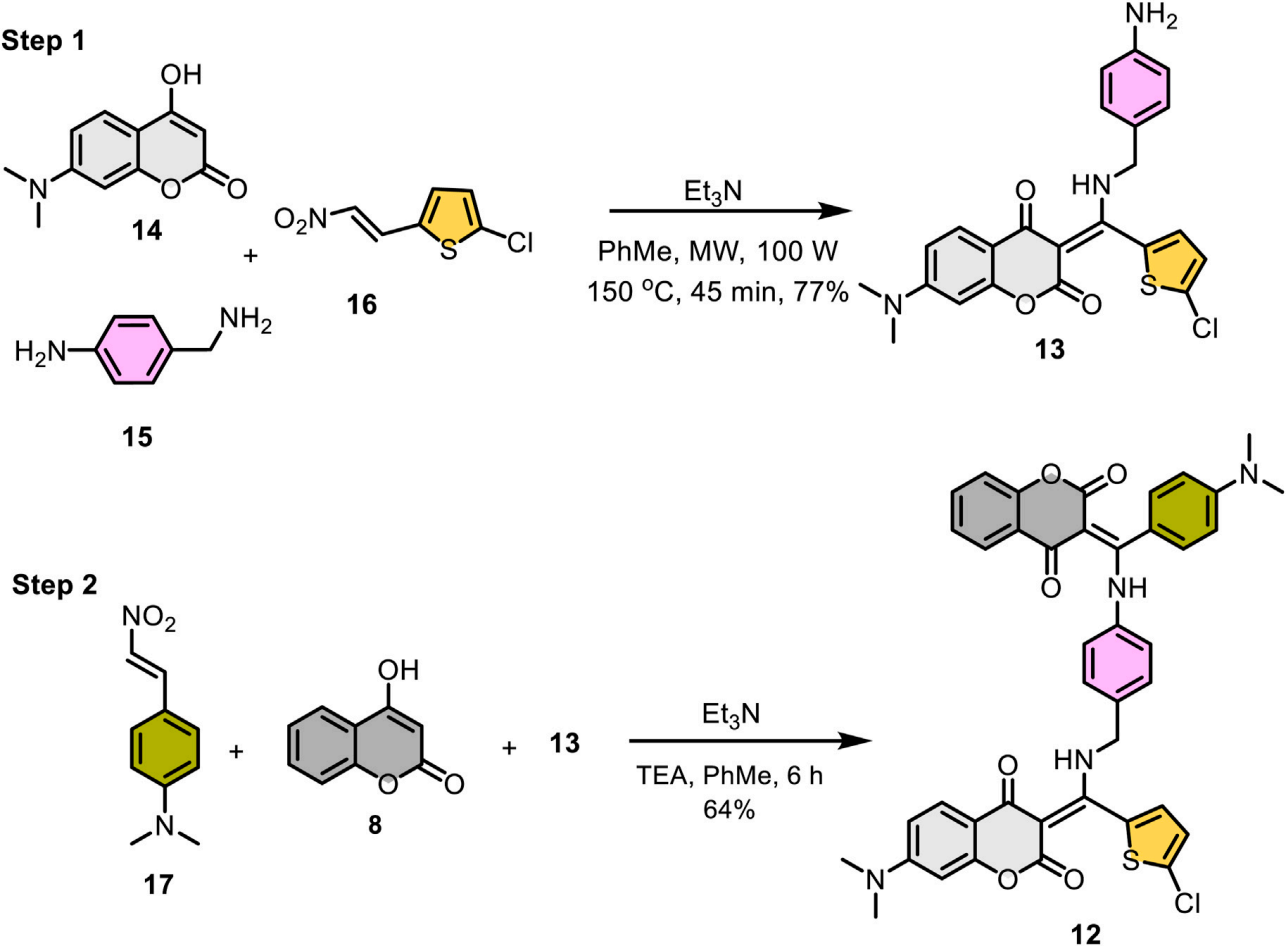
Two-step preparation of β-enamino diketone **12**.

Scheme 4 outlines the two-step synthesis of the target compound **12**. The photochromic unit **13** was synthesized via base-promoted, three-component reaction of 7-*N*,*N*-dimethylamino-4-hydroxycoumarin (**14**), 4-aminobenzylamine (**15**), and β-nitrostyrene **16** under microwave conditions. As expected, the less nucleophilic aniline nitrogen on 4-aminobenzylamine (**15**) did not participate in the reaction, and the desired β-enamino diketone **13** was isolated as an exclusive product in 77% yield. The subsequent three-component reaction of **13** with 4-hydroxycoumarin (**8**) and *p*-*N*,*N*-dimethylamino-β-nitrostyrene (**17**) under basic conditions yielded the hybrid β-enaminone **12** in 64% yield.

**FIGURE 10:**
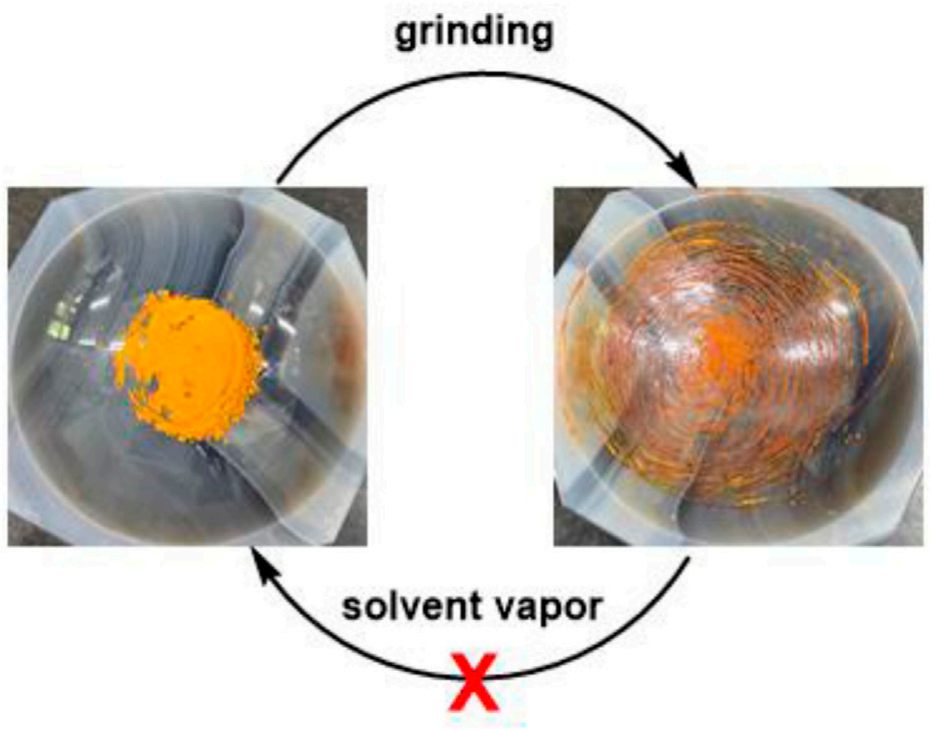
Irreversible response of compound **12** towards mechanical force.

After realizing compound **12**, we then explored its functional properties by applying external stimuli such as mechanical force and UV irradiation to examine its potential piezochromic and photochromic responses. Upon grinding, compound **12** turned slowly from yellow to dark brown (Figure 10), indicating that the hybrid **12** remained pressure-sensitive. Nevertheless, the ground **12** failed to return to its original color when exposed to various solvent vapors such as methylene chloride, dichloroethane, acetone, chloroform, and THF, etc. This irreversible response of the hybrid **12** towards mechanical force suggests that it is not piezochromic anymore.

**FIGURE 11:**
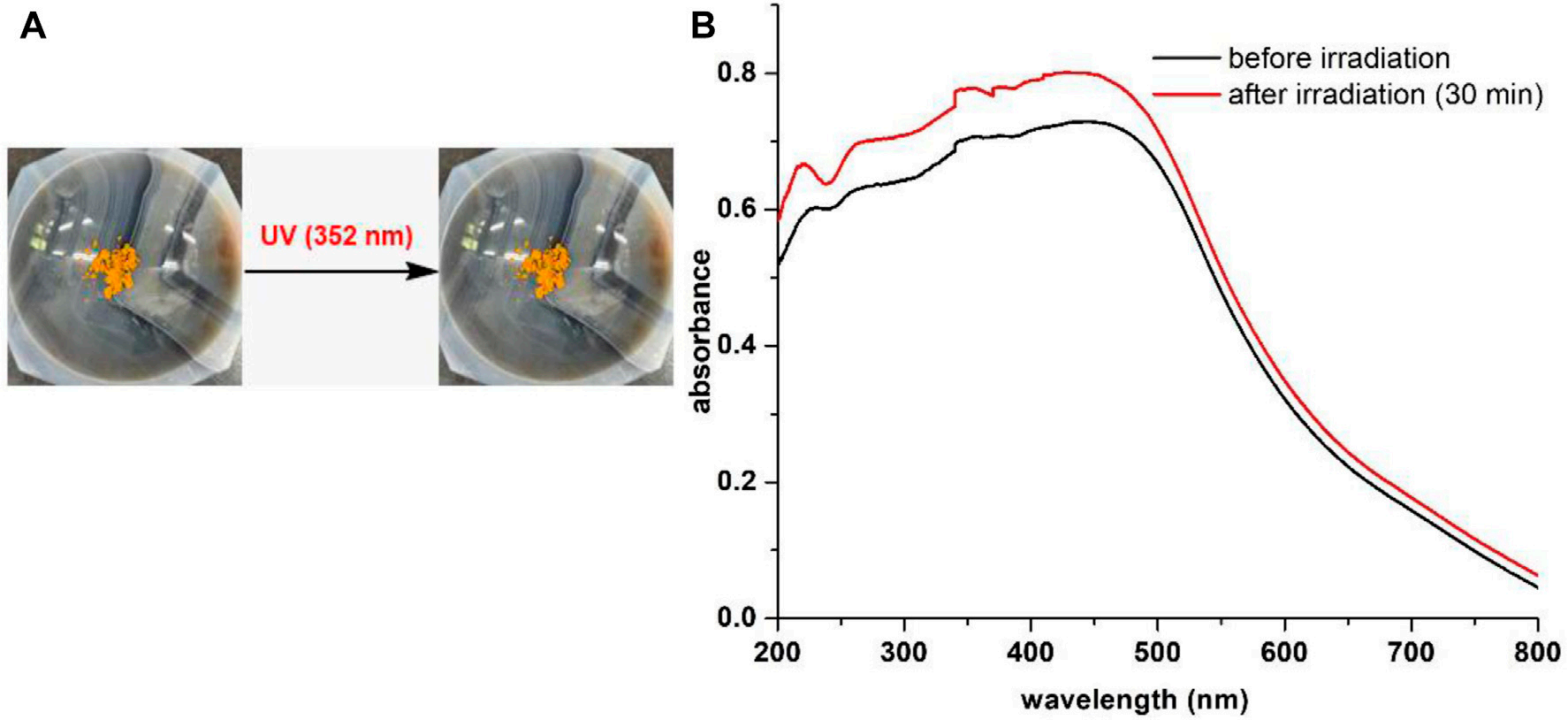
**(A)** No color change of **12** prior to and after UV (352 nm) irradiation. **(B)** Solid state absorption profiles of **12** prior to and after UV (352 nm) irradiation.

Compound **12** was also subjected to UV (352 nm) irradiation in solid state as it consisted of a light-sensitive β-enamino diketone moiety. Regrettably, the hybrid **12** failed to respond to UV light in solid state. As shown in Figure 11A compound **12** did not show noticeable color change even after exposed to UV light (352 nm) for 30 min. Further, no major change was observed in the solid-state absorbance spectra of **12** prior to and after UV irradiation (Figure 11B). Our studies suggest that a multi-stimuli responsive molecule cannot be constructed simply through combination of two different chromic moieties into one, even though structures of the two chromic molecular scaffolds are closely related.

## 3 Methods and materials

### 3.1 General information

Microwave reactions were performed using a CEM Discover unit (operating at 110 V, microwave irradiation of 2.45 GHz, maximum microwave output of 300 W) in 50 mL capacity open round-bottom flasks. Visualization was accomplished by using portable UV light and an iodine chamber. Flash chromatography was performed in columns of various diameters with Merck silica gel (230–400 mesh ASTM 9385 kieselgel 60H) by elution with the solvent systems. Solvents, unless otherwise specified, were reagent grade and distilled once before use. All new compounds exhibited satisfactory spectroscopic and analytical data. ^1^H NMR (400 MHz) and ^13^C NMR (100) spectra were recorded on a Bruker 400 spectrometer. Chemical shifts were reported in parts per million on the scale relative to an internal standard (tetramethylsilane, or appropriate solvent peaks) with coupling constants given in hertz. ^1^H NMR multiplicity data are denoted by s (singlet), d (doublet), t (triplet), q (quartet), and m (multiplet). Analytical thin-layer chromatography (TLC) was carried out on Merck silica gel 60G-254 plates (25 mm) and developed with the solvents mentioned. Melting points were determined on a Mel-Temp melting point apparatus in open capillaries and are uncorrected. High-resolution mass spectra (HRMS) were obtained on a Thermo Fisher Scientific Finnigan MAT95XL spectrometer using a magnetic sector analyzer. Infrared (IR) spectra were recorded using 1725XFT-IR spectrophotometer. Single-crystal structures were determined with a Bruker AXS SMART-1000 X-ray single-crystal diffractometer. The absorption spectra were obtained using a UV/vis/NIR spectrophotometer (Jasco V-770) with a deuterium lamp (190–350 nm) and halogen lamp (300–2,700 nm) light sources and the detector was a photomultiplier tube.

### 3.2 Synthesis of compounds 7a–o, 12, and 13

Mixtures of appropriately substituted 4-hydroxycoumarin (1 equiv.), β-nitrostyrene (1.2 equiv.), substituted amine (1.2 equiv.), and a few drops of triethylamine in toluene (∼20 mL) were irradiated under microwave (100 W, 150 C) for 25–30 min (unless otherwise specified). The cooled reaction mixture was concentrated and then re-dissolved in DCM. The solution was washed with water, brine, dried over MgSO_4_, and evaporated *in vacuo*. The crude product was purified by column chromatography and was further recrystallized from DCM/hexanes.

## 4 Conclusion

In summary, a total of 15 structurally diverse β-enamino diketone derivatives were synthesized in good to excellent yields via microwave-assisted, base-mediated, three-component reaction between 4-hydroxycoumarins, substituted β-nitrostyrene, and primary amines. Among prepared compounds, compound **7i** was found to exhibit positive photochromism in solution/thin film and negative photochromism in crystalline state. Further, compound **12** bearing pressure- and light-sensitive molecular scaffolds was designed and synthesized in two steps as a potential dual-responsive material. Unfortunately, the prepared **12** failed to show any expected functional behavior. The in-depth investigation of the photochromic mechanism of this intriguing thiophene-derived β-enamino diketone **7i** at the molecular level is currently underway and will be reported in due course.

## Data Availability

The datasets presented in this study can be found in online repositories. The names of the repository/repositories and accession number(s) can be found in the article/Supplementary Material.
