# Supplementary material for: Structural tuning of β-enamino diketones: exploration of solution and crystalline state photochromism
Source: Front Chem. 2023 Nov 7;11:1295347. doi: 10.3389/fchem.2023.1295347 (PMC10661371; doi:10.3389/fchem.2023.1295347)
Supplement: Supplementary file 1 [file DataSheet1.PDF]

Structural Tuning of β-Enamino Diketones: Exploration of Solution and Crystalline State Photochromism

Kiran B. Manjappa,[1]* Sheng-Chieh Fan,[2] Ding-Yah Yang,[1,2]*

[1]Graduate Program for Biomedical and Materials Science, Tunghai University
[2]Department of Chemistry, Tunghai University
No. 1727, Sec. 4, Taiwan Boulevard, Xitun District, Taichung 407224, Taiwan

*** Correspondence:**
Kiran B. Manjappa: kiran@thu.edu.tw
Ding-Yah Yang: yang@thu.edu.tw

Supporting Information

Table of Contents

1. Characterization of prepared compounds S2–S7
2. Single X-ray crystal structure analysis of the compounds **7b**, **7g**, **7i**, and **7m** S8–S15
3. Crystalline state reversible color change of the compound **7i**........ S16
4. UV-absorbance profile of the prepared compounds S17–S24
5. ^{1}H and ^{13}C NMR spectra of prepared compounds S25–S65

1. Characterization of prepared compounds

7a; R_f = 0.58 (30% EtOAc/hexanes); colorless solid; 197 mg; yield 90%; mp 216–218 °C;

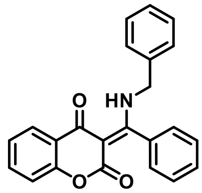

^{1}H NMR (400 MHz, $CDCl_3$) δ 14.44 (b.s., 1H), 8.07 (d, *J* = 7.6 Hz, 1H), 7.55–7.51 (m, 5H), 7.37–7.29 (m, 3H), 7.25–7.19 (m, 5H), 4.37 (d, *J* = 6.0 Hz, 2H); ^{13}C NMR (150 MHz, $CDCl_3$) δ 194.1, 173.0, 170.3, 138.8, 135.2, 132.3, 130.6, 129.1, 128.8, 128.2, 127.8, 127.5, 125.6, 124.4, 123.0, 120.6, 119.0, 114.0, 46.3; IR ν_{max} (KBr) 1708, 1559, 1341, 1231, 1068, 898, 703 cm^{-1}; HRMS (EI) m/z calcd for $C_{23}H_{17}NO_3$ [M^+] 355.1208, found 355.1213.

7b: R_f = 0.36 (30% EtOAc/hexanes); off–brown solid; 130 mg; yield 67%; mp 142–144

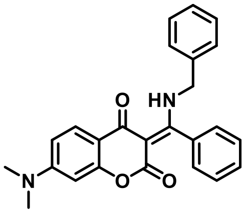

°C; ^{1}H NMR (400 MHz, $CDCl_3$) δ 14.42 (b.s., 1H), 7.88 (d, *J* = 8.8 Hz, 1H), 7.49–7.47 (m, 3H), 7.36–7.29 (m, 3H), 7.25–7.23 (m, 2H), 7.17 (d, *J* = 7.2 Hz, 2H), 6.56 (dd, *J* = 9.2, 2.4 Hz, 1H), 6.29 (*J* = d, 2.4 Hz, 1H), 4.33 (d, *J* = 6.4 Hz, 2H), 3.04 (s, 6H); ^{13}C NMR (100 MHz, $CDCl_3$) δ 181.9, 174.7, 162.1, 156.2, 154.6, 136.1, 133.9, 129.2, 128.9, 128.8, 127.9, 127.3, 127.1, 125.9, 109.4, 108.2, 97.2, 96.0, 48.9, 40.1; IR ν_{max} (KBr) 1715, 1610, 1539, 1436, 1337, 1127, 1067, 905 cm^{-1}; HRMS (EI) m/z calcd for $C_{25}H_{22}N_2O_3$ [M^+] 398.1630, found 398.1635.

7c: R_f = 0.52 (30% EtOAc/hexanes); off–brown solid; 184 mg; yield 79%; mp 214–216

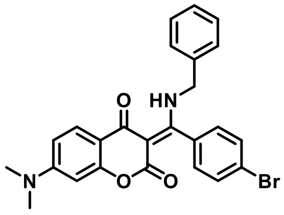

°C; ^{1}H NMR (400 MHz, $CDCl_3$) δ 14.43 (b.s., 1H), 7.87 (d, *J* = 8.8 Hz, 1H), 7.61 (d, *J* = 8.4, 2H), 7.36–7.29 (m, 3H), 7.16 (d, *J* = 6.8 Hz, 2H), 7.11 (d, *J* = 8.4 Hz, 2H), 6.57 (dd, *J* = 8.8, 2.4 Hz, 1H), 6.29 (d, *J* = 2.4 Hz, 1H), 4.32 (d, *J* = 6.0 Hz, 2H), 3.05 (s, 6H); ^{13}C NMR (150 MHz, $CDCl_3$) δ 181.9, 173.6, 162.2, 156.1, 154.6, 135.9, 132.8, 132.1, 129.0, 128.1, 127.7, 127.2, 127.1, 123.6, 109.2, 108.3, 97.2, 96.0, 48.9, 40.1; IR ν_{max} (KBr) 1709, 1611, 1539, 1439,1334, 1231, 1124, 1065, 907, 824, 697 cm^{-1}; HRMS (EI) m/z calcd for $C_{25}H_{21}BrN_2O_3$ [M^+] 476.0736, found 476.0743.

7d: R_f = 0.51(30% EtOAc/hexanes); coloress solid; 133 mg; yield 62%; mp 182–184 °C;

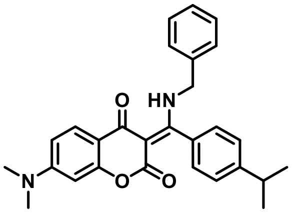

^{1}H NMR (400 MHz, $CDCl_3$) δ 14.37 (b.s., 1H), 7.87 (d, *J* = 9.2 Hz, 1H), 7.35–7.28 (m, 5H), 7.17 (t, *J* = 7.2 Hz, 4H), 6.56 (d, *J* = 8.8 Hz, 1H), 6.29 (s, 1H), 4.34 (d, *J* = 6.0 Hz, 2H), 3.04 (s,

6H), 3.03–2.96 (m, 1H), 1.31 (d, *J* = 2.8 Hz, 6H); ^{13}C NMR (100 MHz, $CDCl_3$) δ 181.8, 175.1, 162.2, 156.2, 154.5, 150.0, 136.2, 131.6, 128.9, 127.9, 127.3, 126.8, 126.0, 109.4, 108.2, 97.1, 96.1, 48.9, 40.0, 33.9, 30.8, 23.8; IR ν_{max} (KBr) 1713, 1363, 1336, 1232, 1123, 840, 694 cm^{-1}; HRMS (EI) m/z calcd for $C_{28}H_{28}N_2O_3$ [M^+] 440.2100, found 440.2102.

7e: R_f = 0.42 (40% EtOAc/hexanes); light yellow solid; 166 mg; yield 84%; mp 213–215 °C; ^{1}H NMR (400 MHz, $CDCl_3$) δ 14.28 (b.s., 1H), 7.85 (d, *J* = 9.2 Hz, 1H), 7.55 (d, *J* = 4.8 Hz, 1H), 7.38–7.28 (m, 3H), 7.23 (d, *J* = 7.2 Hz, 2H), 7.14 (t, *J* = 3.6 Hz, 1H), 7.04 (d, *J* = 3.2 Hz, 1H), 6.55 (dd, *J* = 6.8, 2.0 Hz, 1H), 6.30 (d, *J* = 2.0 Hz, 1H), 4.49 (d, *J* = 6.0 Hz, 2H), 3.05 (s, 6H); ^{13}C NMR (100 MHz, $CDCl_3$) δ 180.9, 167.9, 156.0, 154.6, 136.1, 133.1, 129.0, 128.8, 128.0, 128.0, 127.3, 127.2, 125.9, 109.3, 108.3, 108.2, 97.6, 97.1, 49.0, 40.1; IR ν_{max} (KBr) 2920, 1705, 1616, 1543, 1350, 1119, 1065, 833 cm^{-1}; HRMS (EI) m/z calcd for $C_{23}H_{20}N_2O_3S$ [M^+] 404.1195, found 404.1187.

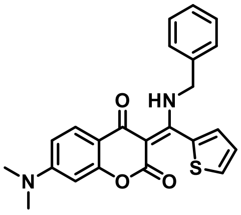

7f: R_f = 0.51 (40% EtOAc/hexanes); light yellow solid; 200 mg; yield 85%; mp 160–162 °C; ^{1}H NMR (400 MHz, $CDCl_3$) δ 14.39 (b.s., 1H), 7.86 (d, *J* = 9.2 Hz, 1H), 7.49 (d, *J* = 5.2 Hz, 1H), 7.38–7.27 (m, 5H), 7.10 (*J* = d, 5.5 Hz, 1H), 6.56 (dd, *J* = 6.4, 2.4 Hz, 1H), 6.29 (d, *J* = 2.4 Hz, 1H), 4.53 (ABdq, *J* = 15.2, 5.2 Hz, 1H), 4.44 (ABdq, *J* = 15.2, 6.4 Hz, 1H), 3.05 (s, 6H); ^{13}C NMR (150 MHz, $CDCl_3$) δ 181.5, 165.4, 161.3, 156.1, 154.7, 135.4, 130.4, 129.5, 129.0, 128.2, 128.0, 127.7, 127.2, 110.6, 109.1, 108.4, 97.6, 97.1, 49.2, 40.1; IR ν_{max} (KBr) 2927, 1699, 1465, 1441, 1349, 1326, 1116, 1088, 1068, 906, 694 cm^{-1}; HRMS (EI) m/z calcd for $C_{23}H_{19}BrN_2O_3S$ [M^+] 482.0300, found 482.0299.

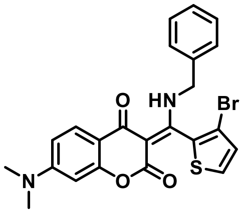

7g: R_f = 0.57 (40% EtOAc/hexanes); light brown solid; 214 mg; yield 91%; mp 200–202 °C; ^{1}H NMR (400 MHz, $CDCl_3$) δ 14.37 (b.s., 1H), 7.84 (d, *J* = 8.8 Hz, 1H), 7.43 (d, *J* = 1.2 Hz, 1H), 7.39–7.30 (m, 3H), 7.23 (dd, *J* = 8.4, 1.2 Hz, 2H), 6.92 (d, *J* = 1.6 Hz, 1H), 6.56 (dd, *J* = 8.8, 2.4 Hz, 1H), 6.30 (d, *J* = 2.4 Hz, 1H), 4.49 (d, *J* = 6.0 Hz, 2H), 3.06 (s, 6H); ^{13}C NMR (150 MHz, $CDCl_3$) δ 181.4, 166.2, 161.6, 156.1, 154.8, 135.9, 134.5, 129.4, 129.1, 128.2, 127.29, 127.28, 125.1, 110.1, 109.1, 108.4, 97.6, 97.1, 49.1, 40.1; IR ν_{max} (KBr) 2924, 2857, 1713, 1608, 1586, 1539,

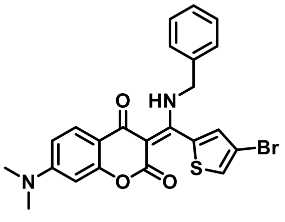

1456, 1408, 1384, 1336, 1238, 1158, 1118, 1056, 822 cm^{-1}; HRMS (EI) m/z calcd for $C_{23}H_{19}BrN_2O_3S$ [M^+] 482.0300, found 482.0301.

7h: R_f = 0.53 (40% EtOAc/hexanes); light brown solid; 196 mg; yield 83%; mp 186–188 °C; ^{1}H NMR (400 MHz, $CDCl_3$) δ 14.29 (b.s., 1H), 7.84 (d, *J* = 8.8 Hz, 1H), 7.39–7.29 (m, 3H), 7.23 (d, *J* = 7.6 Hz, 2H), 7.08 (d, *J* = 3.6 Hz, 1H), 6.77 (d, *J* = 3.6 Hz, 1H), 6.56 (dd, *J* = 8.8, 1.6 Hz, 1H), 6.30 (d, *J* = 1.6 Hz, 1H), 4.53 (d, *J* = 6.0 Hz, 2H), 3.06 (s, 6H); ^{13}C NMR (150 MHz, $CDCl_3$) δ 181.2, 161.3, 166.2, 155.9, 154.7, 136.0, 134.6, 130.1, 129.0, 128.1, 127.6, 127.3, 127.2, 115.4, 109.2, 108.4, 97.5, 97.1, 49.0, 40.1; IR ν_{max} (KBr) 2926, 1704, 1612, 1589, 1539, 1463, 1439, 1335, 1238, 1160, 1119, 1065, 964, 827, 744, 693 cm^{-1}; HRMS (EI) m/z calcd for $C_{23}H_{19}BrN_2O_3S$ [M^+] 482.0300, found 482.0294.

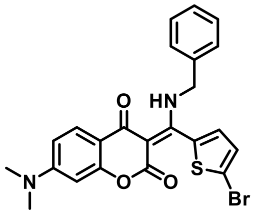

7i: R_f = 0.57 (40% EtOAc/hexanes); off–yellow solid; 184 mg; yield 86%; mp 210–212 °C; ^{1}H NMR (400 MHz, $CDCl_3$) δ 14.26 (b.s., 1H), 7.84 (d, *J* = 9.2 Hz, 1H), 7.39–7.29 (m, 3H), 7.23 (dd, *J* = 7.2, 1.2 Hz, 2H), 6.94 (d, *J* = 4.0 Hz, 1H), 6.79 (d, *J* = 4.0 Hz, 1H), 6.55 (dd, *J* = 9.2, 2.4 Hz, 1H), 6.30 (d, *J* = 2.8 Hz, 1H), 4.54 (d, *J* = 6.4 Hz, 2H), 3.06 (s, 6H); ^{13}C NMR (100 MHz, $CDCl_3$) δ 182.3, 166.1, 162.5, 156.0, 154.7, 136.0, 133.1, 131.7, 129.0, 128.1, 127.3, 127.2, 126.6, 126.4, 109.2, 108.4, 97.6, 97.0, 49.0, 40.1; IR ν_{max} (KBr) 1718, 1162, 1120, 1066, 994, 826, 803, 693 cm^{-1}; HRMS (EI) m/z calcd for $C_{23}H_{19}ClN_2O_3S$ [M^+] 438.0805, found 438.0804.

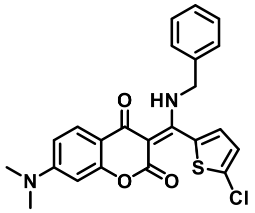

7j: R_f = 0.42 (40% EtOAc/hexanes); brown solid; 180 mg; yield 88%; mp 196–198 °C; ^{1}H NMR (400 MHz, $CDCl_3$) δ 14.03 (b.s., 1H), 7.85 (d, *J* = 8.8 Hz, 1H), 7.38–7.30 (m, 3H), 7.24 (d, *J* = 6.8 Hz, 2H), 6.84 (d, *J* = 4.8 Hz, 1H), 6.78 (dd, *J* = 4.8, 0.8 Hz, 1H), 6.55 (dd, *J* = 8.8, 2.4 Hz, 1H), 6.31 (d, *J* = 2.4 Hz, 1H), 4.55 (d, *J* = 6.0 Hz, 2H), 3.05 (s, 6H), 2.55 (d, *J* = 0.8 Hz, 3H); ^{13}C NMR (150 MHz, $CDCl_3$) δ 180.8, 168.0, 162.5, 155.9, 154.5, 143.2, 136.2, 130.5, 128.9, 128.0, 127.5, 127.24, 127.20, 125.6, 109.5, 108.3, 97.4, 97.2, 49.0, 40.1, 15.3; IR ν_{max} (KBr) 2922, 1715, 1610, 1538, 1431, 1228, 1119, 905, 838, 830, 698 cm^{-1}; HRMS (EI) m/z calcd for $C_{24}H_{22}N_2O_3S$ [M^+] 418.1351, found 418.1346.

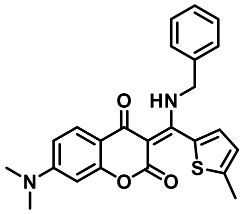

7k: R_f = 0.56 (40% EtOAc/hexanes); dark brown solid; 27 mg; yield 12%; mp 178–180 °C; ^{1}H NMR (400 MHz, $CDCl_3$) δ 13.86 (b.s., 1H), 7.84 (d, *J* = 8.8 Hz, 1H), 7.62 (d, *J* = 0.8 Hz, 1H), 7.40–7.31 (m, 3H), 7.24 (d, *J* = 6.4 Hz, 2H), 6.59 (d, *J* = 0.8 Hz, 1H), 6.56 (dd, *J* = 8.8, 2.4 Hz, 1H), 6.31 (d, *J* = 2.4 Hz, 1H), 4.55 (d, *J* = 6.0 Hz, 2H), 3.06 (s, 6H); ^{13}C NMR (150 MHz, $CDCl_3$) δ 181.1, 162.2, 161.3, 156.1, 154.8, 144.6, 142.4, 135.8, 129.1, 128.2, 127.24, 127.2, 115.1, 109.2, 108.5, 100.9, 97.3, 97.2, 49.6, 40.1; IR ν_{max} (KBr) 3132, 2916, 1705, 1616, 1586, 1435, 1119, 918, 822, 783 cm^{-1}; HRMS (EI) m/z calcd for $C_{23}H_{19}BrN_2O_4$ [M^+] 466.0528, found 466.0521.

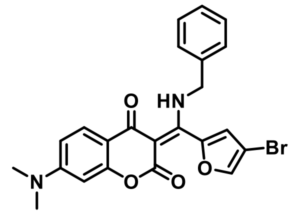

7l: R_f = 0.62 (40% EtOAc/hexanes); brown solid; 175 mg; yield 83%; mp 180–182 °C; ^{1}H NMR (400 MHz, $CDCl_3$) δ 14.08 (b.s., 1H), 7.86 (d, *J* = 9.2 Hz, 1H), 7.43 (d, *J* = 1.2 Hz, 1H), 6.96 (d, *J* = 1.2 Hz, 1H), 6.57 (dd, *J* = 9.2, 2.4 Hz, 1H), 6.30 (d, *J* = 2.4 Hz, 1H), 5.85 (ddt, *J* = 17.2, 10.4, 5.2 Hz, 1H), 5.29 (dd, *J* = 17.2, 1.6 Hz, 1H), 5.28 (dd, *J* = 10.4, 1.6 Hz, 1H), 3.92 (tt, *J* = 5.2, 1.6 Hz, 2H), 3.06 (s, 6H); ^{13}C NMR (100 MHz, $CDCl_3$) δ 181.2, 166.3, 156.0, 154.8, 134.4, 132.4, 129.3, 127.3, 125.0, 118.1, 110.0, 108.4, 97.5, 97.1, 47.4, 40.1; IR ν_{max} (KBr) 3092, 2924, 1694, 1575, 1349, 1115, 914, 819 cm^{-1}; HRMS (EI) m/z calcd for $C_{19}H_{17}BrN_2O_3S$ [M^+] 432.0143, found 432.0148.

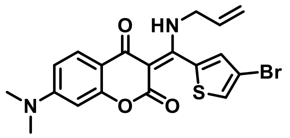

7m: R_f = 0.64 (40% EtOAc/hexanes); off–yellow solid; 177 mg; yield 81%; mp 144–148 °C; ^{1}H NMR (400 MHz, $CDCl_3$) δ 14.05 (b.s., 1H), 7.86 (d, *J* = 8.8 Hz, 1H), 7.43 (d, *J* = 1.6 Hz, 1H), 6.95 (d, *J* = 1.6 Hz, 1H), 6.56 (dd, *J* = 9.2, 2.4 Hz, 1H), 6.30 (d, *J* = 2.4 Hz, 1H), 3.30 (q, *J* = 6.8 Hz, 2H), 3.06 (s, 6H), 1.65 (quintet, *J* = 7.2 Hz, 2H), 1.41 (sextet, *J* = 7.2 Hz, 2H), 0.93 (t, *J* = 7.2 Hz, 3H); ^{13}C NMR (150 MHz, $CDCl_3$) δ 180.9, 166.0, 161.7, 156.0, 154.6, 134.7, 129.1, 127.1, 124.8, 110.0, 109.2, 108.4, 97.1, 97.0, 45.2, 40.1, 32.0, 20.0, 13.6; IR ν_{max} (KBr) 3090, 2960, 2936, 1699, 1615, 1575, 1548, 1346, 1229, 1111, 910, 777 cm^{-1}; HRMS (EI) m/z calcd for $C_{20}H_{21}BrN_2O_3S$ [M^+] 448.0456, found 448.0453.

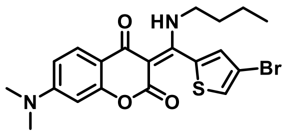

7n: R_f = 0.62 (40% EtOAc/hexanes); brown solid; 225 mg; yield 93%; mp 213–215 °C; 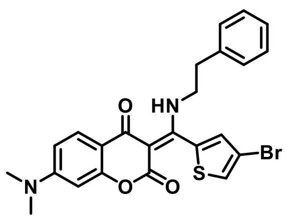

^{1}H NMR (400 MHz, $CDCl_3$) δ 14.17 (b.s., 1H), 7.87 (d, *J* = 8.8 Hz, 1H), 7.37 (d, *J* = 1.2 Hz, 1H), 7.34–7.24 (m, 3H), 7.11 (dd, *J* = 8.4, 1.6 Hz, 2H), 6.57 (dd, *J* = 9.2, 1.6 Hz, 1H), 6.47 (d, *J* = 1.6 Hz, 1H), 6.29 (d, *J* = 2.4 Hz, 1H), 3.52 (q, *J* = 7.2 Hz, 2H), 3.06 (s, 6H), 2.93 (t, *J* = 6.8 Hz, 2H); ^{13}C NMR (100 MHz, $CDCl_3$) δ 181.4, 166.3, 161.6, 156.0, 154.7, 137.1, 134.3, 129.2, 128.8, 127.2, 127.0, 124.7, 109.9, 109.2, 108.4, 97.1, 97.08, 46.9, 40.1, 36.7, 30.9; IR ν_{max} (KBr) 2926, 1707, 1567, 1453, 1408, 1385, 1350, 1238, 1160, 1117, 1073, 784, 721, 695 cm^{-1}; HRMS (EI) m/z calcd for $C_{24}H_{21}BrN_2O_3S$ [M^+] 496.0456, found 496.0452.

7o: R_f = 0.59 (40% EtOAc/hexanes); off–brown solid; 208 mg; yield 80%; mp 202–204 °C; 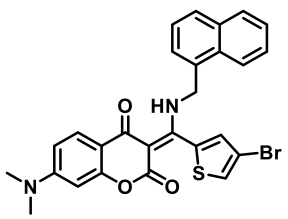

^{1}H NMR (400 MHz, $CDCl_3$) δ 14.48 (b.s., 1H), 7.91–7.80 (m, 4H), 7.60–7.48 (m, 2H), 7.46–7.37 (m, 3H), 7.00 (d, *J* = 0.8 Hz, 1H), 6.53 (dd, *J* = 9.2, 2.4 Hz, 1H), 6.29 (d, *J* = 2.0 Hz, 1H), 4.93 (d, *J* = 5.6 Hz, 2H), 3.04 (s, 6H); ^{13}C NMR (150 MHz, $CDCl_3$) δ 181.6, 166.2, 161.9, 156.0, 154.7, 134.4, 133.8, 131.4, 130.6, 129.5, 129.1, 129.0, 127.3, 126.9, 126.2, 125.8, 125.4, 125.1, 122.3, 110.2, 109.1, 108.4, 97.7, 97.1, 46.9, 40.1; IR ν_{max} (KBr) 2924, 1714, 1568, 1544, 1526, 1460, 1347, 1316, 1157, 1116, 903, 869, 823 cm^{-1}; HRMS (EI) m/z calcd for $C_{27}H_{21}BrN_2O_3S$ [M^+] 532.0456, found 532.0460.

13: R_f = 0.50 (5% MeOH/DCM); yellow solid; 255 mg; yield 67%; mp 192–194 °C; 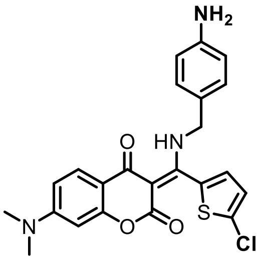

^{1}H NMR (400 MHz, $CDCl_3$) δ 14.10 (b.s., 1H), 7.83 (d, *J* = 8.2 Hz, 1H), 7.09 (d, *J* = 3.6 Hz, 1H), 7.00 (d, *J* = 8.4 Hz, 2H), 6.79 (d, *J* = 4.0 Hz, 1H), 6.65 (d, *J* = 8.4 Hz, 2H), 6.54 (dd, *J* = 9.2, 2.4 Hz, 1H), 6.29 (d, *J* = 2.4 Hz, 1H), 4.40 (d, *J* = 6.0 Hz, 2H), 3.71 (b.s., 2H), 3.05 (s, 6H); ^{13}C NMR (150 MHz, $CDCl_3$) δ 183.8, 172.2, 165.7, 156.0, 154.7, 146.4, 134.9, 130.2, 128.7, 127.6, 127.3, 125.5, 115.5, 115.3, 109.4. 108.4, 97.3, 97.1, 48.9, 40.2; IR ν_{max} (KBr) cm^{-1}; 2813, 1812, 1598. 1532, 1472, 1364, 1247, 1108, 947, 834 HRMS (EI) m/z calcd for $C_{23}H_{20}ClN_3O_3S$ [M^+] 453.0914, found 453.0911

13: R_f = 0.50 (5% MeOH/DCM); brown solid; 157 mg; yield 71%; mp 174–176 °C; ^{1}H NMR (400 MHz, $CDCl_3$) δ 14.97 (b.s., 1H), 14.29 (b.s., 1H), 8.07 (d, *J* = 7.6 Hz, 1H), 7.83 (d, *J* = 8.8 Hz, 1H), 7.56 (td, *J* = 8.4, 1.6 Hz, 1H), 7.22 (d, *J* = 8.4 Hz, 2H), 7.11 (d, *J* = 8.8 Hz, 2H), 7.05 (d, *J* = 7.6 Hz, 2H), 7.04 (d, *J* = 3.2 Hz, 1H), 6.87 (d, *J* = 8.4 Hz, 2H), 6.67 (d, *J* = 3.6 Hz, 1H), 6.59 (d, *J* = 9.2 Hz, 2H), 6.57 (dd, *J* = 9.2, 2.4 Hz, 1H), 6.30 (d, *J* = 2.4 Hz, 1H), 4.46 (d, *J* = 6.0 Hz, 2H), 3.07 (b.s., 6H), 3.00 (s, 6H); ^{13}C NMR (150 MHz, $CDCl_3$) δ 172.8, 167.2, 166.5, 156.0, 154.9, 154.1, 151.8, 137.9, 134.5, 134.4, 134.1, 133.8, 130.5, 130.2, 129.5, 128.3, 127.8, 127.7, 127.3, 126.1, 125.5, 123.6, 120.8, 118.5, 116.8, 115.5, 111.2, 111.0, 108.5, 97.8, 97.7, 97.1, 48.3, 40.2, 40.0; IR ν_{max} (KBr) 2924, 2936, 1750, 1620, 1578, 1533, 1400, 1365, 1130, 804, 800 cm^{-1}; HRMS (EI) m/z calcd for $C_{41}H_{33}ClN_4O_6S$ [M^+] 744.1809, found 744.1805

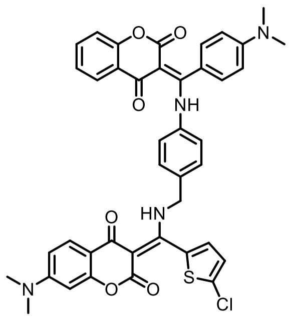

2.1 X-ray crystallographic analysis of compound 7b

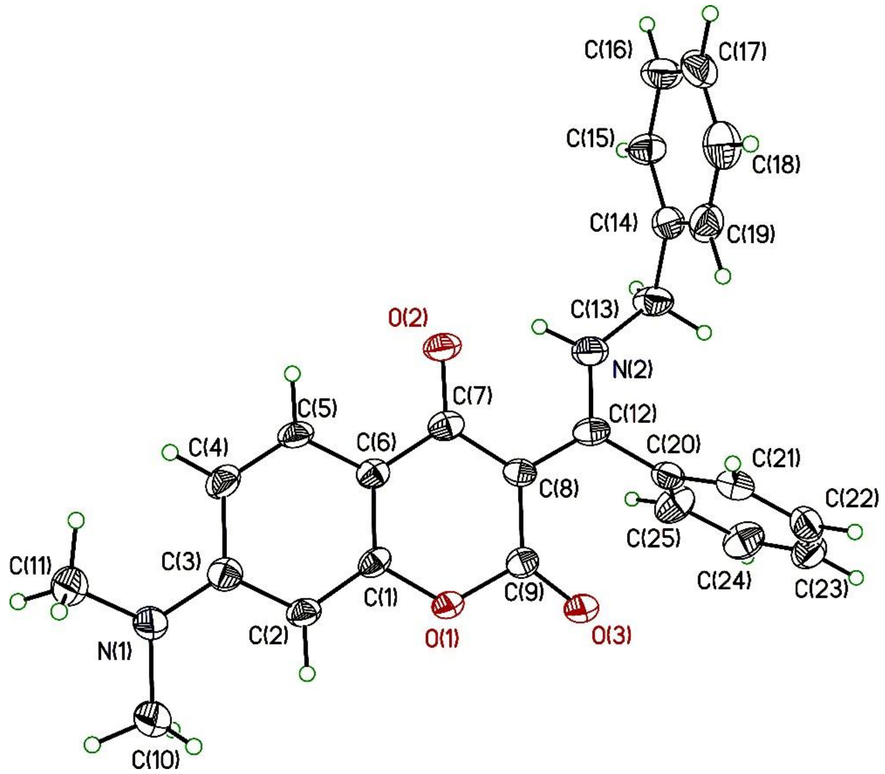

Figure S1: ORTEP diagram of compound **7b**. The ellipsoid contour probability levels: 50%

Table S1. Crystal data and structure refinement for **7b**.

Identification code	bmk2320	
Empirical formula	$C_{25} H_{22} N_2 O_3$	
Formula weight	398.45	
Temperature	150(2) K	
Wavelength	0.71073 Å	
Crystal system	Orthorhombic	
Space group	P 21 21 21	
Unit cell dimensions	a = 8.1692(3) Å	a= 90°.
	b = 12.7582(5) Å	b= 90°.
	c = 19.2379(8) Å	g = 90°.
Volume	2005.06(14) Å^3	
Z	4	
Density (calculated)	1.320 Mg/m^3	
Absorption coefficient	0.087 mm^{-1}	
F(000)	840	
Crystal size	0.44 x 0.40 x 0.17 mm^3	
Theta range for data collection	3.14 to 26.38°.	
Index ranges	-9<=h<=10, -15<=k<=15, -24<=l<=24	
Reflections collected	25009	
Independent reflections	4081 [R(int) = 0.0572]	
Completeness to theta = 26.38°	99.8 %	
Absorption correction	Semi-empirical from equivalents	
Max. and min. transmission	0.9853 and 0.9626	
Refinement method	Full-matrix least-squares on F^2	
Data / restraints / parameters	4081 / 0 / 278	
Goodness-of-fit on F^2	1.021	
Final R indices [I>2sigma(I)]	R1 = 0.0420, wR2 = 0.0940	
R indices (all data)	R1 = 0.0614, wR2 = 0.1034	
Absolute structure parameter	0.5(13)	
Largest diff. peak and hole	0.266 and -0.211 e.Å^{-3}	

2.2 X-ray crystallographic analysis of compound 7g

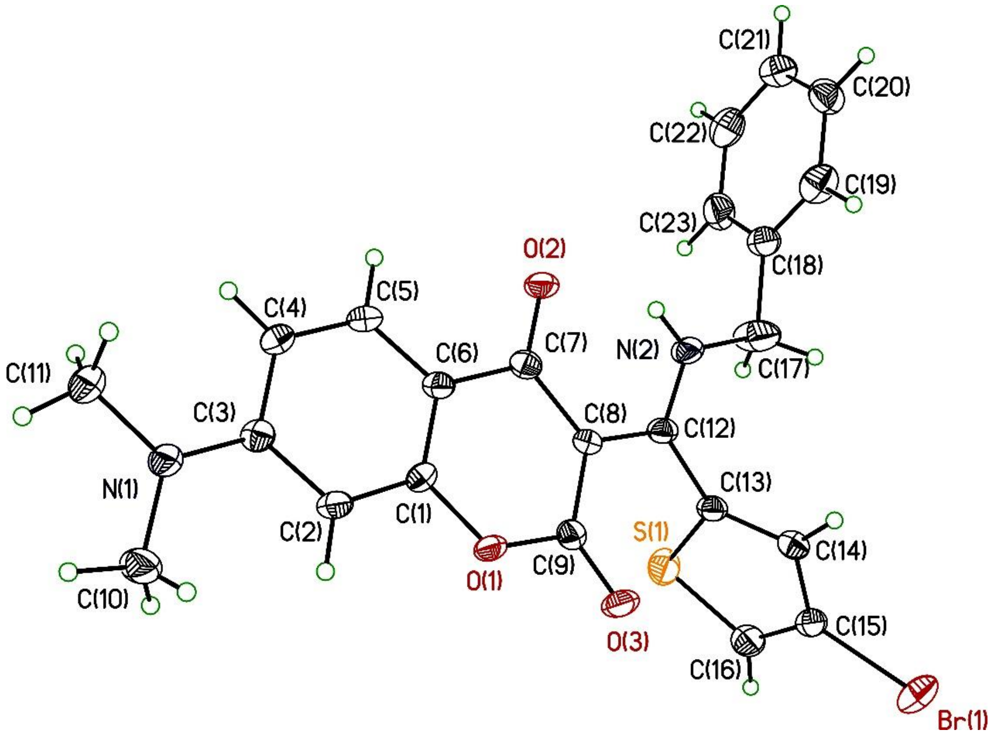

Figure S2: ORTEP diagram of compound **7g**. The ellipsoid contour probability levels: 50%

Table S2. Crystal data and structure refinement for **7g**.

Identification code	fc036	
Empirical formula	$C_{23} H_{19} Br N_2 O_3 S$	
Formula weight	483.37	
Temperature	150(2) K	
Wavelength	0.71073 Å	
Crystal system	Triclinic	
Space group	P-1	
Unit cell dimensions	a = 7.8372(3) Å	a= 101.177(3)°.
	b = 13.4564(5) Å	b= 100.437(3)°.
	c = 20.9843(8) Å	g = 99.881(3)°.
Volume	2085.54(14) $Å^3$	
Z	4	
Density (calculated)	1.539 Mg/m^3	
Absorption coefficient	2.099 mm^{-1}	
F(000)	984	
Crystal size	0.46 x 0.42 x 0.23 mm^3	
Theta range for data collection	2.826 to 29.146°.	
Index ranges	-10<=h<=9, -16<=k<=17, -22<=l<=28	
Reflections collected	16873	
Independent reflections	9548 [R(int) = 0.0312]	
Completeness to theta = 25.242°	99.9 %	
Absorption correction	Semi-empirical from equivalents	
Max. and min. transmission	1.00000 and 0.90110	
Refinement method	Full-matrix least-squares on F^2	
Data / restraints / parameters	9548 / 0 / 549	
Goodness-of-fit on F^2	1.009	
Final R indices [I>2sigma(I)]	R1 = 0.0509, wR2 = 0.1031	
R indices (all data)	R1 = 0.0809, wR2 = 0.1167	
Extinction coefficient	n/a	
Largest diff. peak and hole	0.848 and -0.477 e.$Å^{-3}$	

2.3 X-ray crystallographic analysis of compound 7i

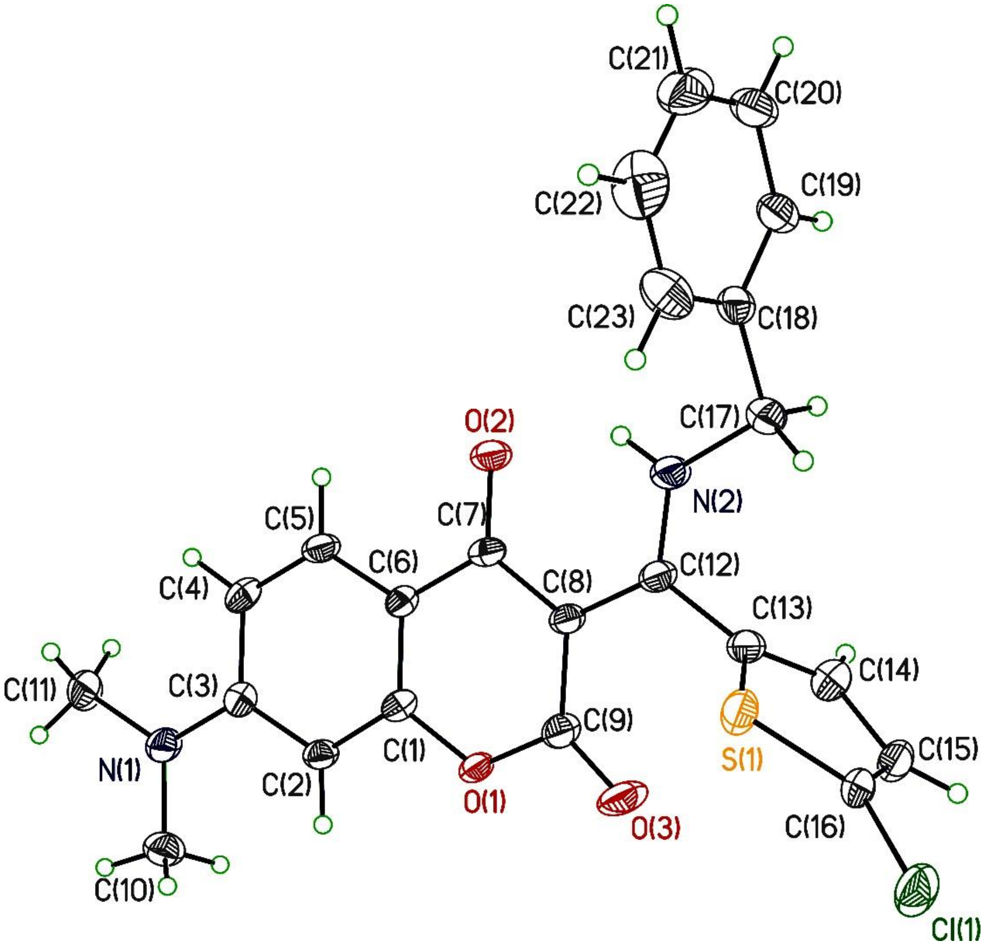

Figure S3: ORTEP diagram of compound **7i**. The ellipsoid contour probability levels: 50%

Table S3. Crystal data and structure refinement for **7i**.

Identification code	fc067	
Empirical formula	$C_{20} H_{21} Br N_2 O_3 S$	
Formula weight	449.36	
Temperature	150(2) K	
Wavelength	0.71073 Å	
Crystal system	Monoclinic	
Space group	$P2_1/c$	
Unit cell dimensions	a = 9.863(5) Å	a= 90°.
	b = 12.012(3) Å	b= 96.40(4)°.
	c = 33.719(8) Å	g = 90°.
Volume	3970(3) $Å^3$	
Z	8	
Density (calculated)	1.504 Mg/m^3	
Absorption coefficient	2.199 mm^{-1}	
F(000)	1840	
Crystal size	0.47 x 0.43 x 0.10 mm^3	
Theta range for data collection	2.848 to 29.200°.	
Index ranges	-13<=h<=12, -14<=k<=15, -45<=l<=44	
Reflections collected	16696	
Independent reflections	8844 [R(int) = 0.1179]	
Completeness to theta = 25.242°	98.1 %	
Absorption correction	Semi-empirical from equivalents	
Max. and min. transmission	1.00000 and 0.50372	
Refinement method	Full-matrix least-squares on F^2	
Data / restraints / parameters	8844 / 0 / 491	
Goodness-of-fit on F^2	1.053	
Final R indices [I>2sigma(I)]	R1 = 0.1011, wR2 = 0.2524	
R indices (all data)	R1 = 0.1819, wR2 = 0.3527	
Extinction coefficient	0.0035(8)	
Largest diff. peak and hole	1.225 and -1.376 e.$Å^{-3}$	

2.4 X-ray crystallographic analysis of compound 7m

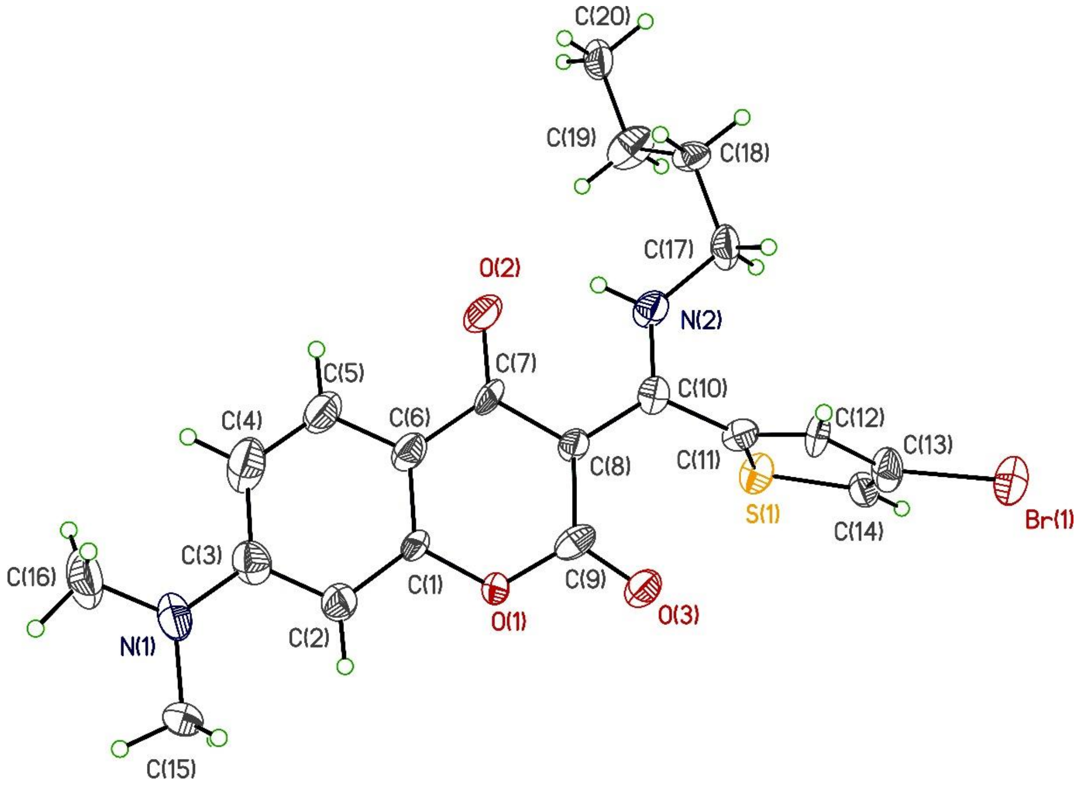

Figure S4: ORTEP diagram of compound **7m**. The ellipsoid contour probability levels: 50%

Table S4. Crystal data and structure refinement for **7m**.

Identification code	fc112	
Empirical formula	$C_{23} H_{19} Cl N_2 O_3 S$	
Formula weight	438.91	
Temperature	150(2) K	
Wavelength	0.71073 Å	
Crystal system	Monoclinic	
Space group	$P2_1/c$	
Unit cell dimensions	a = 18.9673(10) Å	a= 90°.
	b = 13.5198(7) Å	b= 102.855(5)°.
	c = 16.4123(8) Å	g = 90°.
Volume	4103.2(4) $Å^3$	
Z	8	
Density (calculated)	1.421 Mg/m^3	
Absorption coefficient	0.316 mm^{-1}	
F(000)	1824	
Crystal size	0.50 x 0.40 x 0.30 mm^3	
Theta range for data collection	2.953 to 29.306°.	
Index ranges	-26<=h<=24, -11<=k<=18, -21<=l<=12	
Reflections collected	19594	
Independent reflections	9622 [R(int) = 0.0415]	
Completeness to theta = 25.242°	99.9 %	
Absorption correction	Semi-empirical from equivalents	
Max. and min. transmission	1.00000 and 0.99299	
Refinement method	Full-matrix least-squares on F^2	
Data / restraints / parameters	9622 / 0 / 549	
Goodness-of-fit on F^2	1.028	
Final R indices [I>2sigma(I)]	R1 = 0.0566, wR2 = 0.1229	
R indices (all data)	R1 = 0.1010, wR2 = 0.1476	
Extinction coefficient	n/a	
Largest diff. peak and hole	0.388 and -0.315 e.$Å^{-3}$	

3. Crystalline state reversible color change of the compound 7i

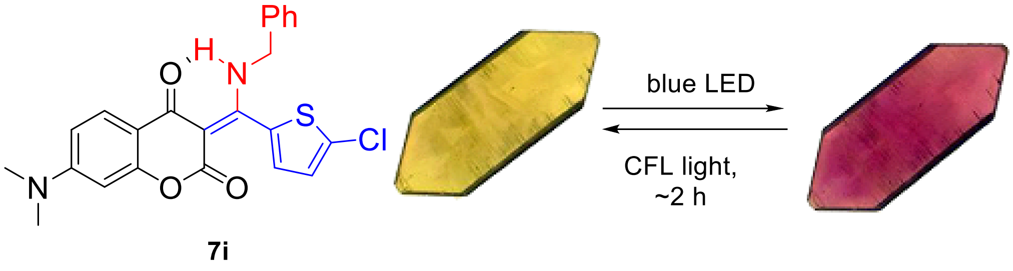

Figure S5: Reversible color change in compound **7i** when irradiated with blue LED (15 sec) and CFL light.

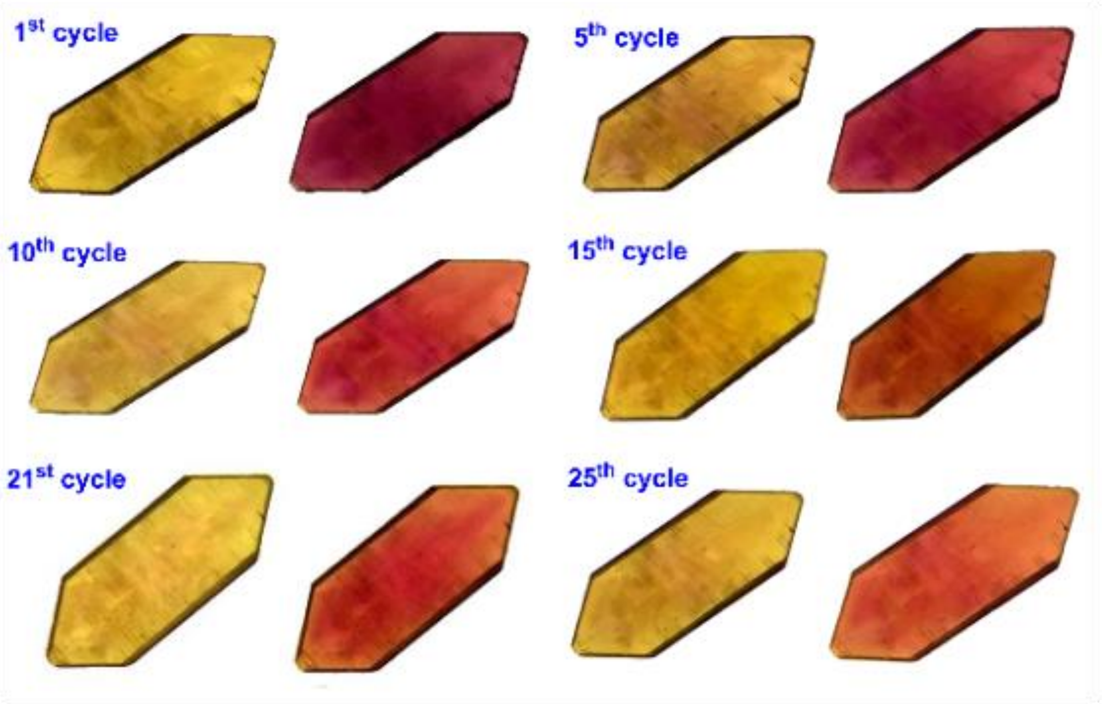

Figure S6: Reversibility of **7i**. A significant photo-bleaching can be seen at the 25th cycle.

4. UV-absorbance profile of the prepared compounds

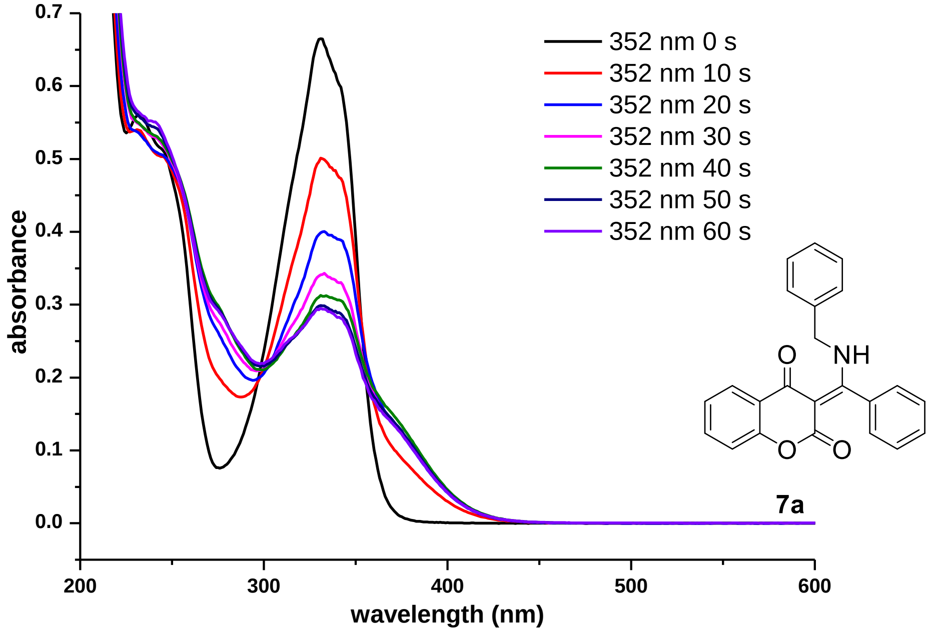

Figure S7: UV-absorption profile of **7a** (3.0×10^{-5} M in CH_3CN) during the UV irradiation (352 nm).

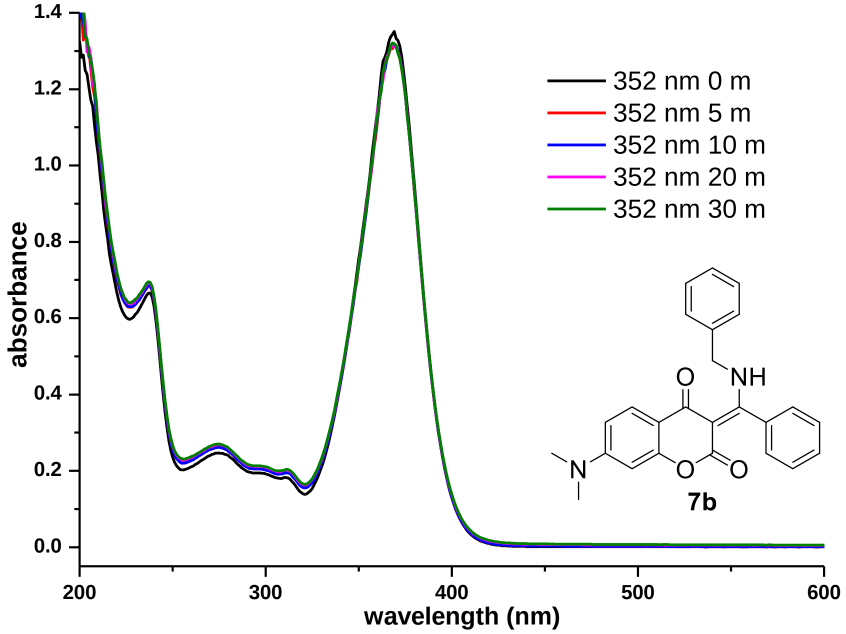

Figure S8: UV-absorption profile of **7b** (3.0×10^{-5} M in CH_3CN) during the UV irradiation (352 nm).

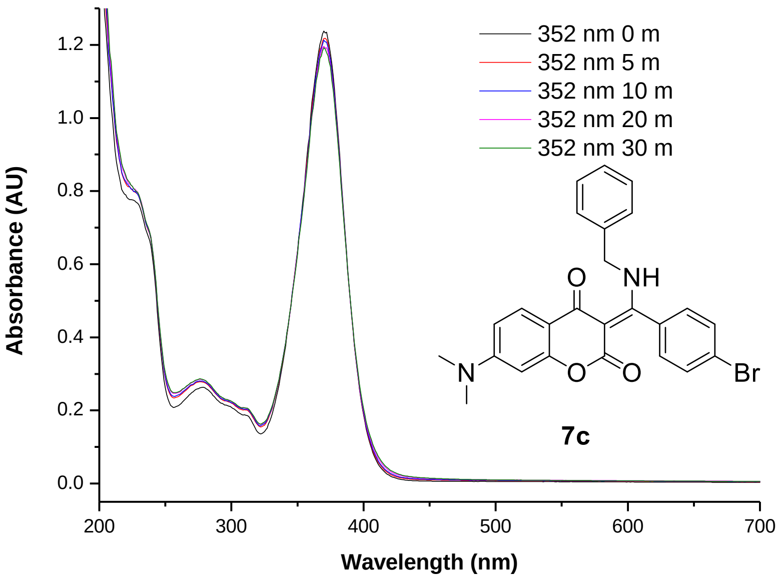

Figure S9: UV-absorption profile of **7c** (3.0×10^{-5} M in CH_3CN) during the UV irradiation (352 nm).

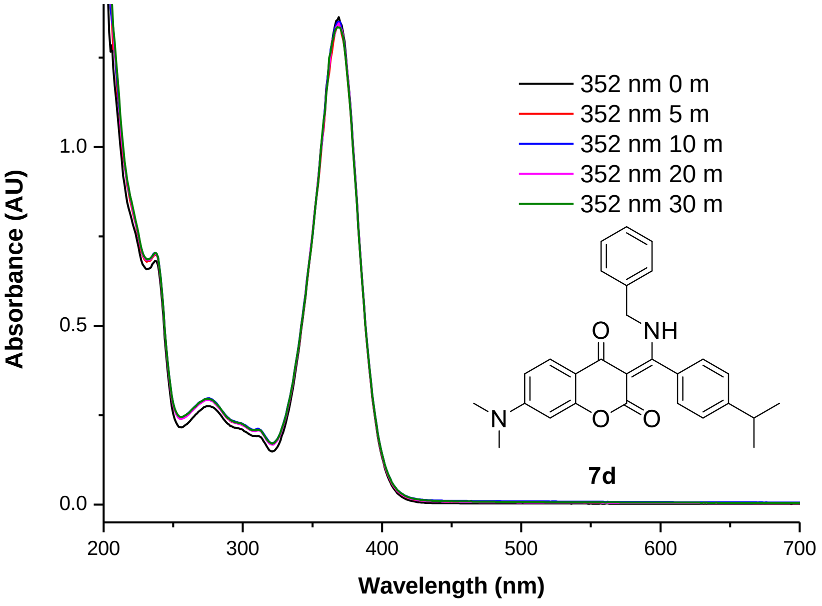

Figure S10: UV-absorption profile of **7d** (3.0×10^{-5} M in CH_3CN) during the UV irradiation (352 nm).

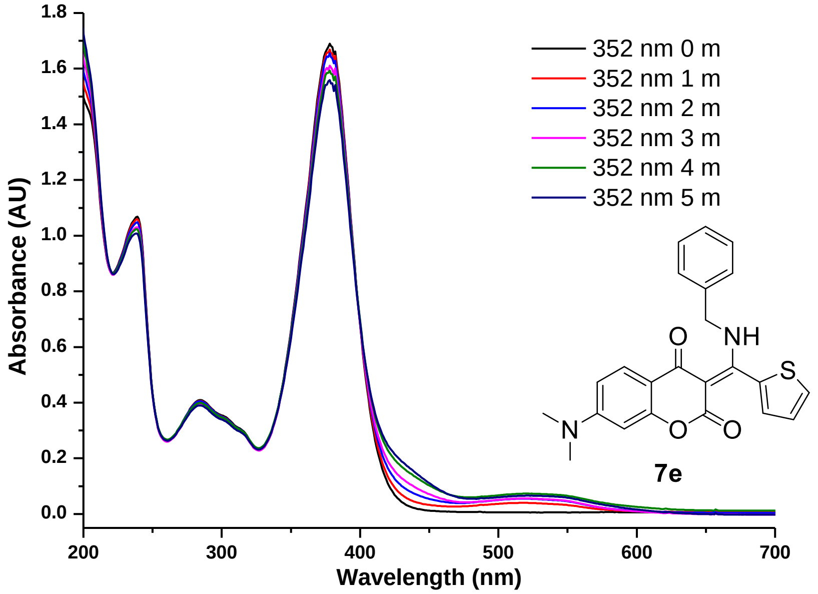

Figure S11: UV-absorption profile of **7e** (3.0×10^{-5} M in CH_3CN) during the UV irradiation (352 nm).

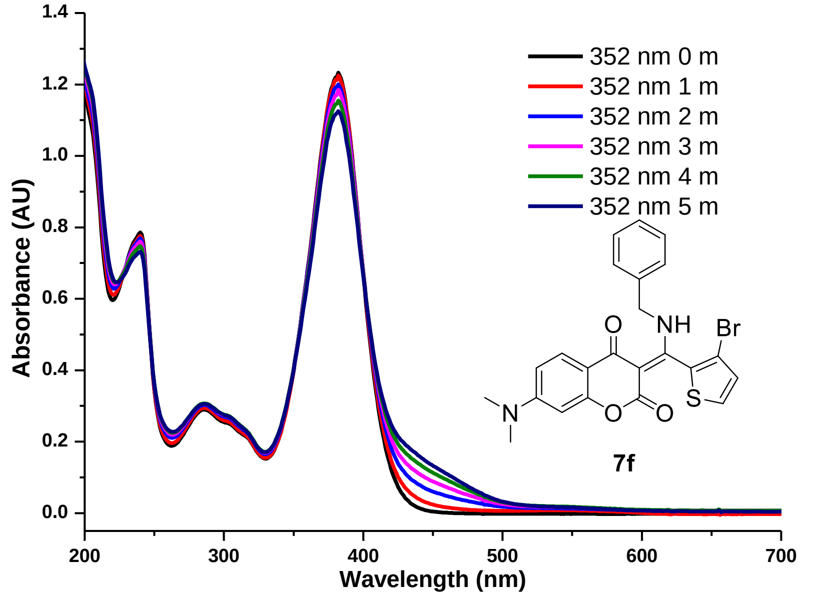

Figure S12: UV-absorption profile of **7f** (3.0×10^{-5} M in CH_3CN) during the UV irradiation (352 nm).

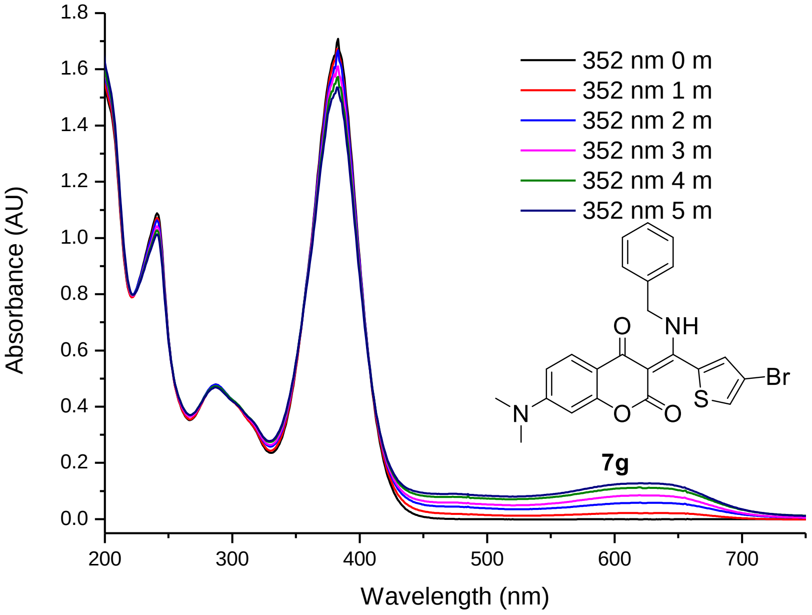

Figure S13: UV-absorption profile of **7g** (3.0×10^{-5} M in CH_3CN) during the UV irradiation (352 nm).

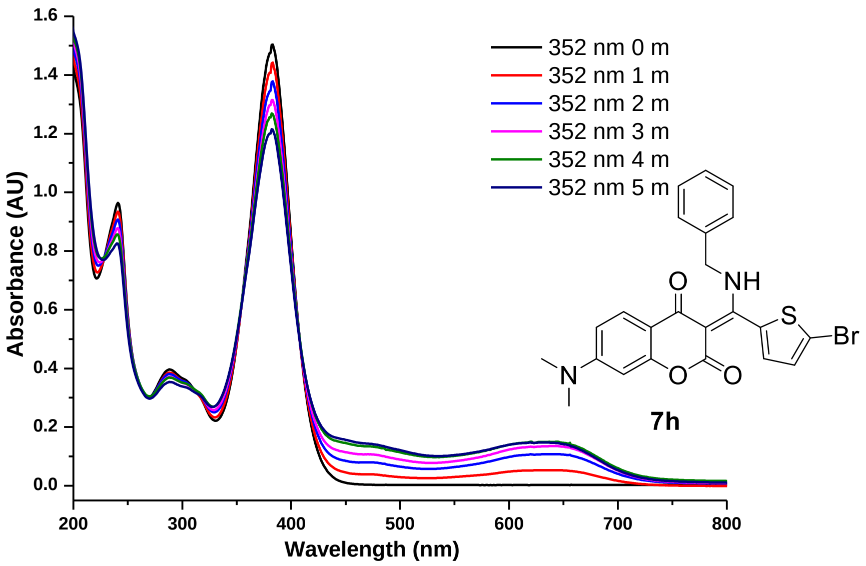

Figure S14: UV-absorption profile of **7h** (3.0×10^{-5} M in CH_3CN) during the UV irradiation (352 nm).

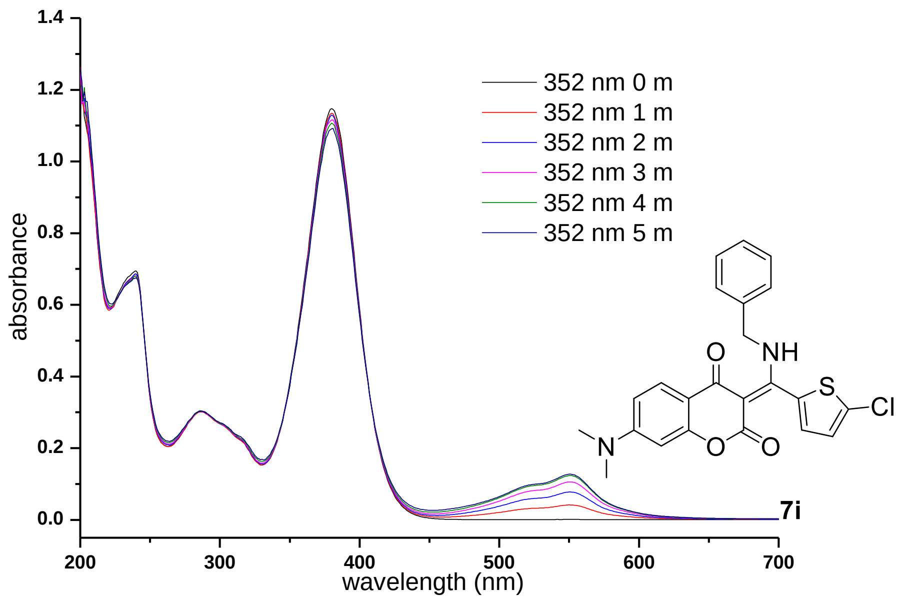

Figure S15: UV-absorption profile of **7i** (3.0×10^{-5} M in CH_3CN) during the UV irradiation (352 nm).

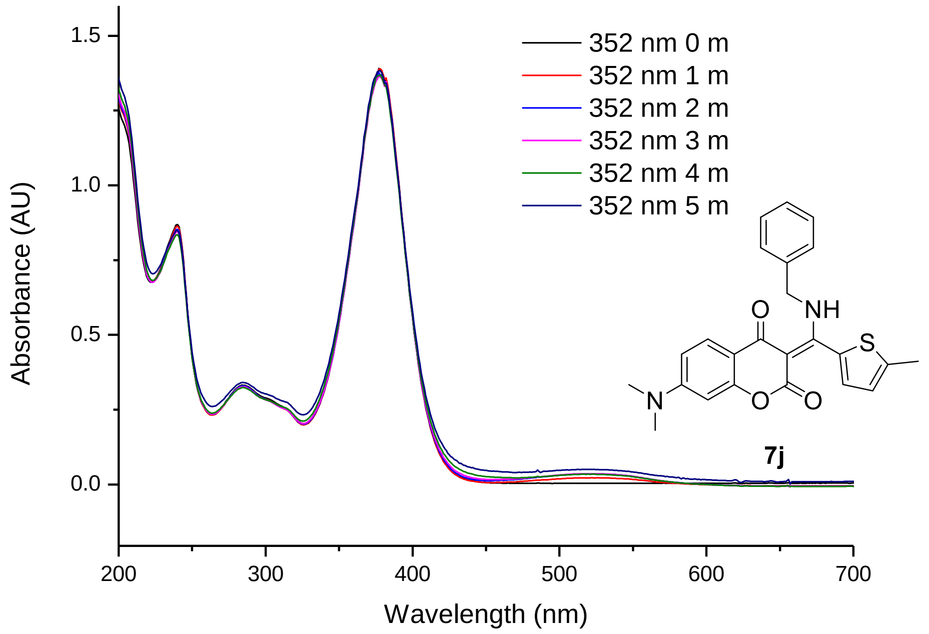

Figure S16: UV-absorption profile of **7j** (3.0×10^{-5} M in CH_3CN) during the UV irradiation (352 nm).

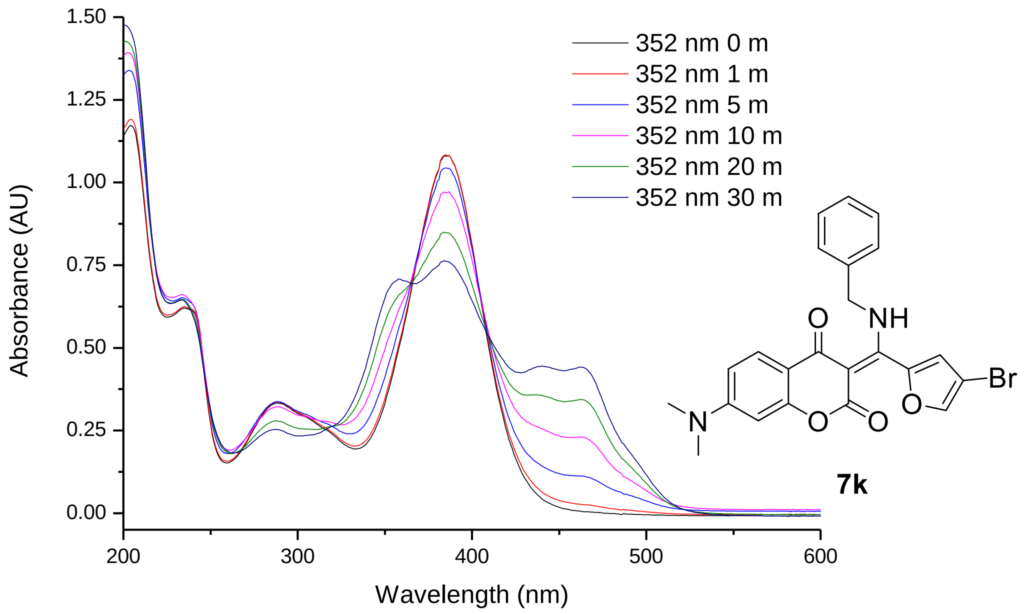

Figure S17: UV-absorption profile of **7k** (3.0×10^{-5} M in CH_3CN) during the UV irradiation (352 nm).

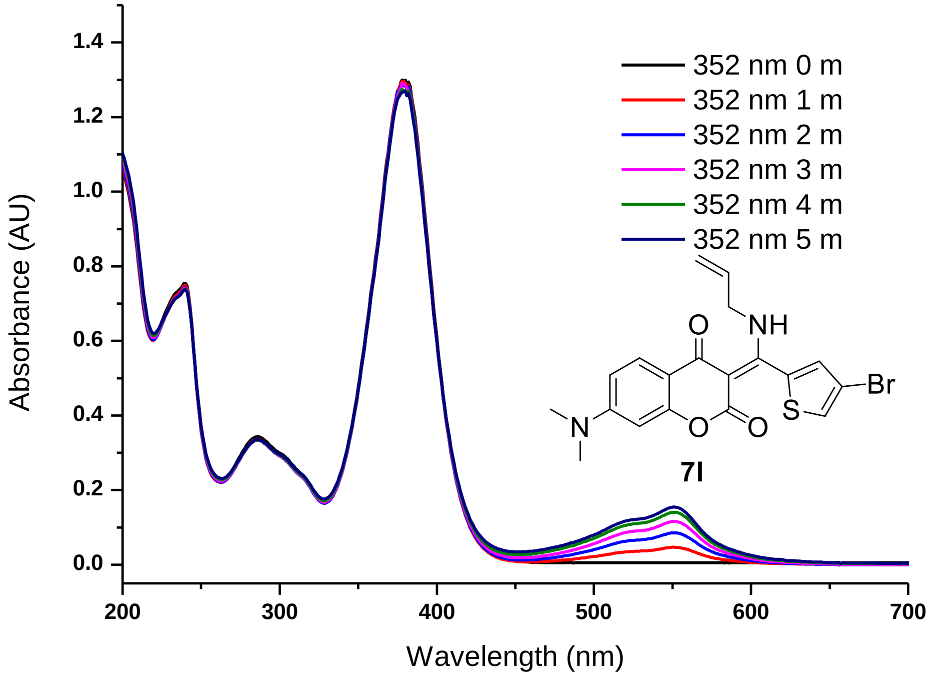

Figure S18: UV-absorption profile of **7l** (3.0×10^{-5} M in CH_3CN) during the UV irradiation (352 nm).

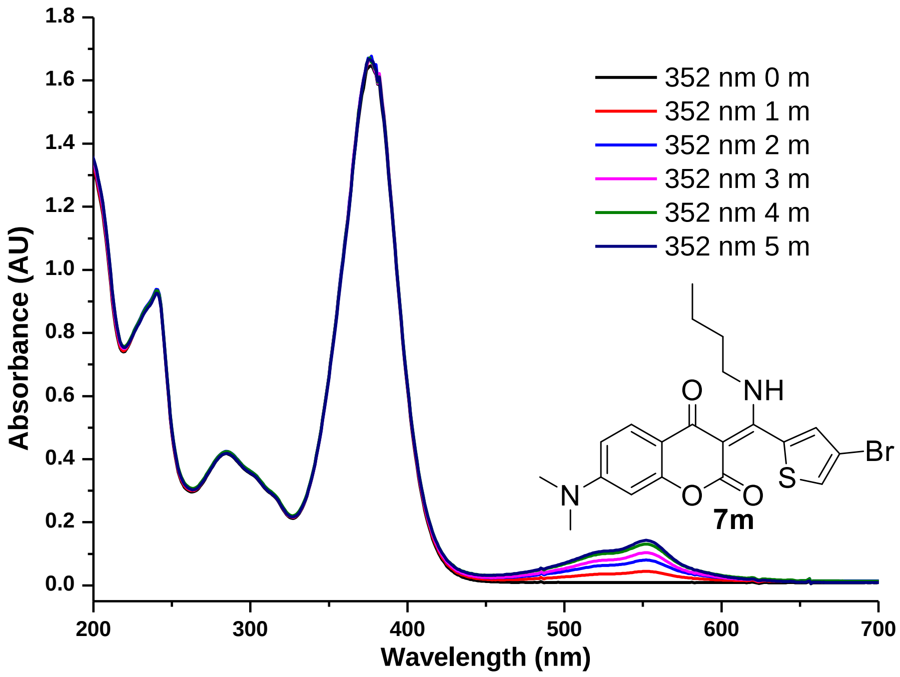

Figure S19: UV-absorption profile of **7m** (3.0×10^{-5} M in CH_3CN) during the UV irradiation (352 nm).

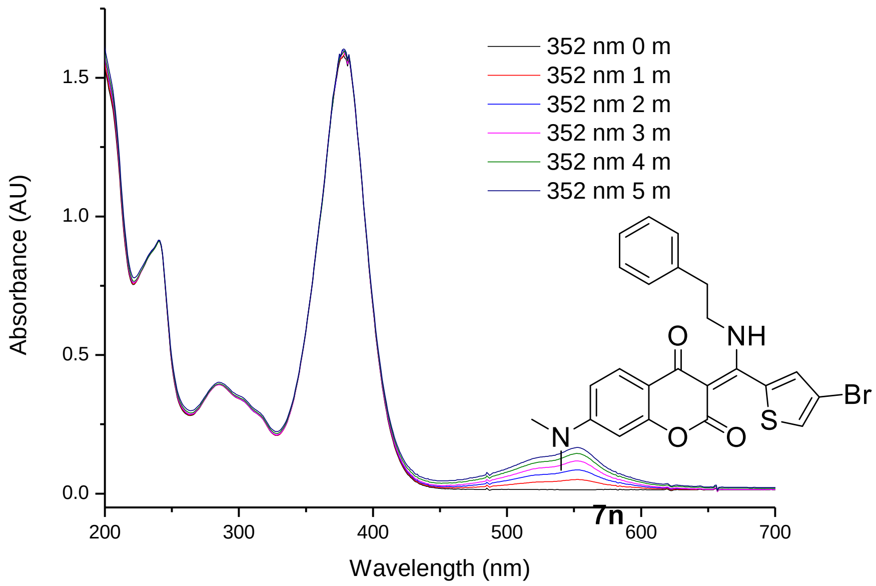

Figure S20: UV-absorption profile of **7n** (3.0×10^{-5} M in CH_3CN) during the UV irradiation (352 nm).

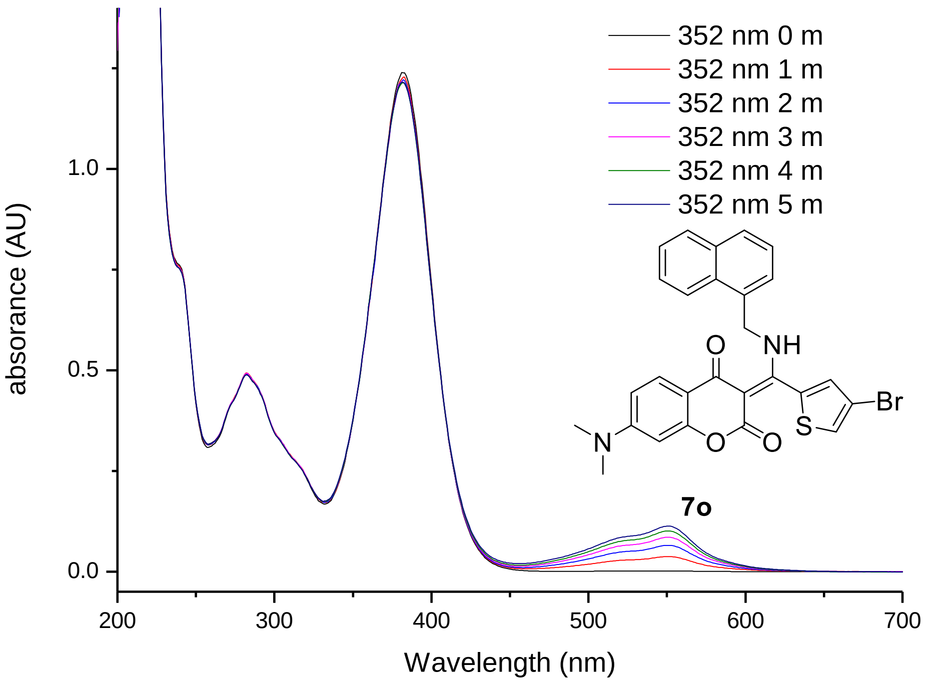

Figure S21: UV-absorption profile of **7o** (3.0×10^{-5} M in CH_3CN) during the UV irradiation (352 nm).

^{1}H and ^{13}C NMR spectra of the prepared compounds

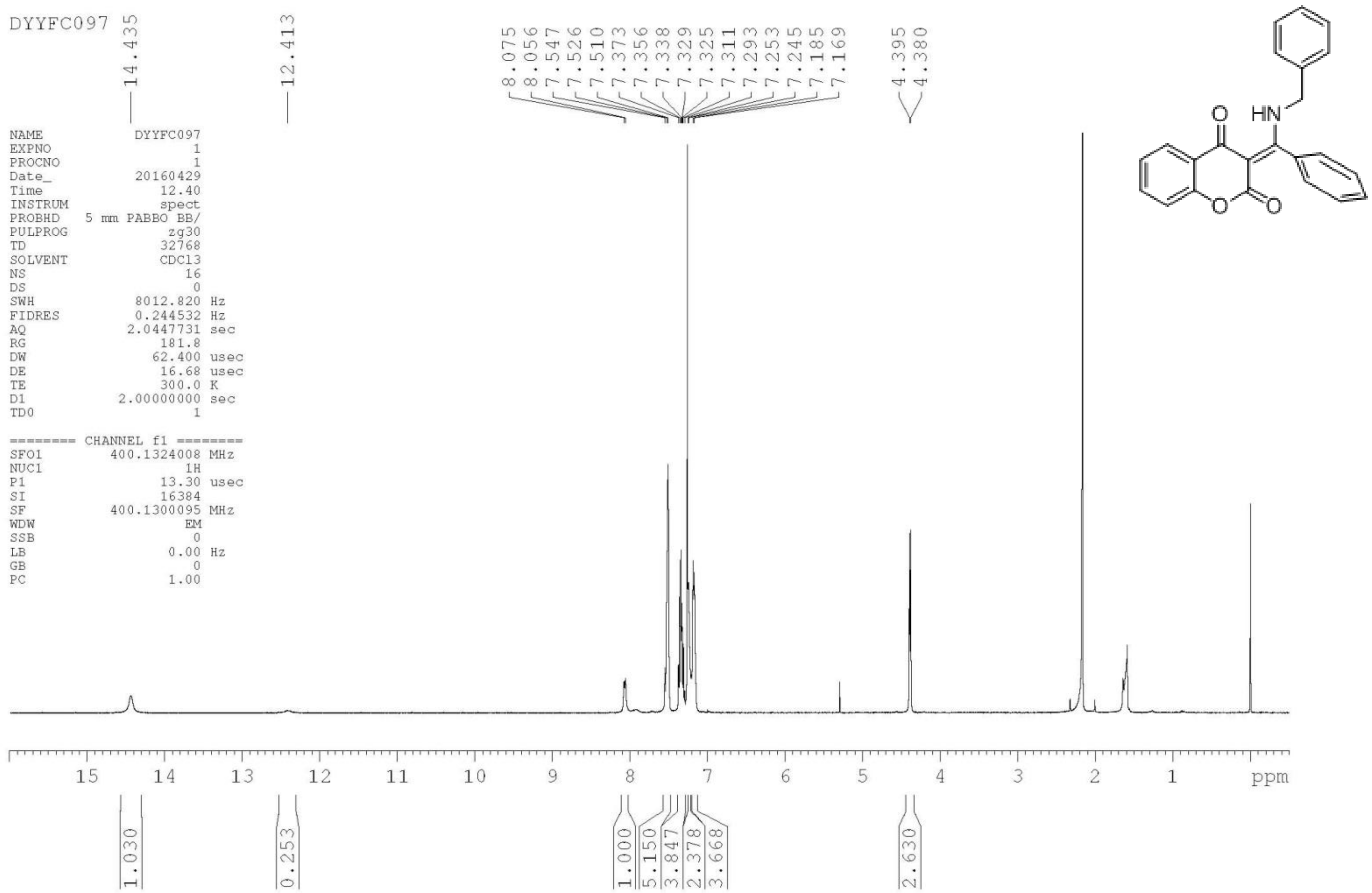
DYYFC097
14.435
12.413
8.075
8.056
7.547
7.526
7.510
7.373
7.356
7.338
7.329
7.325
7.311
7.293
7.253
7.245
7.185
7.169
4.395
4.380
HN
O
NAME DYYFC097
EXPNO 1
PROCNO 1
Date_ 20160429
Time 12.40
INSTRUM spect
PROBHD 5 mm PABBO BB/
PULPROG zg30
TD 32768
SOLVENT CDCl3
NS 16
DS 0
SWH 8012.820 Hz
FIDRES 0.244532 Hz
AQ 2.0447731 sec
RG 181.8
DW 62.400 usec
DE 16.68 usec
TE 300.0 K
D1 2.00000000 sec
TD0 1
======== CHANNEL f1 ========
SFO1 400.1324008 MHz
NUC1 1H
P1 13.30 usec
SI 16384
SF 400.1300095 MHz
WDW EM
SSB 0
LB 0.00 Hz
GB 0
PC 1.00
15 14 13 12 11 10 9 8 7 6 5 4 3 2 1 ppm
1.030
0.253
1.000
5.150
3.847
2.378
3.668
2.630

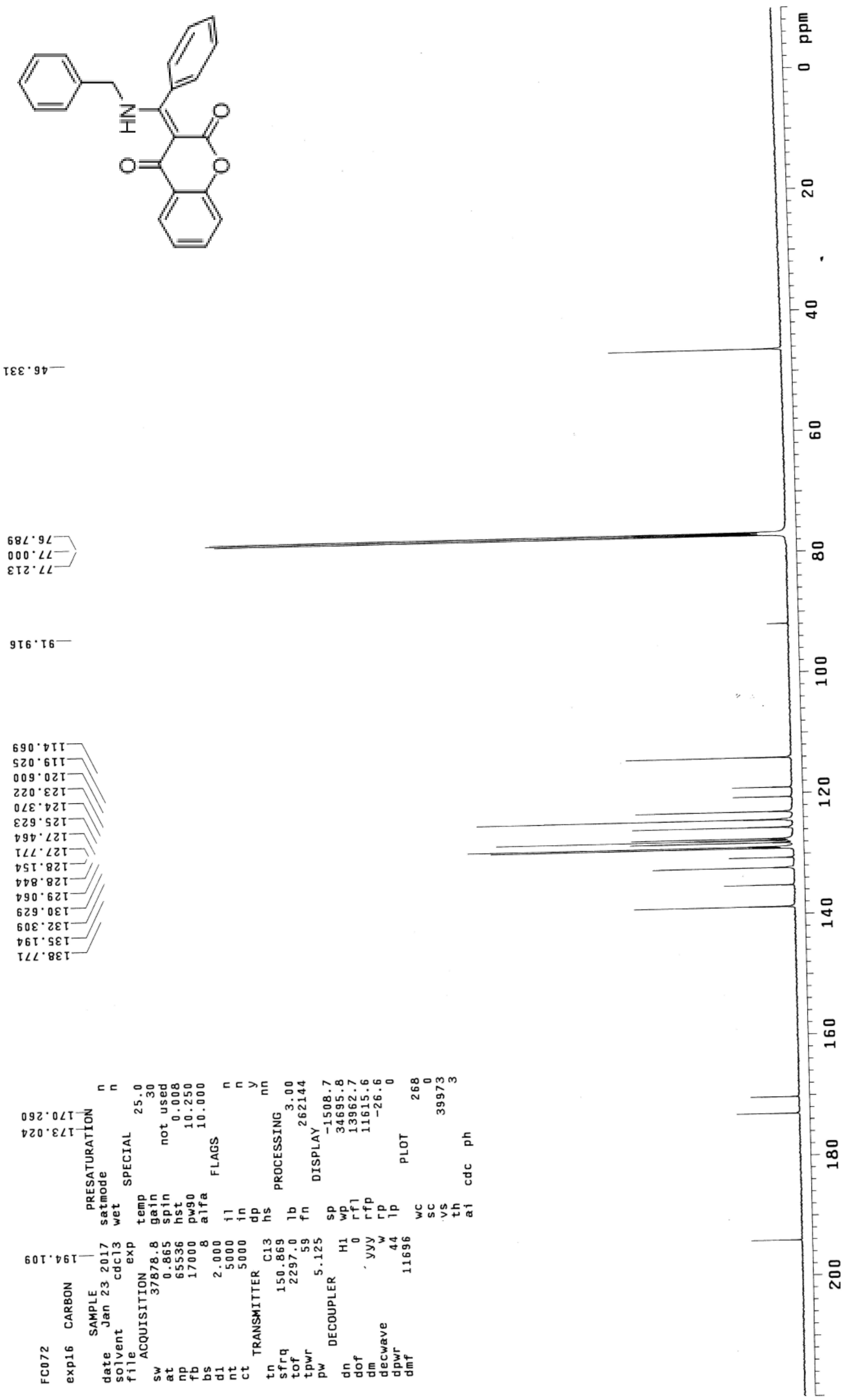

FC072
exp16 CARBON
46.331
76.789
77.000
77.213
91.916
114.069
119.025
120.600
123.022
124.370
125.623
127.464
127.771
128.154
128.844
129.064
130.629
132.309
135.194
138.771
170.260
173.024
194.109
ppm
0
20
40
60
80
100
120
140
160
180
200

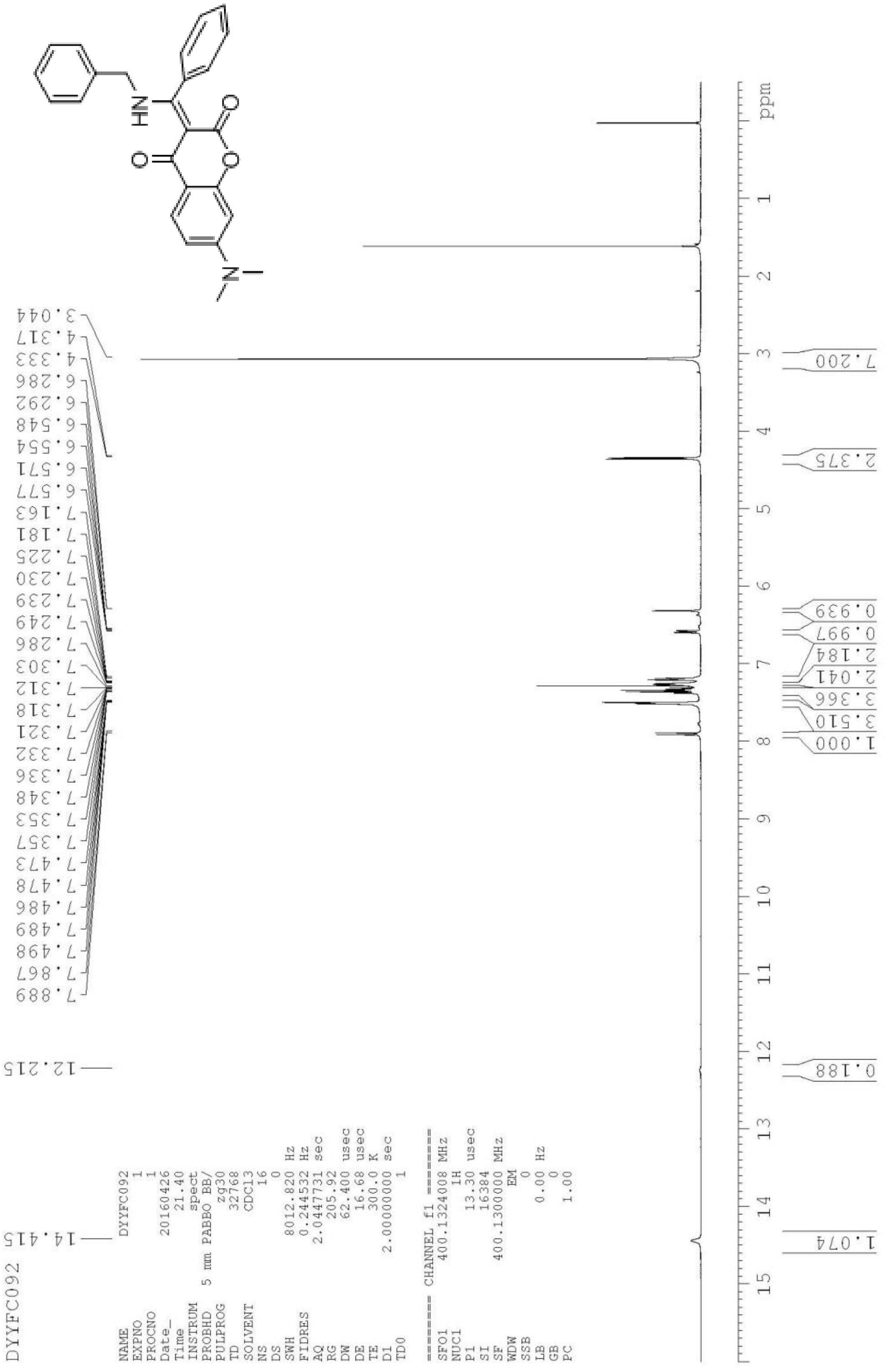
DYYFC092
HN
O
O
O
N
14.415
12.215
3.044
4.317
4.333
6.286
6.292
6.548
6.554
6.571
6.577
7.163
7.181
7.225
7.230
7.239
7.249
7.286
7.303
7.312
7.318
7.321
7.332
7.336
7.348
7.353
7.357
7.473
7.478
7.486
7.489
7.498
7.867
7.889
NAME DYYFC092
EXPNO 1
PROCNO 1
Date_ 20160426
Time 21.40
INSTRUM spect
PROBHD 5 mm PABBO BB/
PULPROG zg30
TD 32768
SOLVENT CDCl3
NS 16
DS 0
SWH 8012.820 Hz
FIDRES 0.244532 Hz
AQ 2.0447731 sec
RG 205.92
DW 62.400 usec
DE 16.68 usec
TE 300.0 K
D1 2.00000000 sec
TD0 1
======== CHANNEL f1 ========
SFO1 400.1324008 MHz
NUC1 1H
P1 13.30 usec
SI 16384
SF 400.1300000 MHz
WDW EM
SSB 0
LB 0.00 Hz
GB 0
PC 1.00
1.074
0.188
1.000
3.510
3.366
2.041
2.184
0.997
0.939
2.375
7.200
15
14
13
12
11
10
9
8
7
6
5
4
3
2
1
ppm

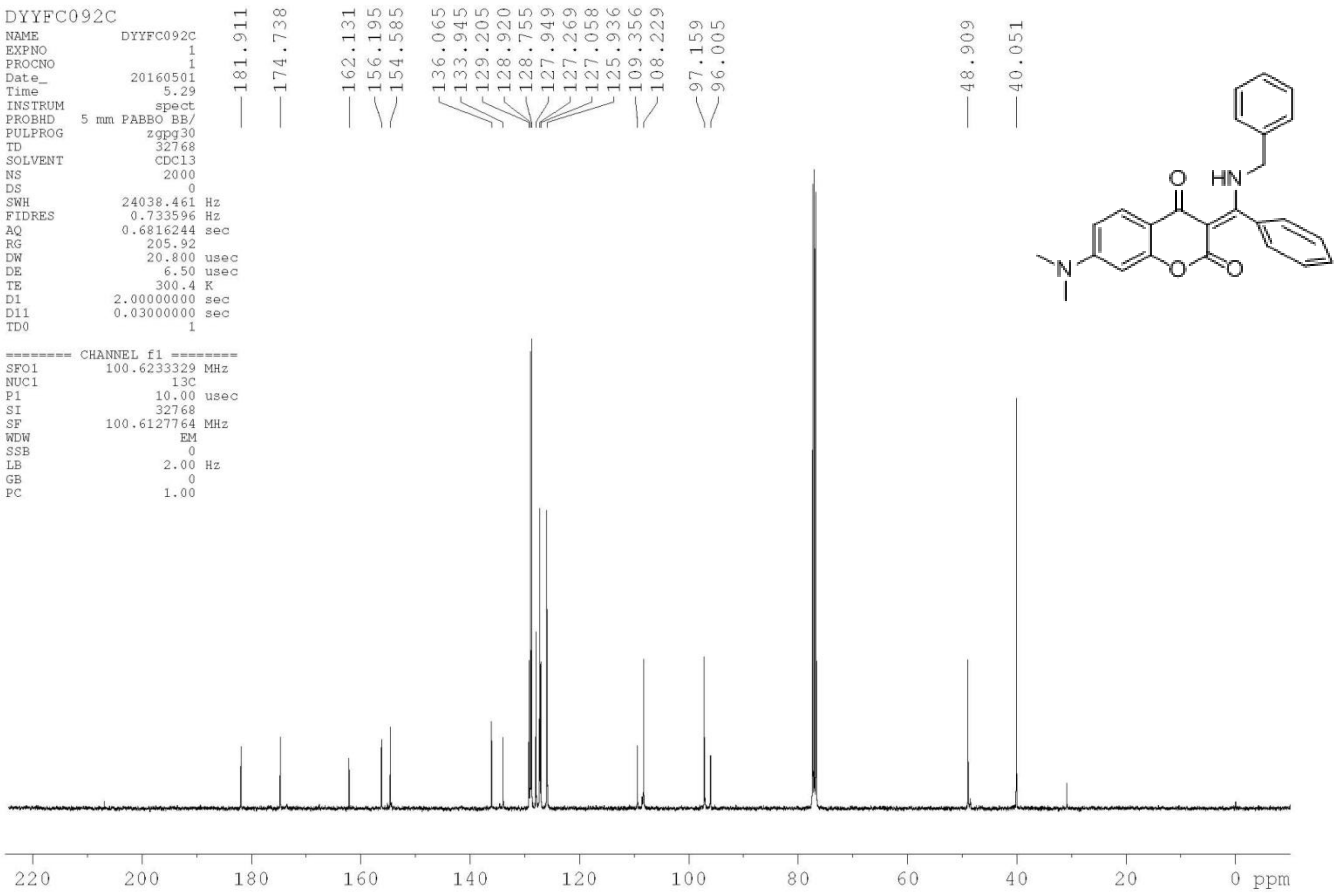
DYYFC092C
NAME DYYFC092C
EXPNO 1
PROCNO 1
Date_ 20160501
Time 5.29
INSTRUM spect
PROBHD 5 mm PABBO BB/
PULPROG zgpg30
TD 32768
SOLVENT CDCl3
NS 2000
DS 0
SWH 24038.461 Hz
FIDRES 0.733596 Hz
AQ 0.6816244 sec
RG 205.92
DW 20.800 usec
DE 6.50 usec
TE 300.4 K
D1 2.00000000 sec
D11 0.03000000 sec
TD0 1
======== CHANNEL f1 ========
SFO1 100.6233329 MHz
NUC1 13C
P1 10.00 usec
SI 32768
SF 100.6127764 MHz
WDW EM
SSB 0
LB 2.00 Hz
GB 0
PC 1.00
181.911
174.738
162.131
156.195
154.585
136.065
133.945
129.205
128.920
128.755
127.949
127.269
127.058
125.936
109.356
108.229
97.159
96.005
48.909
40.051
HN
O
O
O
N
220
200
180
160
140
120
100
80
60
40
20
0 ppm

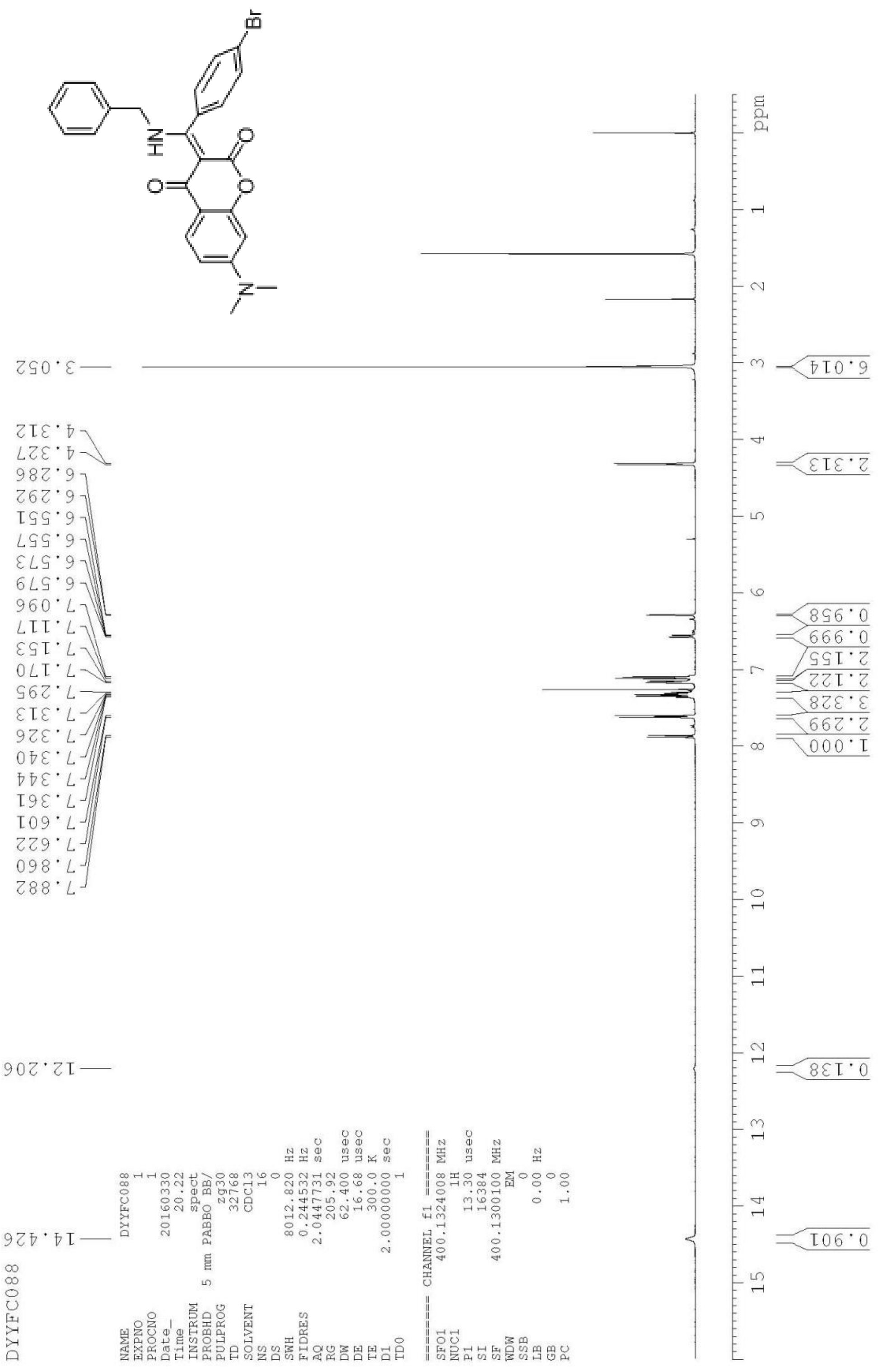
DYYFC088
14.426
12.206
7.882
7.860
7.622
7.601
7.361
7.344
7.340
7.326
7.313
7.295
7.170
7.153
7.117
7.096
6.579
6.573
6.557
6.551
6.292
6.286
4.327
4.312
3.052
NAME DYYFC088
EXPNO 1
PROCNO 1
Date_ 20160330
Time 20.22
INSTRUM spect
PROBHD 5 mm PABBO BB/
PULPROG zg30
TD 32768
SOLVENT CDCl3
NS 16
DS 0
SWH 8012.820 Hz
FIDRES 0.244532 Hz
AQ 2.0447731 sec
RG 205.92
DW 62.400 usec
DE 16.68 usec
TE 300.0 K
D1 2.00000000 sec
TD0 1
======== CHANNEL f1 ========
SFO1 400.1324008 MHz
NUC1 1H
P1 13.30 usec
SI 16384
SF 400.1300100 MHz
WDW EM
SSB 0
LB 0.00 Hz
GB 0
PC 1.00
0.901
0.138
1.000
2.299
3.328
2.122
2.155
0.999
0.958
2.313
6.014
ppm
HN
O
O
O
N
Br

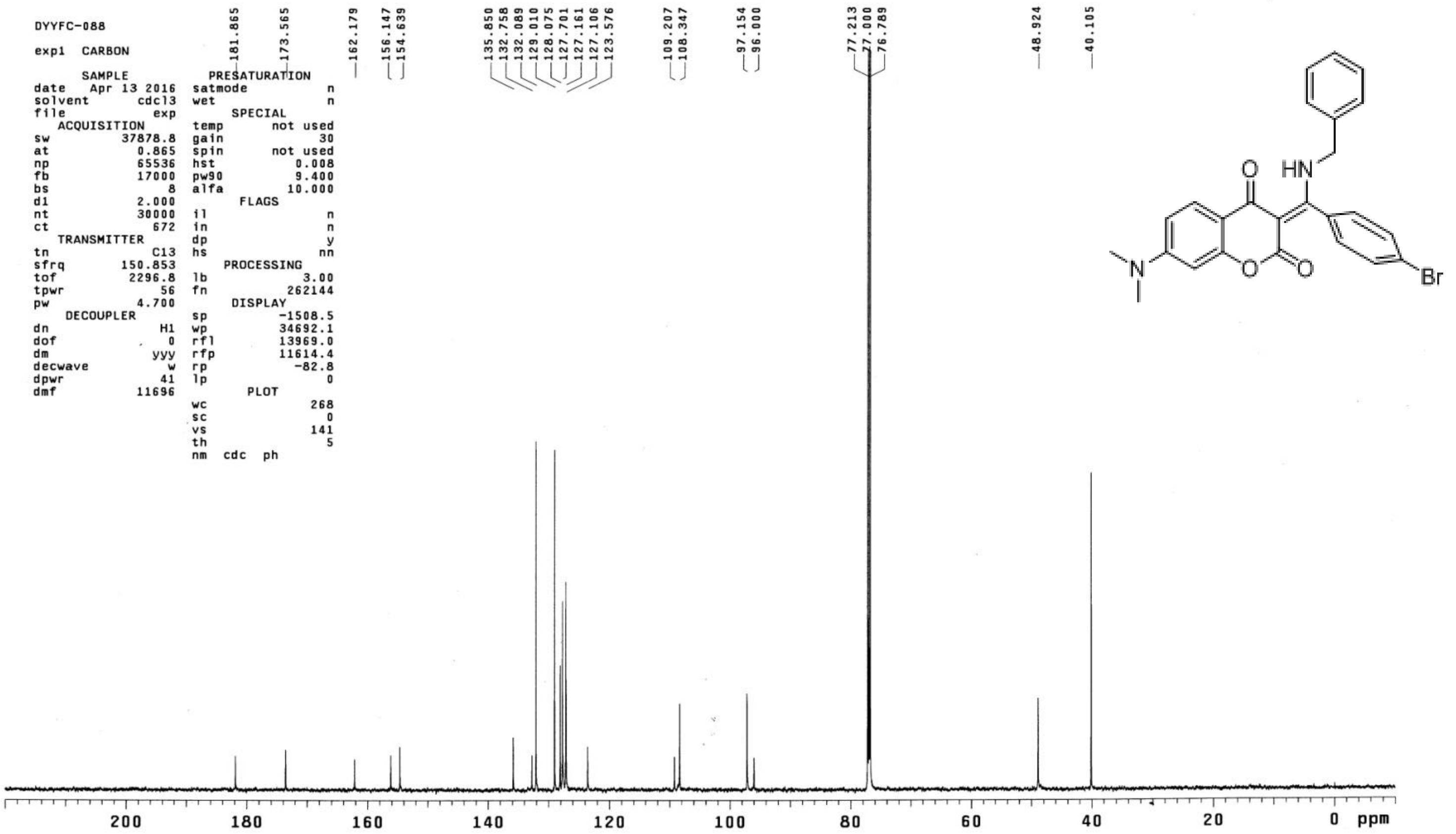
DYYFC-088
exp1 CARBON
SAMPLE
date Apr 13 2016
solvent cdcl3
file exp
ACQUISITION
sw 37878.8
at 0.865
np 65536
fb 17000
bs 8
d1 2.000
nt 30000
ct 672
TRANSMITTER
tn C13
sfrq 150.853
tof 2296.8
tpwr 56
pw 4.700
DECOUPLER
dn H1
dof 0
dm yyy
decwave w
dpwr 41
dmf 11696
PRESATURATION
satmode n
wet n
SPECIAL
temp not used
gain 30
spin not used
hst 0.008
pw90 9.400
alfa 10.000
FLAGS
il n
in n
dp y
hs nn
PROCESSING
lb 3.00
fn 262144
DISPLAY
sp -1508.5
wp 34692.1
rfl 13969.0
rfp 11614.4
rp -82.8
lp 0
PLOT
wc 268
sc 0
vs 141
th 5
nm cdc ph
181.865
173.565
162.179
156.147
154.639
135.850
132.758
132.089
129.010
128.075
127.701
127.161
127.106
123.576
109.207
108.347
97.154
96.000
77.213
77.000
76.789
48.924
40.105
O
HN
N
O
O
Br
200
180
160
140
120
100
80
60
40
20
0 ppm

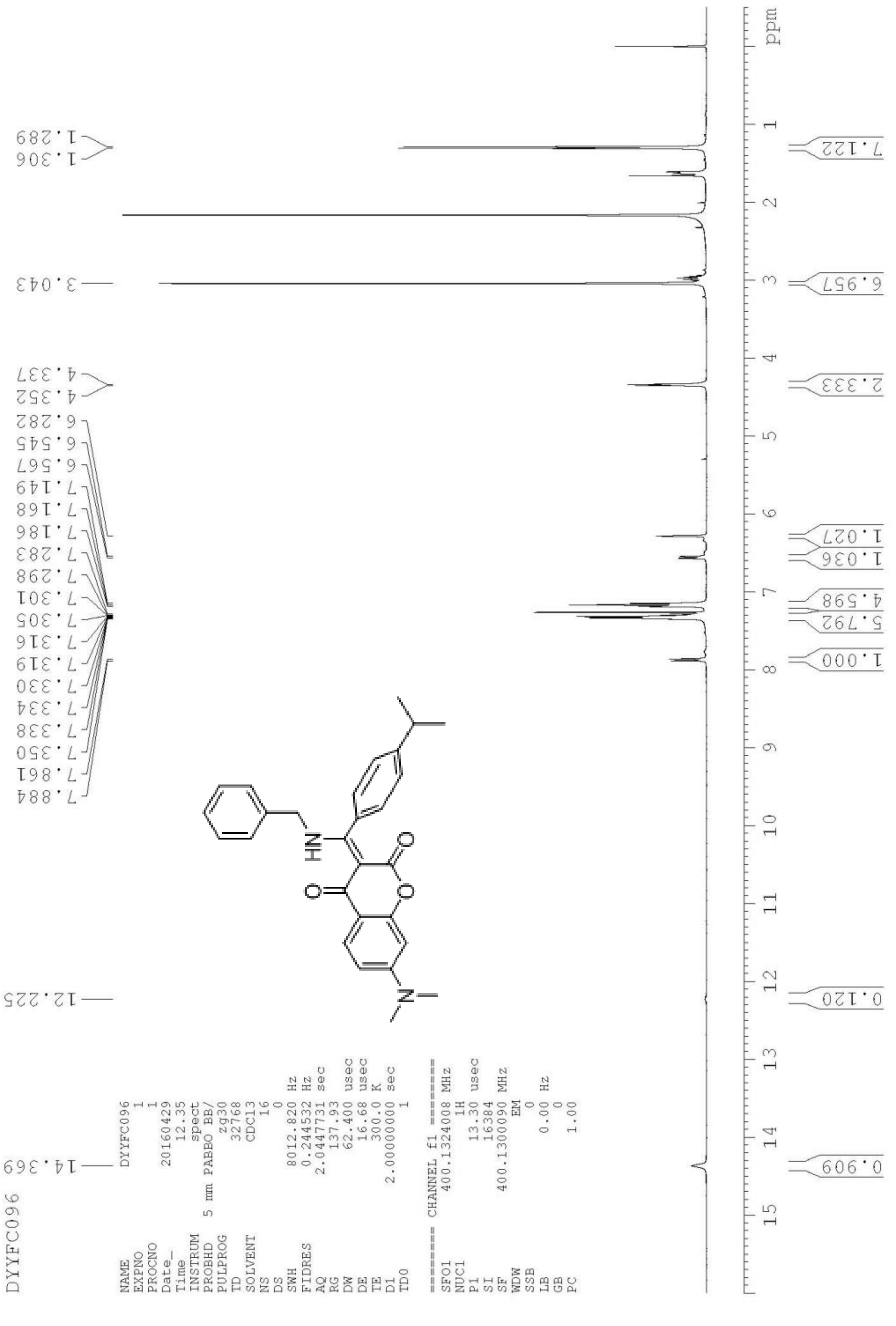
DYYFC096
14.369
12.225
7.884
7.861
7.350
7.338
7.334
7.330
7.319
7.316
7.305
7.301
7.298
7.283
7.186
7.168
7.149
6.567
6.545
6.282
4.352
4.337
3.043
1.306
1.289
NAME DYYFC096
EXPNO 1
PROCNO 1
Date_ 20160429
Time 12.35
INSTRUM spect
PROBHD 5 mm PABBO BB/
PULPROG zg30
TD 32768
SOLVENT CDCl3
NS 16
DS 0
SWH 8012.820 Hz
FIDRES 0.244532 Hz
AQ 2.0447731 sec
RG 137.93
DW 62.400 usec
DE 16.68 usec
TE 300.0 K
D1 2.00000000 sec
TD0 1
======== CHANNEL f1 ========
SFO1 400.1324008 MHz
NUC1 1H
P1 13.30 usec
SI 16384
SF 400.1300090 MHz
WDW EM
SSB 0
LB 0.00 Hz
GB 0
PC 1.00
HN
O
N
ppm
0.909
0.120
1.000
5.792
4.598
1.036
1.027
2.333
6.957
7.122

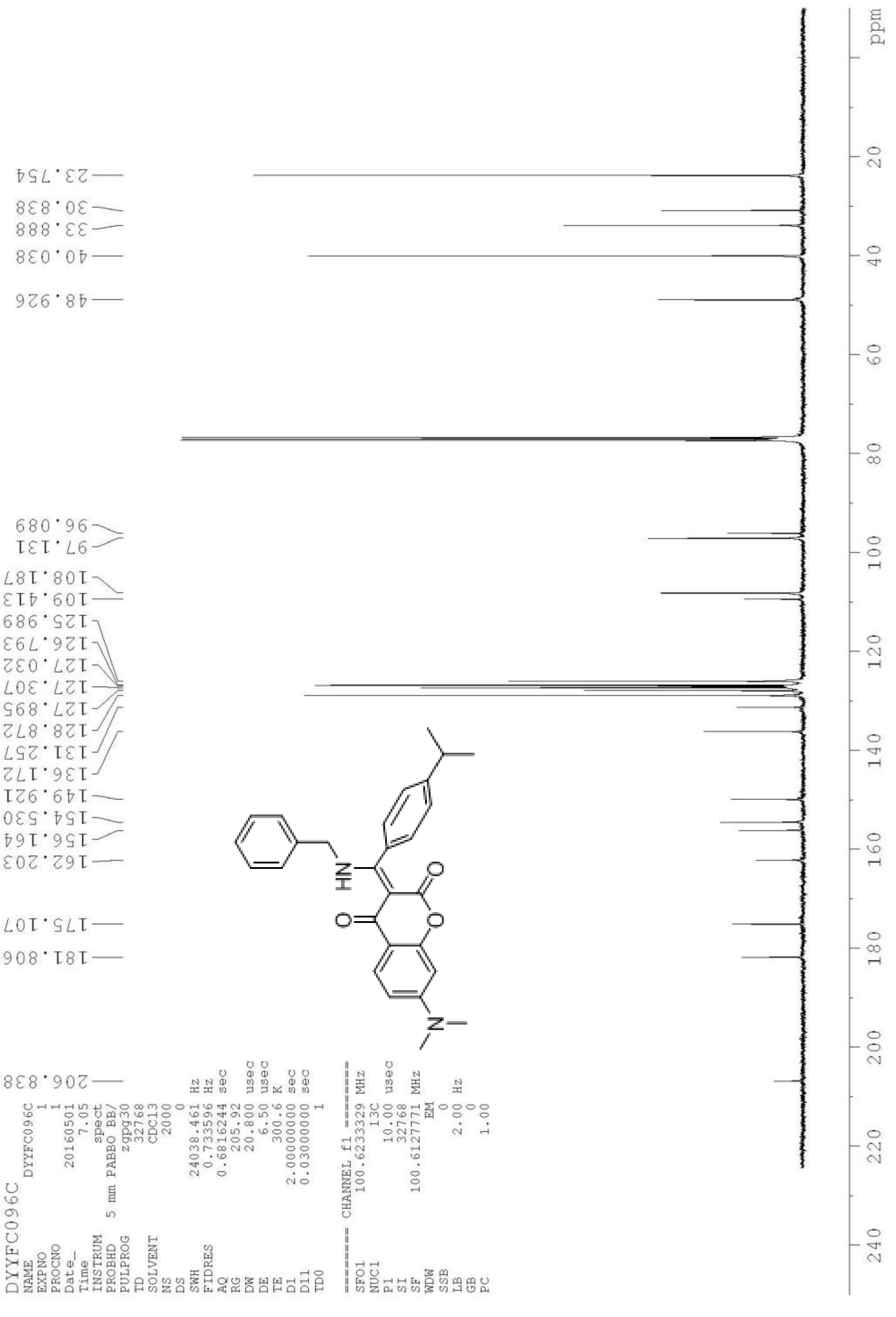

DYYFC096C
NAME DYYFC096C
EXPNO 1
PROCNO 1
Date_ 20160501
Time 7.05
INSTRUM spect
PROBHD 5 mm PABBO BB/
PULPROG zgpg30
TD 32768
SOLVENT CDCl3
NS 2000
DS 0
SWH 24038.461 Hz
FIDRES 0.733596 Hz
AQ 0.6816244 sec
RG 205.92
DW 20.800 usec
DE 6.50 usec
TE 300.6 K
D1 2.00000000 sec
D11 0.03000000 sec
TD0 1
======== CHANNEL f1 ========
SFO1 100.6233329 MHz
NUC1 13C
P1 10.00 usec
SI 32768
SF 100.6127771 MHz
WDW EM
SSB 0
LB 2.00 Hz
GB 0
PC 1.00
206.838
181.806
175.107
162.203
156.164
154.530
149.921
136.172
131.257
128.872
127.895
127.307
127.032
126.793
125.989
109.413
108.187
97.131
96.089
48.926
40.038
33.888
30.838
23.754
HN
O
N
ppm
240
220
200
180
160
140
120
100
80
60
40
20

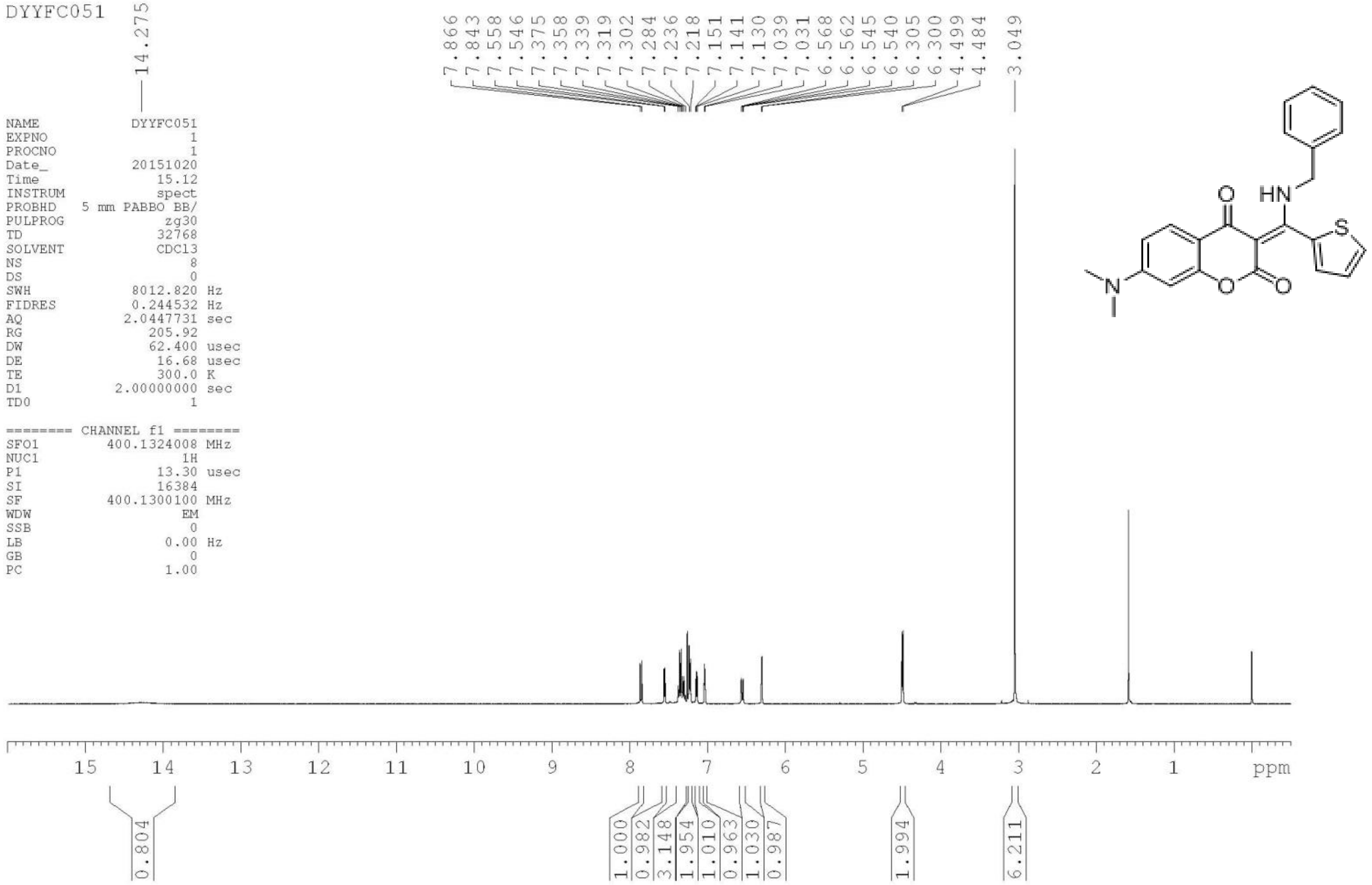
DYYFC051
14.275
7.866
7.843
7.558
7.546
7.375
7.358
7.339
7.319
7.302
7.284
7.236
7.218
7.151
7.141
7.130
7.039
7.031
6.568
6.562
6.545
6.540
6.305
6.300
4.499
4.484
3.049
NAME DYYFC051
EXPNO 1
PROCNO 1
Date_ 20151020
Time 15.12
INSTRUM spect
PROBHD 5 mm PABBO BB/
PULPROG zg30
TD 32768
SOLVENT CDCl3
NS 8
DS 0
SWH 8012.820 Hz
FIDRES 0.244532 Hz
AQ 2.0447731 sec
RG 205.92
DW 62.400 usec
DE 16.68 usec
TE 300.0 K
D1 2.00000000 sec
TD0 1
======== CHANNEL f1 ========
SFO1 400.1324008 MHz
NUC1 1H
P1 13.30 usec
SI 16384
SF 400.1300100 MHz
WDW EM
SSB 0
LB 0.00 Hz
GB 0
PC 1.00
O
HN
S
N
O
O
15
14
13
12
11
10
9
8
7
6
5
4
3
2
1
ppm
0.804
1.000
0.982
3.148
1.954
1.010
0.963
1.030
0.987
1.994
6.211

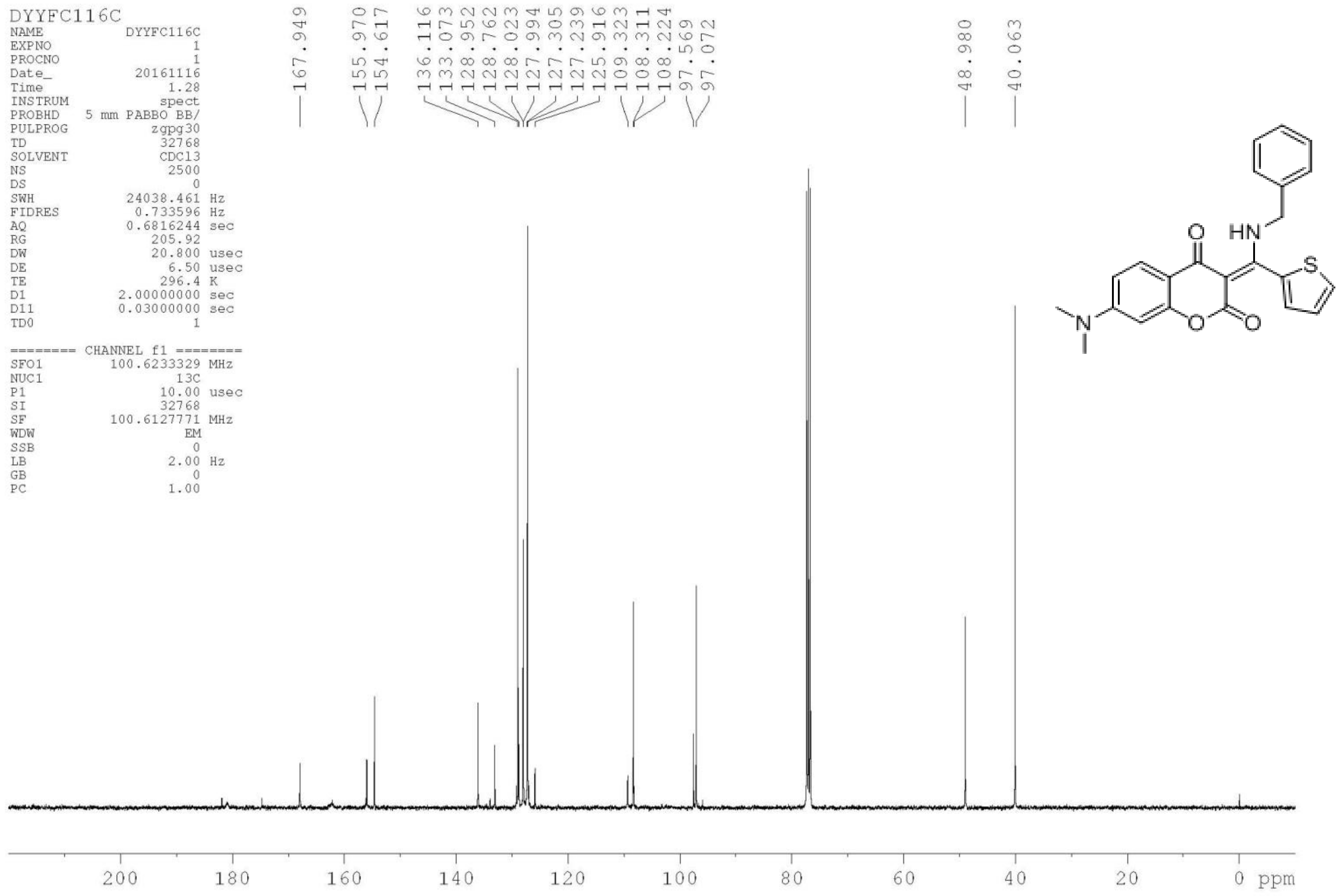
DYYFC116C
NAME DYYFC116C
EXPNO 1
PROCNO 1
Date_ 20161116
Time 1.28
INSTRUM spect
PROBHD 5 mm PABBO BB/
PULPROG zgpg30
TD 32768
SOLVENT CDCl3
NS 2500
DS 0
SWH 24038.461 Hz
FIDRES 0.733596 Hz
AQ 0.6816244 sec
RG 205.92
DW 20.800 usec
DE 6.50 usec
TE 296.4 K
D1 2.00000000 sec
D11 0.03000000 sec
TD0 1
======== CHANNEL f1 ========
SFO1 100.6233329 MHz
NUC1 13C
P1 10.00 usec
SI 32768
SF 100.6127771 MHz
WDW EM
SSB 0
LB 2.00 Hz
GB 0
PC 1.00
167.949
155.970
154.617
136.116
133.073
128.952
128.762
128.023
127.994
127.305
127.239
125.916
109.323
108.311
108.224
97.569
97.072
48.980
40.063
O
HN
S
N
O
O
200
180
160
140
120
100
80
60
40
20
0 ppm

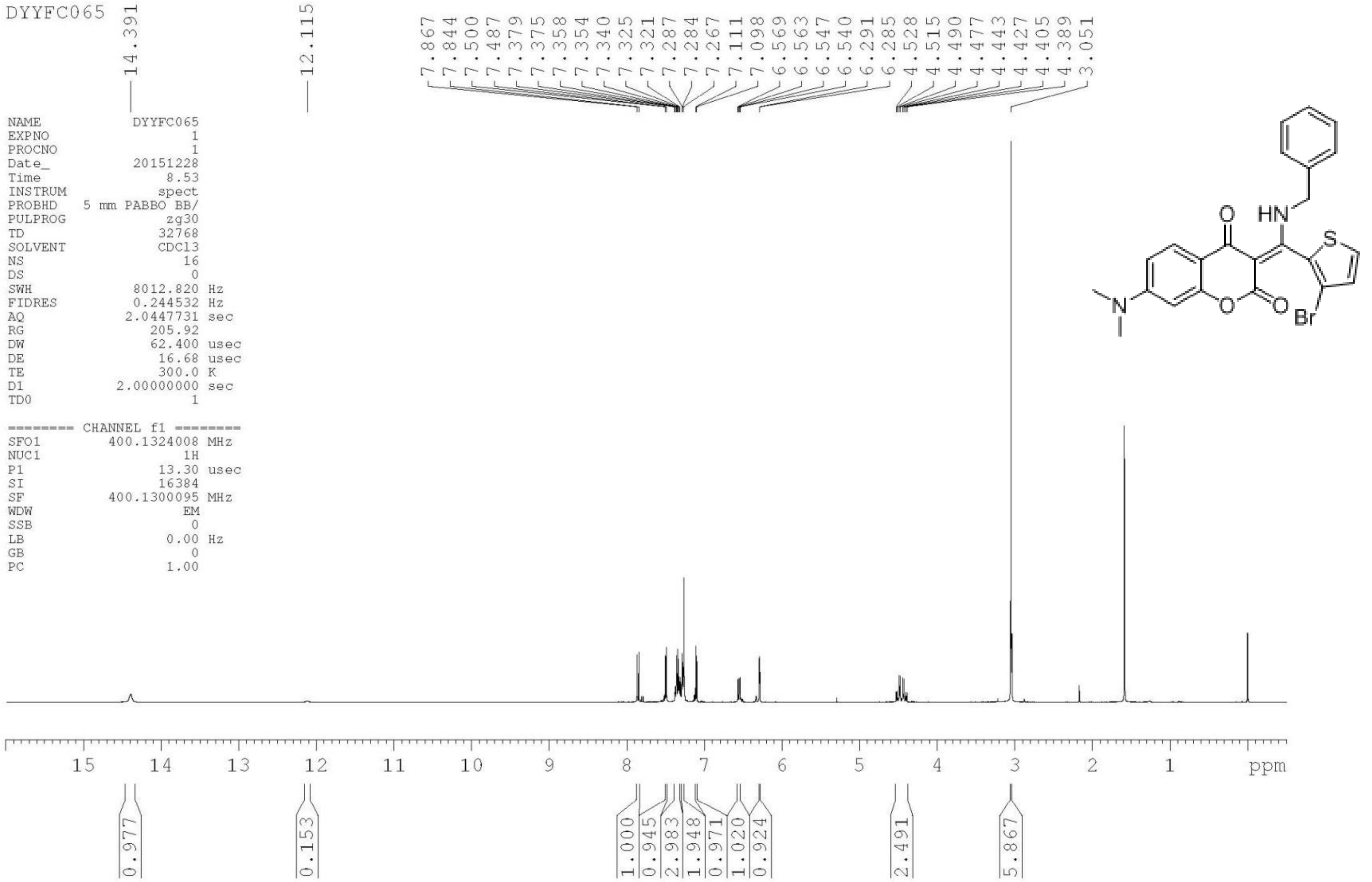
DYYFC065
14.391
12.115
7.867 7.844 7.500 7.487 7.379 7.375 7.358 7.354 7.340 7.325 7.321 7.287 7.284 7.267 7.111 7.098 6.569 6.563 6.547 6.540 6.291 6.285 4.528 4.515 4.490 4.477 4.443 4.427 4.405 4.389 3.051
NAME DYYFC065
EXPNO 1
PROCNO 1
Date_ 20151228
Time 8.53
INSTRUM spect
PROBHD 5 mm PABBO BB/
PULPROG zg30
TD 32768
SOLVENT CDCl3
NS 16
DS 0
SWH 8012.820 Hz
FIDRES 0.244532 Hz
AQ 2.0447731 sec
RG 205.92
DW 62.400 usec
DE 16.68 usec
TE 300.0 K
D1 2.00000000 sec
TD0 1
======== CHANNEL f1 ========
SFO1 400.1324008 MHz
NUC1 1H
P1 13.30 usec
SI 16384
SF 400.1300095 MHz
WDW EM
SSB 0
LB 0.00 Hz
GB 0
PC 1.00
HN
O
S
N
O
O
Br
15 14 13 12 11 10 9 8 7 6 5 4 3 2 1 ppm
0.977
0.153
1.000 0.945 2.983 1.948 0.971 1.020 0.924
2.491
5.867

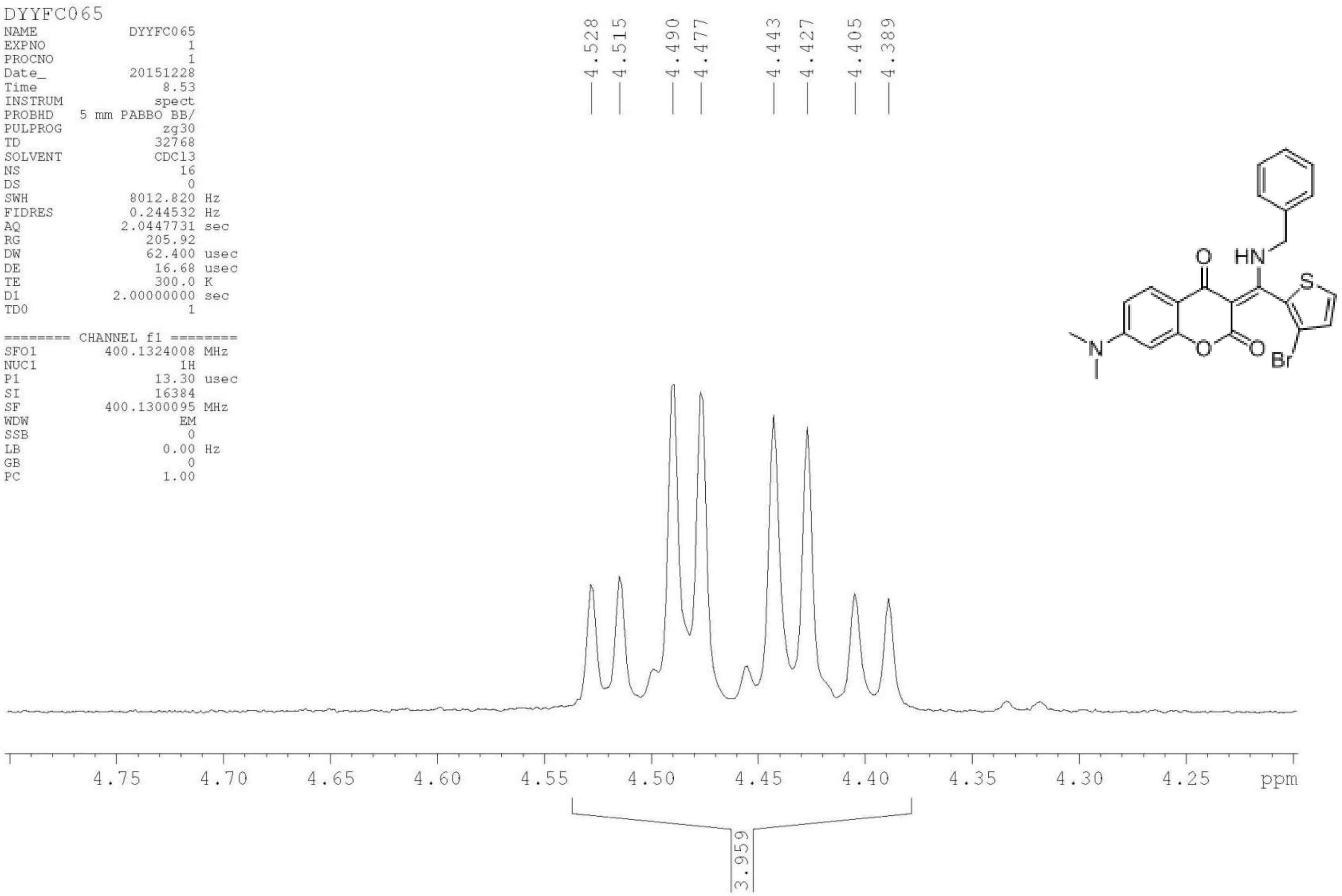
DYYFC065
NAME DYYFC065
EXPNO 1
PROCNO 1
Date_ 20151228
Time 8.53
INSTRUM spect
PROBHD 5 mm PABBO BB/
PULPROG zg30
TD 32768
SOLVENT CDCl3
NS 16
DS 0
SWH 8012.820 Hz
FIDRES 0.244532 Hz
AQ 2.0447731 sec
RG 205.92
DW 62.400 usec
DE 16.68 usec
TE 300.0 K
D1 2.00000000 sec
TD0 1
======== CHANNEL f1 ========
SFO1 400.1324008 MHz
NUC1 1H
P1 13.30 usec
SI 16384
SF 400.1300095 MHz
WDW EM
SSB 0
LB 0.00 Hz
GB 0
PC 1.00
4.528
4.515
4.490
4.477
4.443
4.427
4.405
4.389
HN
S
O
O
O
N
Br
4.75
4.70
4.65
4.60
4.55
4.50
4.45
4.40
4.35
4.30
4.25
ppm
3.959

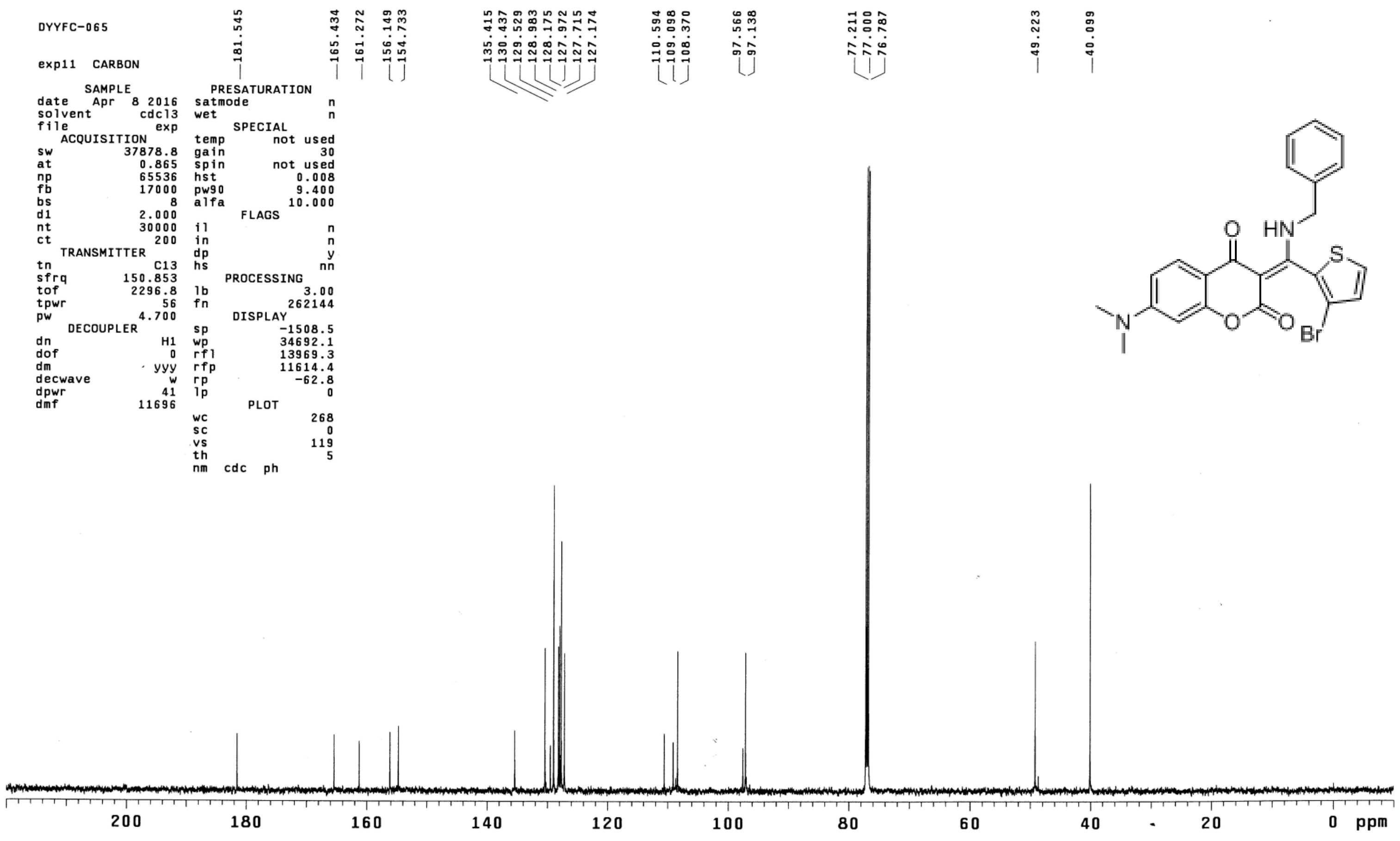

DYYFC-065
exp11 CARBON
SAMPLE
date Apr 8 2016
solvent cdcl3
file exp
ACQUISITION
sw 37878.8
at 0.865
np 65536
fb 17000
bs 8
d1 2.000
nt 30000
ct 200
TRANSMITTER
tn C13
sfrq 150.853
tof 2296.8
tpwr 56
pw 4.700
DECOUPLER
dn H1
dof 0
dm yyy
decwave w
dpwr 41
dmf 11696
PRESATURATION
satmode n
wet n
SPECIAL
temp not used
gain 30
spin not used
hst 0.008
pw90 9.400
alfa 10.000
FLAGS
il n
in n
dp y
hs nn
PROCESSING
lb 3.00
fn 262144
DISPLAY
sp -1508.5
wp 34692.1
rfl 13969.3
rfp 11614.4
rp -62.8
lp 0
PLOT
wc 268
sc 0
vs 119
th 5
nm cdc ph
181.545
165.434
161.272
156.149
154.733
135.415
130.437
129.529
128.983
128.175
127.972
127.715
127.174
110.594
109.098
108.370
97.566
97.138
77.211
77.000
76.787
49.223
40.099
O
HN
S
N
O
O
Br
200
180
160
140
120
100
80
60
40
20
0 ppm

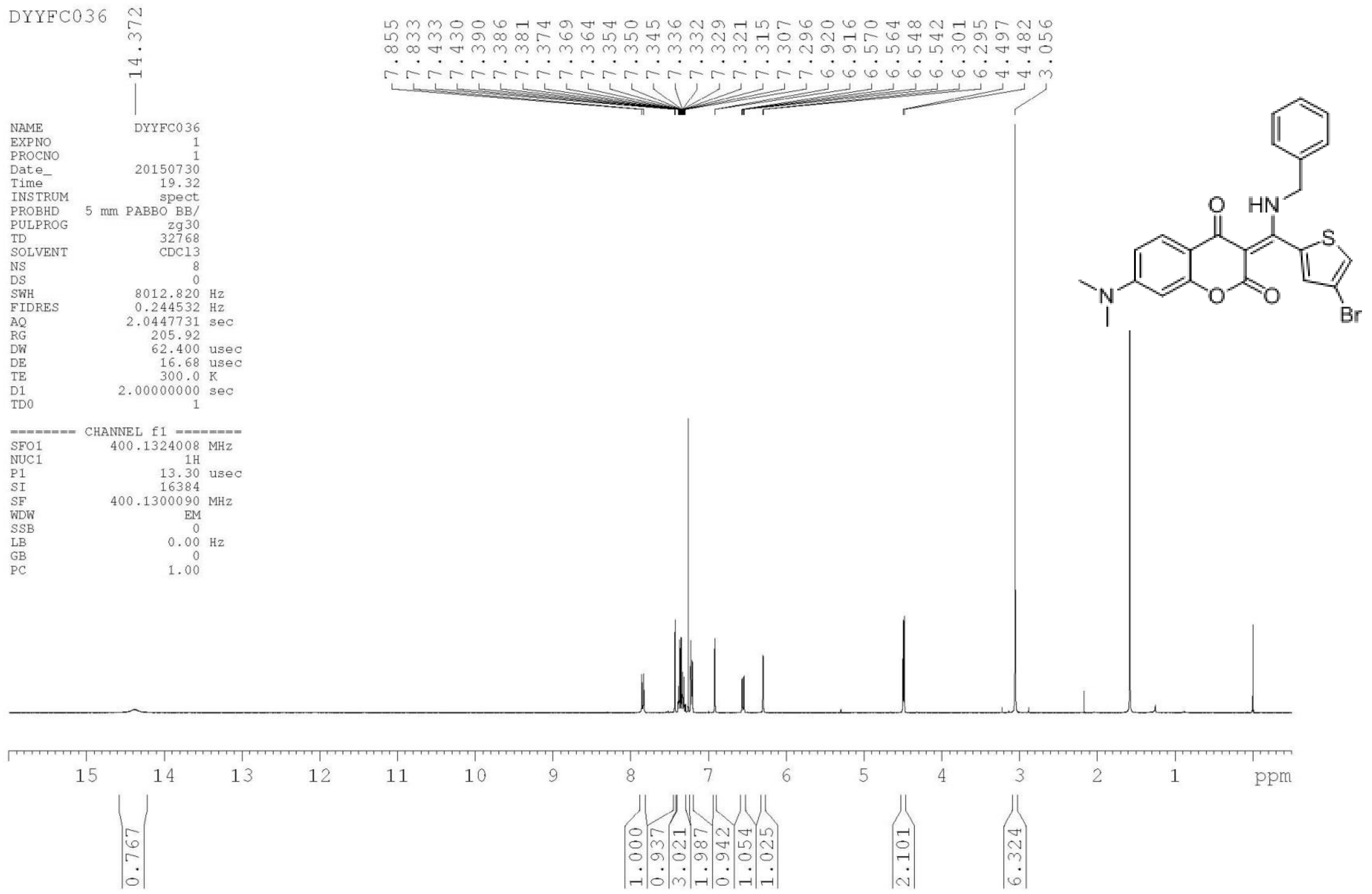
DYYFC036
14.372
7.855
7.833
7.433
7.430
7.390
7.386
7.381
7.374
7.369
7.364
7.354
7.350
7.345
7.336
7.332
7.329
7.321
7.315
7.307
7.296
6.920
6.916
6.570
6.564
6.548
6.542
6.301
6.295
4.497
4.482
3.056
NAME DYYFC036
EXPNO 1
PROCNO 1
Date_ 20150730
Time 19.32
INSTRUM spect
PROBHD 5 mm PABBO BB/
PULPROG zg30
TD 32768
SOLVENT CDCl3
NS 8
DS 0
SWH 8012.820 Hz
FIDRES 0.244532 Hz
AQ 2.0447731 sec
RG 205.92
DW 62.400 usec
DE 16.68 usec
TE 300.0 K
D1 2.00000000 sec
TD0 1
======== CHANNEL f1 ========
SFO1 400.1324008 MHz
NUC1 1H
P1 13.30 usec
SI 16384
SF 400.1300090 MHz
WDW EM
SSB 0
LB 0.00 Hz
GB 0
PC 1.00
O
HN
S
N
O
O
Br
15
14
13
12
11
10
9
8
7
6
5
4
3
2
1
ppm
0.767
1.000
0.937
3.021
1.987
0.942
1.054
1.025
2.101
6.324

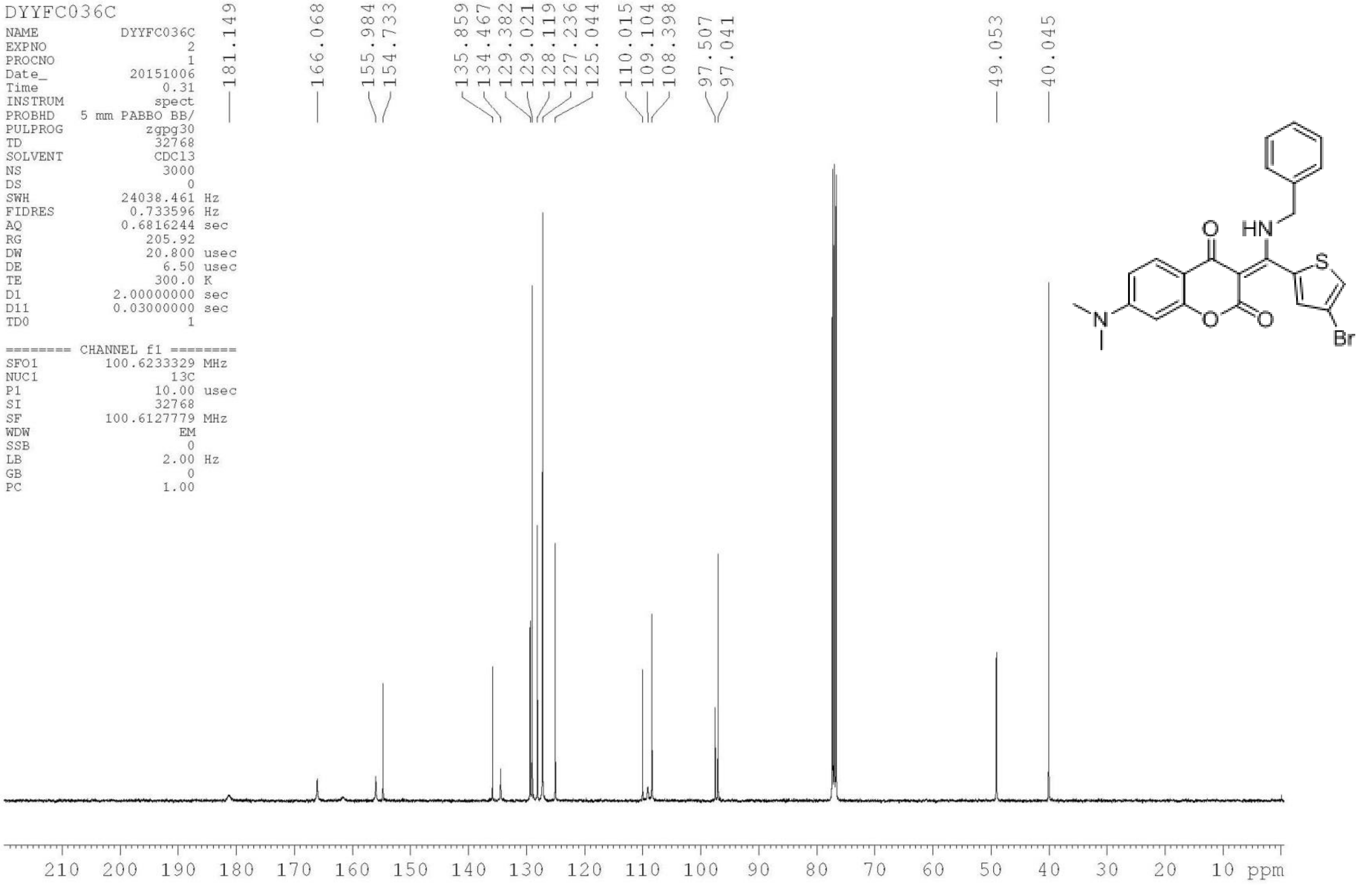
DYYFC036C
NAME DYYFC036C
EXPNO 2
PROCNO 1
Date_ 20151006
Time 0.31
INSTRUM spect
PROBHD 5 mm PABBO BB/
PULPROG zgpg30
TD 32768
SOLVENT CDCl3
NS 3000
DS 0
SWH 24038.461 Hz
FIDRES 0.733596 Hz
AQ 0.6816244 sec
RG 205.92
DW 20.800 usec
DE 6.50 usec
TE 300.0 K
D1 2.00000000 sec
D11 0.03000000 sec
TD0 1
======== CHANNEL f1 ========
SFO1 100.6233329 MHz
NUC1 13C
P1 10.00 usec
SI 32768
SF 100.6127779 MHz
WDW EM
SSB 0
LB 2.00 Hz
GB 0
PC 1.00
181.149
166.068
155.984
154.733
135.859
134.467
129.382
129.021
128.119
127.236
125.044
110.015
109.104
108.398
97.507
97.041
49.053
40.045
HN
O
O
O
N
S
Br
210 200 190 180 170 160 150 140 130 120 110 100 90 80 70 60 50 40 30 20 10 ppm

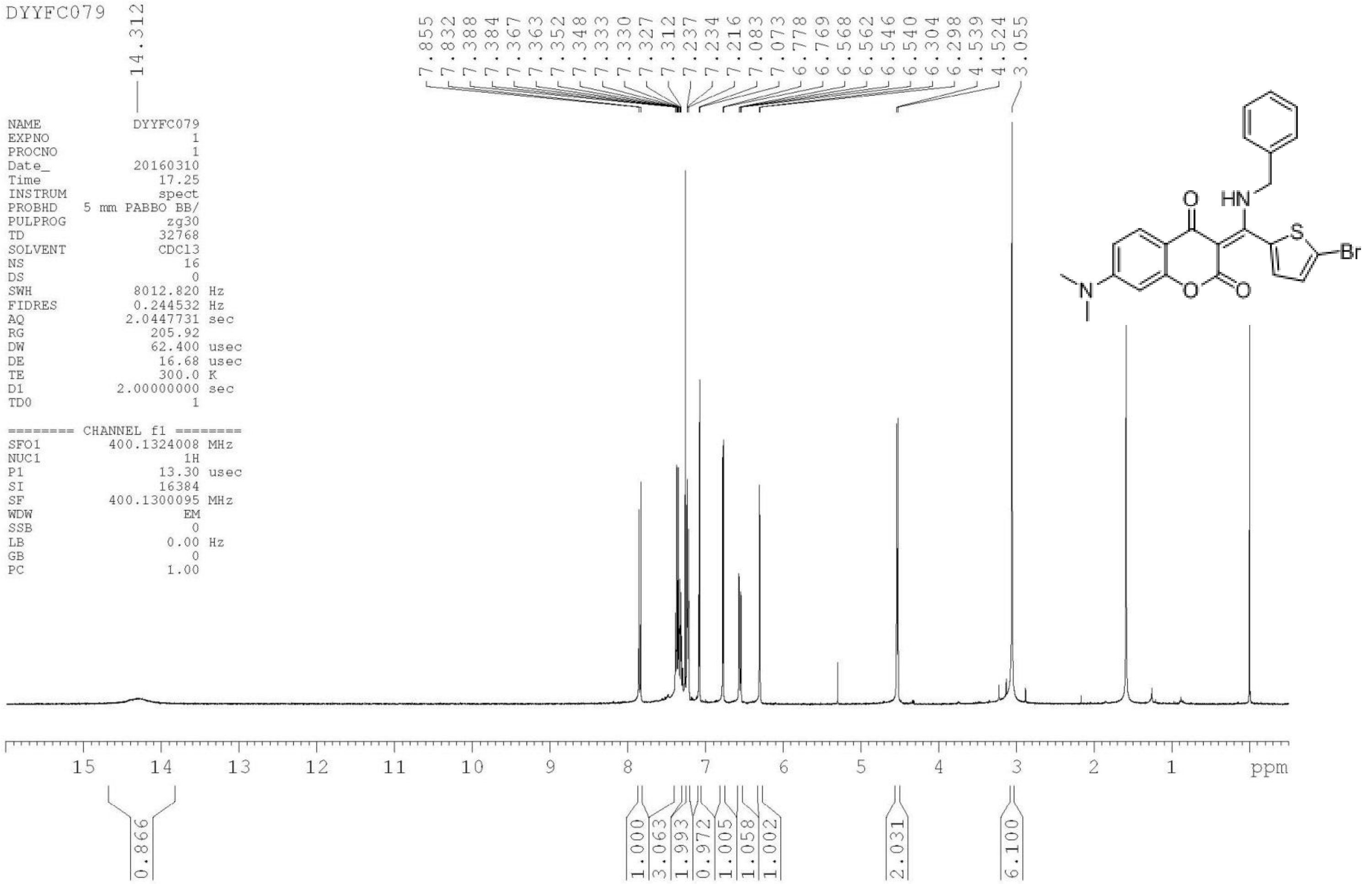
DYYFC079
14.312
7.855
7.832
7.388
7.384
7.367
7.363
7.352
7.348
7.333
7.330
7.327
7.312
7.237
7.234
7.216
7.083
7.073
6.778
6.769
6.568
6.562
6.546
6.540
6.304
6.298
4.539
4.524
3.055
NAME DYYFC079
EXPNO 1
PROCNO 1
Date_ 20160310
Time 17.25
INSTRUM spect
PROBHD 5 mm PABBO BB/
PULPROG zg30
TD 32768
SOLVENT CDCl3
NS 16
DS 0
SWH 8012.820 Hz
FIDRES 0.244532 Hz
AQ 2.0447731 sec
RG 205.92
DW 62.400 usec
DE 16.68 usec
TE 300.0 K
D1 2.00000000 sec
TD0 1
======== CHANNEL f1 ========
SFO1 400.1324008 MHz
NUC1 1H
P1 13.30 usec
SI 16384
SF 400.1300095 MHz
WDW EM
SSB 0
LB 0.00 Hz
GB 0
PC 1.00
O
HN
S
Br
N
O
O
15
14
13
12
11
10
9
8
7
6
5
4
3
2
1
ppm
0.866
1.000
3.063
1.993
0.972
1.005
1.058
1.002
2.031
6.100

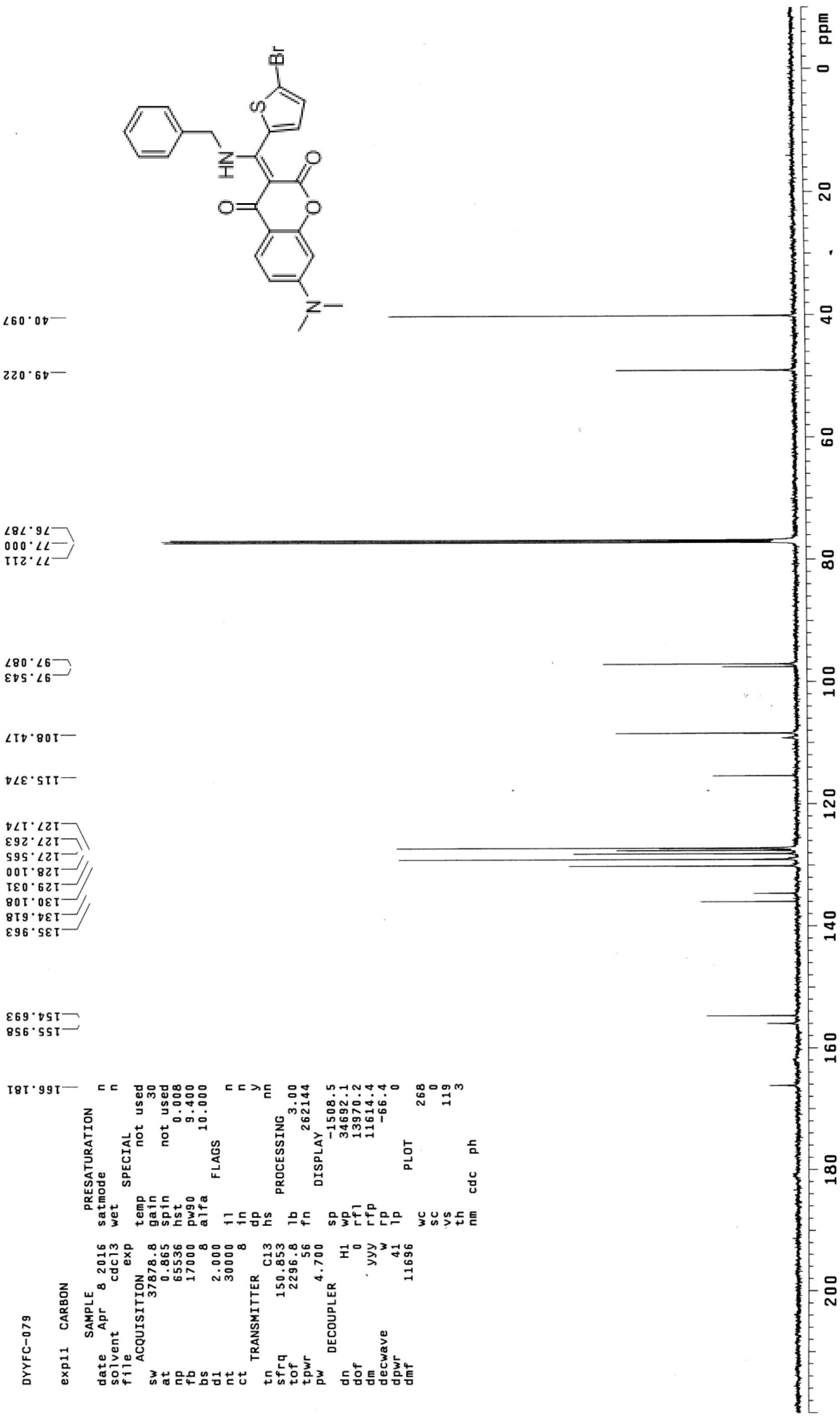
DYYFC-079
exp11 CARBON
SAMPLE
date Apr 8 2016
solvent cdcl3
file exp
ACQUISITION
sw 37878.8
at 0.865
np 65536
fb 17000
bs 8
d1 2.000
nt 30000
ct 8
TRANSMITTER
tn C13
sfrq 150.853
tof 2296.8
tpwr 56
pw 4.700
DECOUPLER
dn H1
dof 0
dm yyy
decwave W
dpwr 41
dmf 11696
PRESATURATION
satmode n
wet n
SPECIAL
temp not used
gain 30
spin not used
hst 0.008
pw90 9.400
alfa 10.000
FLAGS
il n
in n
dp y
hs nn
PROCESSING
lb 3.00
fn 262144
DISPLAY
sp -1508.5
wp 34692.1
rfl 13970.2
rfp 11614.4
rp -66.4
lp 0
PLOT
wc 268
sc 0
vs 119
th 3
nm cdc ph
166.181
155.958
154.693
135.963
134.618
130.108
129.031
128.100
127.565
127.263
127.174
115.374
108.417
97.543
97.087
77.211
77.000
76.787
49.022
40.097
Br
S
HN
O
O
O
N
200
180
160
140
120
100
80
60
40
20
0
ppm

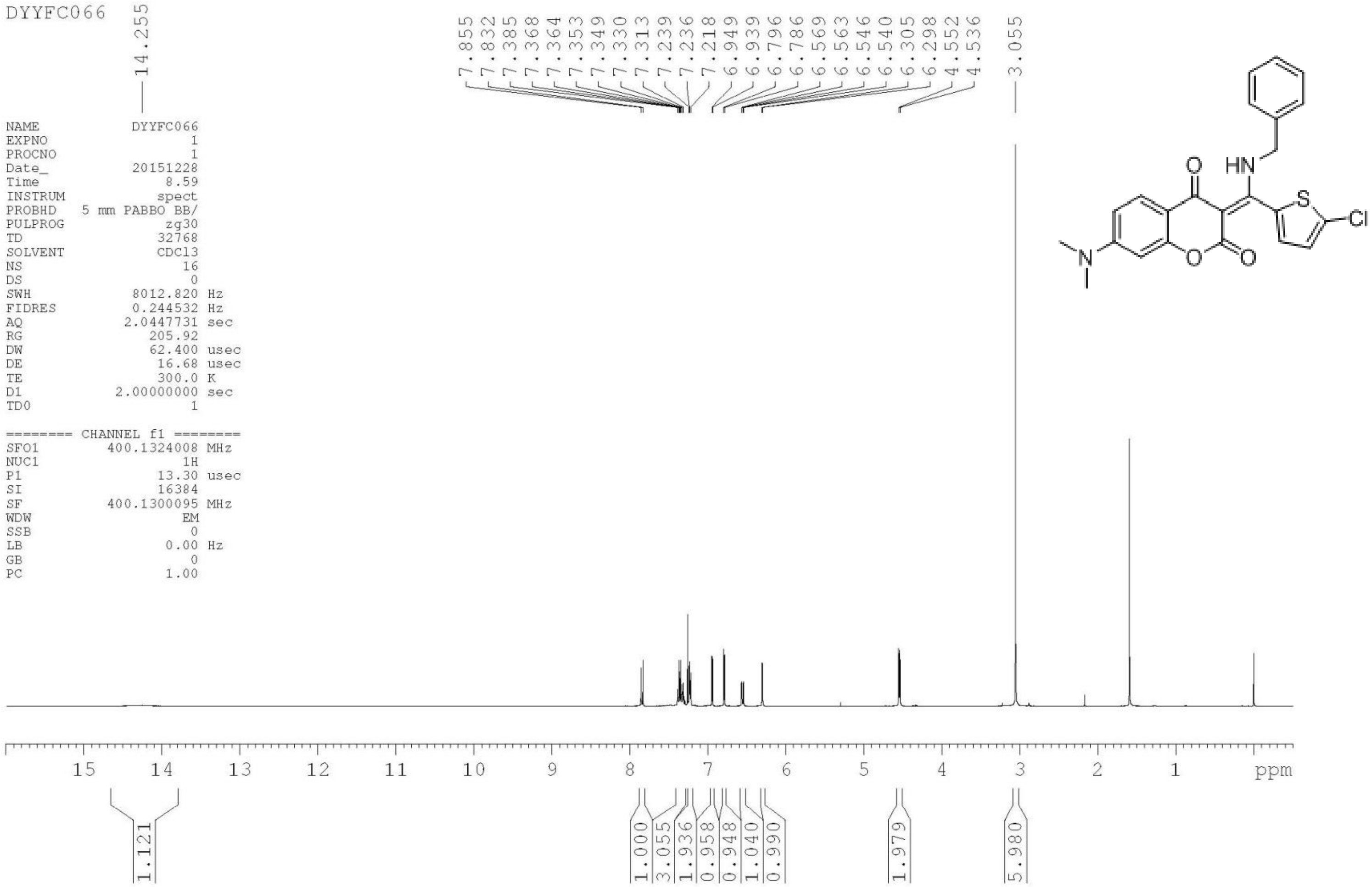
DYYFC066
14.255
7.855
7.832
7.385
7.368
7.364
7.353
7.349
7.330
7.313
7.239
7.236
7.218
6.949
6.939
6.796
6.786
6.569
6.563
6.546
6.540
6.305
6.298
4.552
4.536
3.055
NAME DYYFC066
EXPNO 1
PROCNO 1
Date_ 20151228
Time 8.59
INSTRUM spect
PROBHD 5 mm PABBO BB/
PULPROG zg30
TD 32768
SOLVENT CDCl3
NS 16
DS 0
SWH 8012.820 Hz
FIDRES 0.244532 Hz
AQ 2.0447731 sec
RG 205.92
DW 62.400 usec
DE 16.68 usec
TE 300.0 K
D1 2.00000000 sec
TD0 1
======== CHANNEL f1 ========
SFO1 400.1324008 MHz
NUC1 1H
P1 13.30 usec
SI 16384
SF 400.1300095 MHz
WDW EM
SSB 0
LB 0.00 Hz
GB 0
PC 1.00
HN
O
S
Cl
N
ppm
1.121
1.000
3.055
1.936
0.958
0.948
1.040
0.990
1.979
5.980

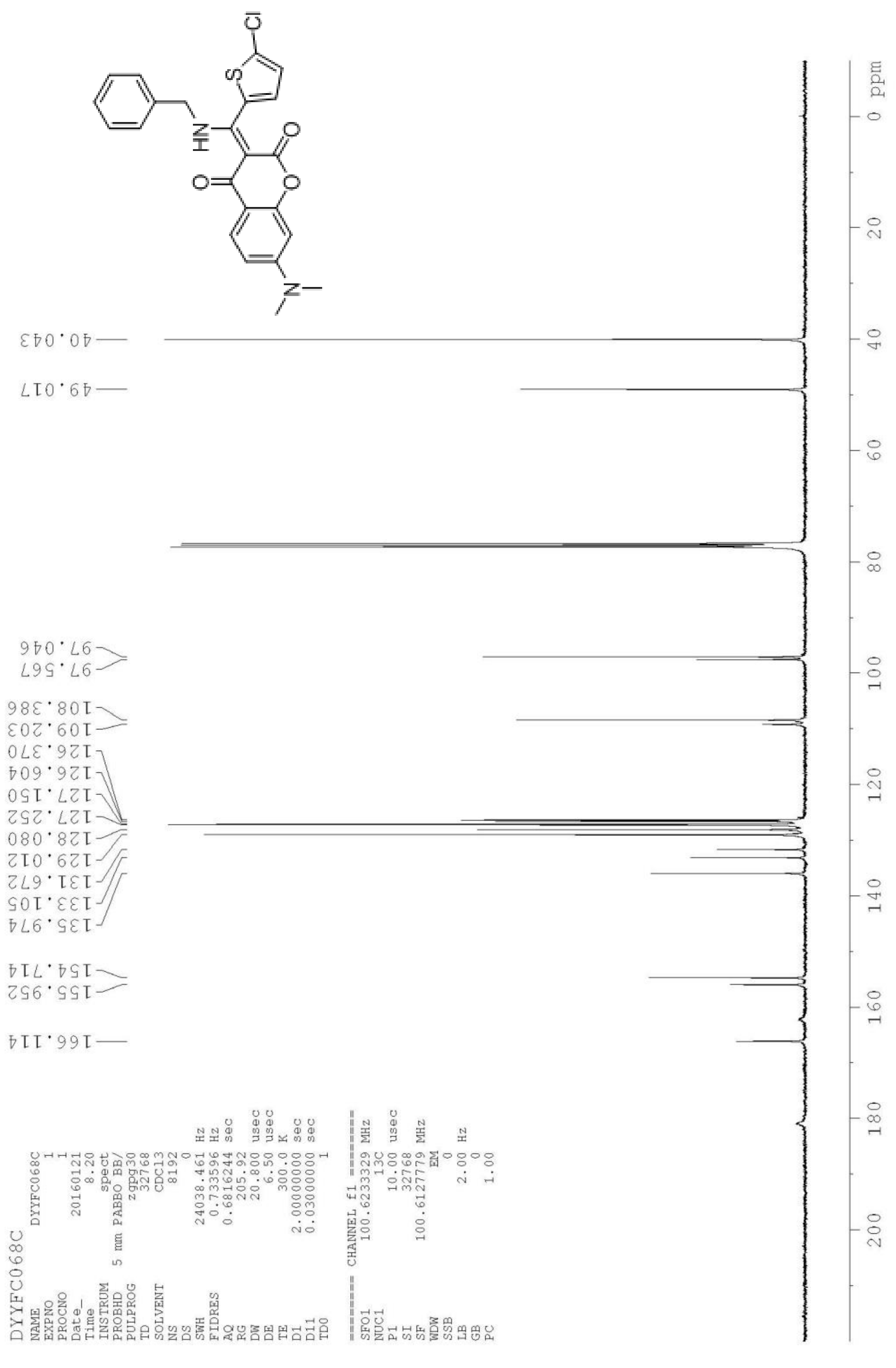
DYYFC068C
NAME DYYFC068C
EXPNO 1
PROCNO 1
Date_ 20160121
Time 8.20
INSTRUM spect
PROBHD 5 mm PABBO BB/
PULPROG zgpg30
TD 32768
SOLVENT CDCl3
NS 8192
DS 0
SWH 24038.461 Hz
FIDRES 0.733596 Hz
AQ 0.6816244 sec
RG 205.92
DW 20.800 usec
DE 6.50 usec
TE 300.0 K
D1 2.00000000 sec
D11 0.03000000 sec
TD0 1
======== CHANNEL f1 ========
SFO1 100.6233329 MHz
NUC1 13C
P1 10.00 usec
SI 32768
SF 100.6127779 MHz
WDW EM
SSB 0
LB 2.00 Hz
GB 0
PC 1.00
40.043
49.017
97.046
97.567
108.386
109.203
126.370
126.604
127.150
127.252
128.080
129.012
131.672
133.105
135.974
154.714
155.952
166.114
HN
O
S
Cl
N
ppm
0
20
40
60
80
100
120
140
160
180
200

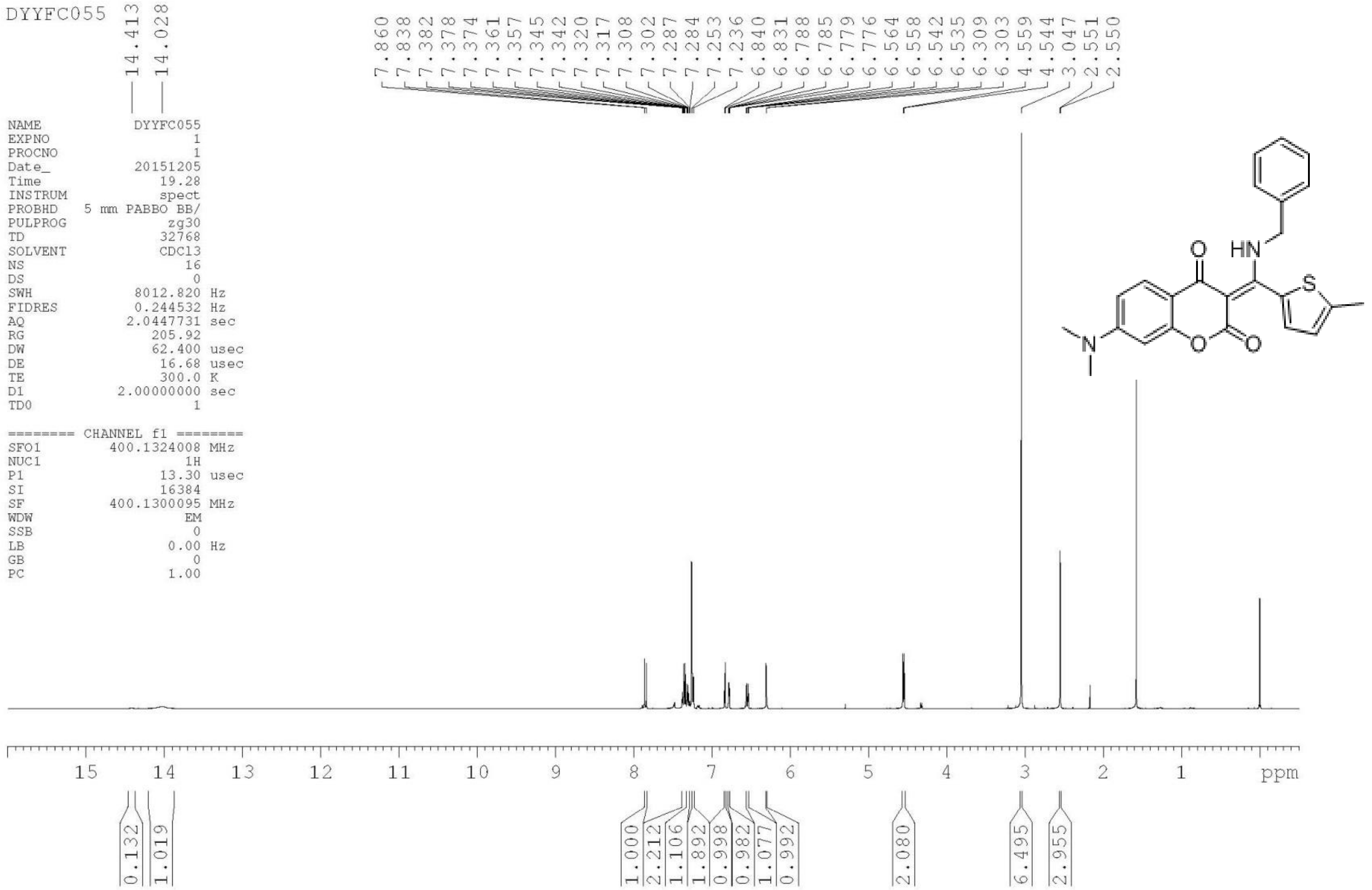
DYYFC055
14.413
14.028
7.860
7.838
7.382
7.378
7.374
7.361
7.357
7.345
7.342
7.320
7.317
7.308
7.302
7.287
7.284
7.253
7.236
6.840
6.831
6.788
6.785
6.779
6.776
6.564
6.558
6.542
6.535
6.309
6.303
4.559
4.544
3.047
2.551
2.550
NAME DYYFC055
EXPNO 1
PROCNO 1
Date_ 20151205
Time 19.28
INSTRUM spect
PROBHD 5 mm PABBO BB/
PULPROG zg30
TD 32768
SOLVENT CDCl3
NS 16
DS 0
SWH 8012.820 Hz
FIDRES 0.244532 Hz
AQ 2.0447731 sec
RG 205.92
DW 62.400 usec
DE 16.68 usec
TE 300.0 K
D1 2.00000000 sec
TD0 1
======== CHANNEL f1 ========
SFO1 400.1324008 MHz
NUC1 1H
P1 13.30 usec
SI 16384
SF 400.1300095 MHz
WDW EM
SSB 0
LB 0.00 Hz
GB 0
PC 1.00
HN
O
S
N
ppm
0.132
1.019
1.000
2.212
1.106
1.892
0.998
0.982
1.077
0.992
2.080
6.495
2.955

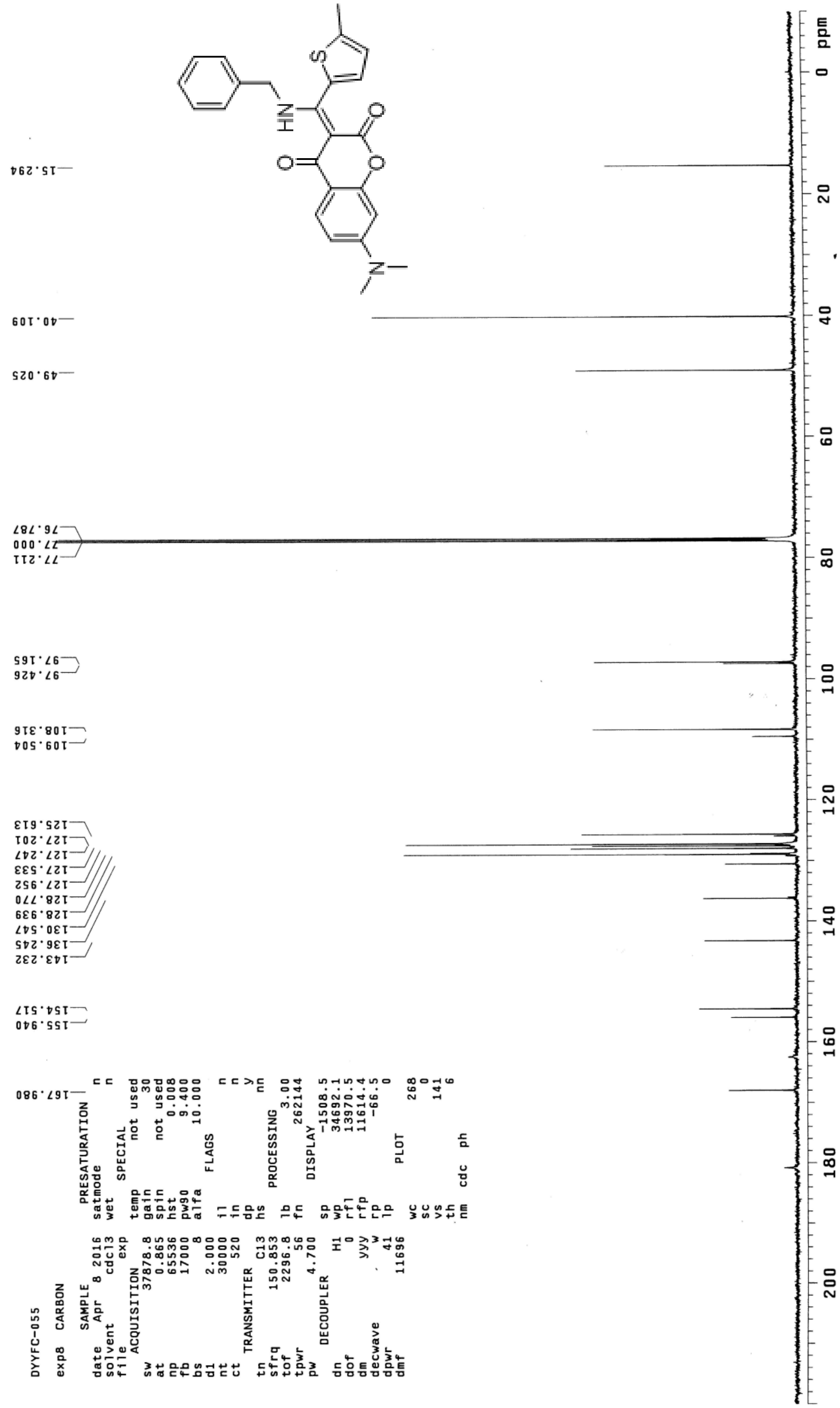
DYYFC-055
exp8 CARBON
SAMPLE
date Apr 8 2016
solvent cdcl3
file exp
ACQUISITION
sw 37878.8
at 0.865
np 65536
fb 17000
bs 8
d1 2.000
nt 30000
ct 520
TRANSMITTER
tn C13
sfrq 150.853
tof 2296.8
tpwr 56
pw 4.700
DECOUPLER
dn H1
dof 0
dm yyy
decwave w
dpwr 41
dmf 11696
PRESATURATION
satmode n
wet n
SPECIAL
temp not used
gain 30
spin not used
hst 0.008
pw90 9.400
alfa 10.000
FLAGS
il n
in n
dp y
hs nn
PROCESSING
lb 3.00
fn 262144
DISPLAY
sp -1508.5
wp 34692.1
rfl 13970.5
rfp 11614.4
rp -66.5
lp 0
PLOT
wc 268
sc 0
vs 141
th 6
nm cdc ph
167.980
155.940
154.517
143.232
136.245
130.547
128.939
128.770
127.952
127.533
127.247
127.201
125.613
109.504
108.316
97.426
97.165
77.211
77.000
76.787
49.025
40.109
15.294
ppm
0
20
40
60
80
100
120
140
160
180
200
S
HN
O
N

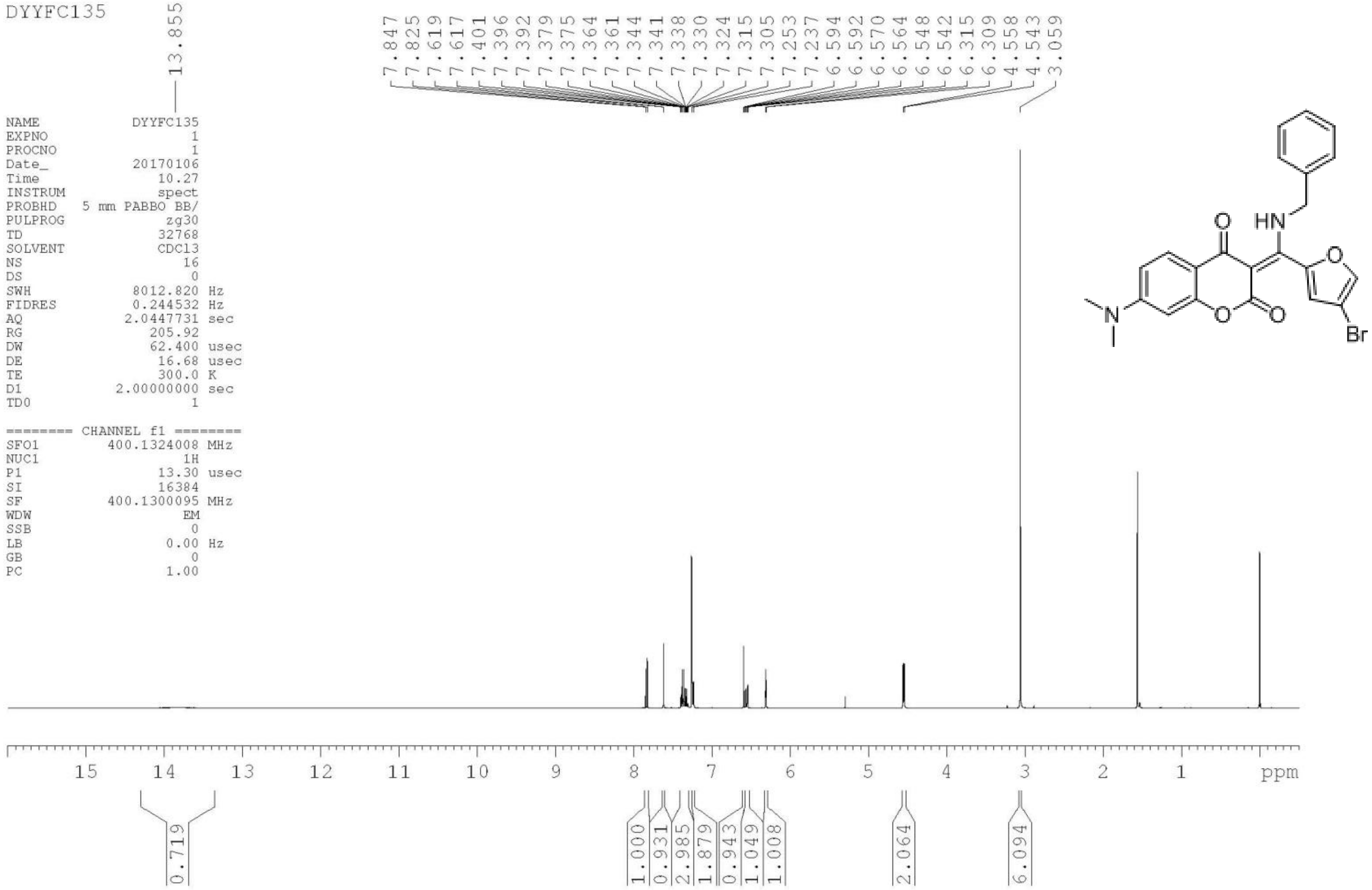
DYYFC135
13.855
7.847
7.825
7.619
7.617
7.401
7.396
7.392
7.379
7.375
7.364
7.361
7.344
7.341
7.338
7.330
7.324
7.315
7.305
7.253
7.237
6.594
6.592
6.570
6.564
6.548
6.542
6.315
6.309
4.558
4.543
3.059
NAME DYYFC135
EXPNO 1
PROCNO 1
Date_ 20170106
Time 10.27
INSTRUM spect
PROBHD 5 mm PABBO BB/
PULPROG zg30
TD 32768
SOLVENT CDCl3
NS 16
DS 0
SWH 8012.820 Hz
FIDRES 0.244532 Hz
AQ 2.0447731 sec
RG 205.92
DW 62.400 usec
DE 16.68 usec
TE 300.0 K
D1 2.00000000 sec
TD0 1
======== CHANNEL f1 ========
SFO1 400.1324008 MHz
NUC1 1H
P1 13.30 usec
SI 16384
SF 400.1300095 MHz
WDW EM
SSB 0
LB 0.00 Hz
GB 0
PC 1.00
HN
O
Br
N
15 14 13 12 11 10 9 8 7 6 5 4 3 2 1 ppm
0.719
1.000
0.931
2.985
1.879
0.943
1.049
1.008
2.064
6.094

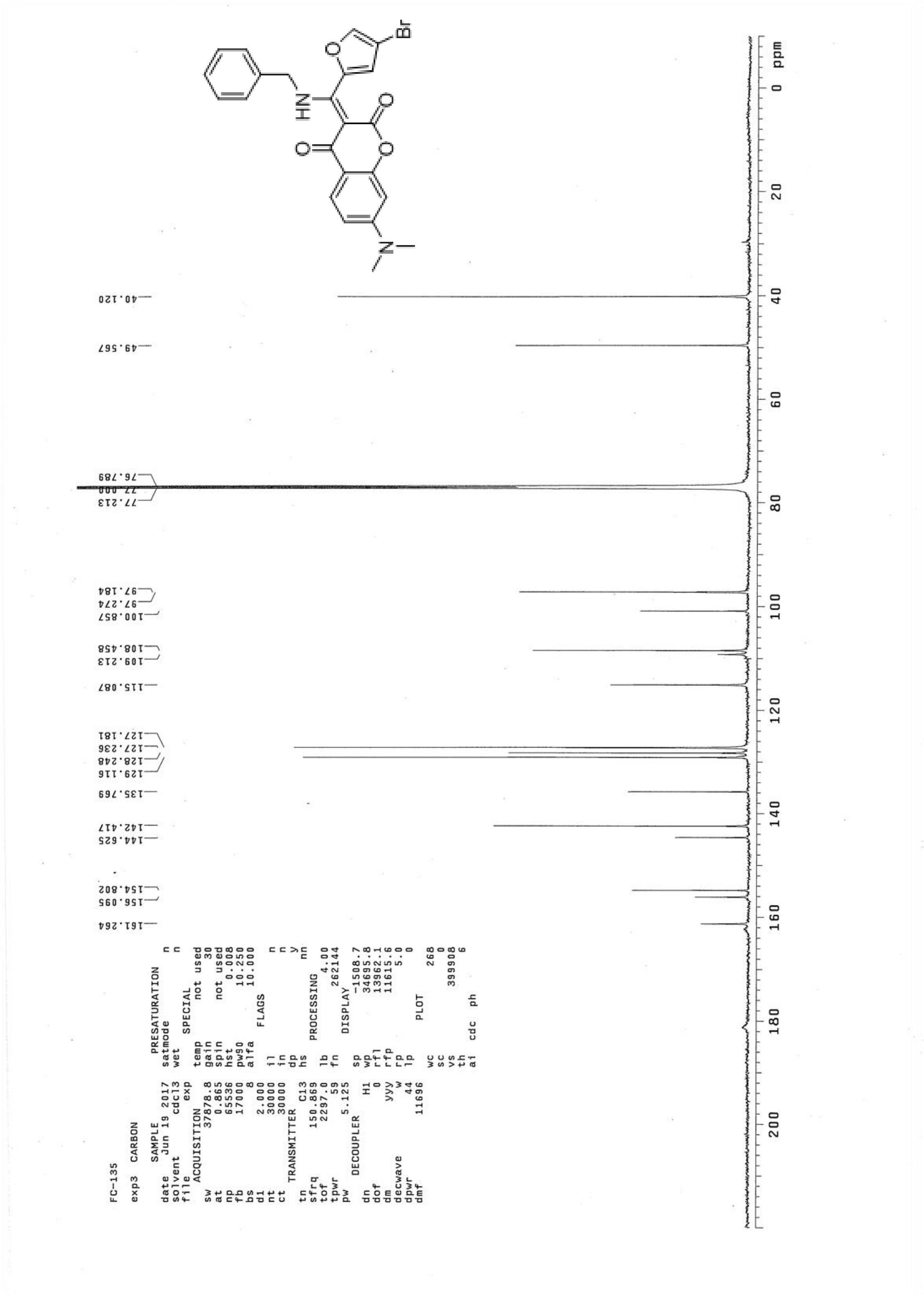

FC-135
exp3 CARBON
SAMPLE
date Jun 19 2017
solvent cdcl3
file exp
ACQUISITION
sw 37878.8
at 0.865
np 65536
fb 17000
bs 8
d1 2.000
nt 30000
ct 30000
TRANSMITTER
tn C13
sfrq 150.869
tof 2297.0
tpwr 59
pw 5.125
DECOUPLER
dn H1
dof 0
dm yyy
decwave W
dpwr 44
dmf 11696
PRESATURATION
satmode n
wet n
SPECIAL
temp not used
gain 30
spin not used
hst 0.008
pw90 10.250
alfa 10.000
FLAGS
il n
in n
dp y
hs nn
PROCESSING
lb 4.00
fn 262144
DISPLAY
sp -1508.7
wp 34695.8
rfl 13962.1
rfp 11615.6
rp 5.0
lp 0
PLOT
wc 268
sc 0
vs 399908
th 6
ai cdc ph
40.120
49.567
76.789
77.000
77.213
97.184
97.274
100.857
108.458
109.213
115.087
127.181
127.236
128.248
129.116
135.769
142.417
144.625
154.802
156.095
161.264
HN
O
Br
N
ppm
0
20
40
60
80
100
120
140
160
180
200

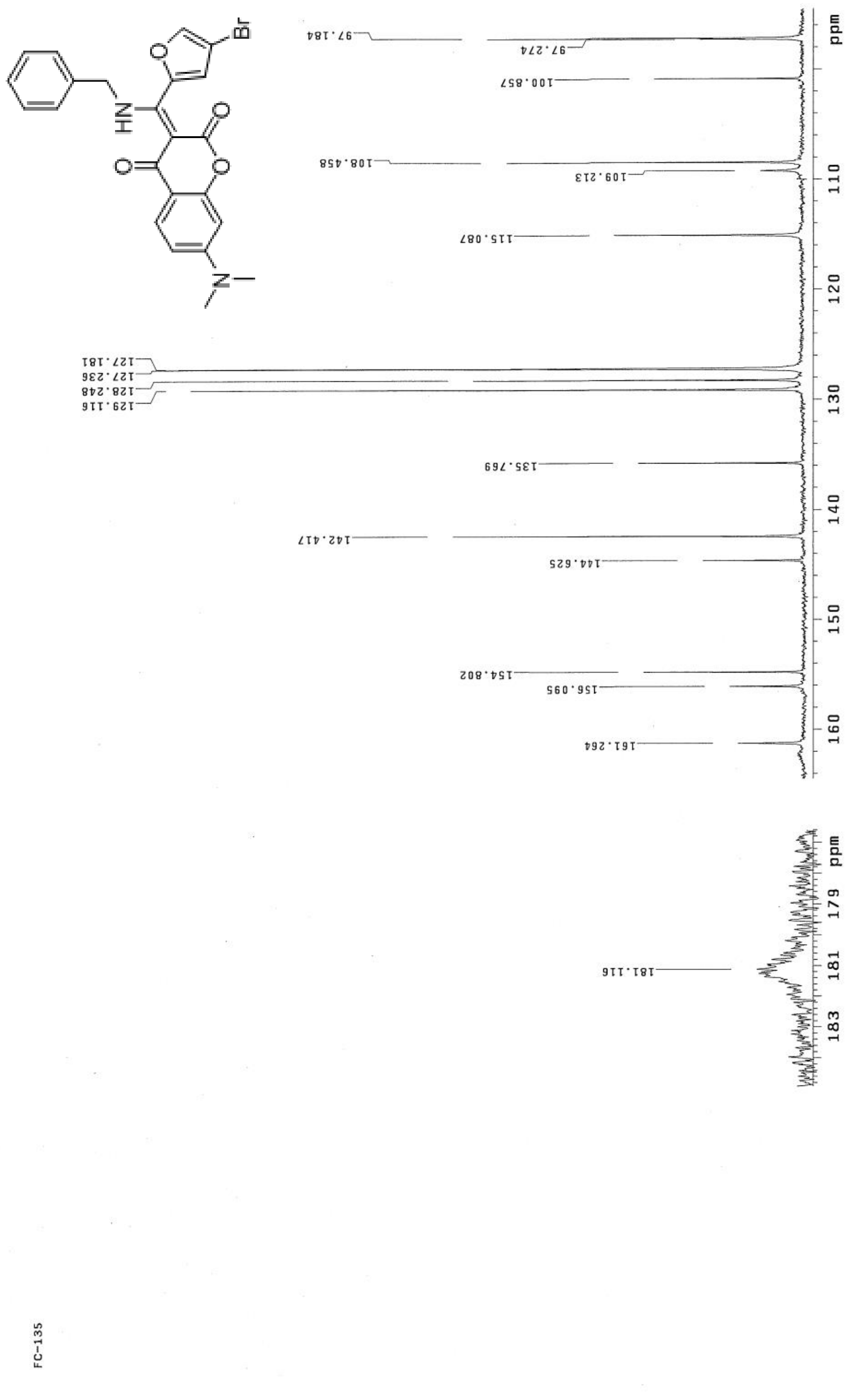
Br
O
HN
N
97.184
97.274
100.857
108.458
109.213
115.087
127.181
127.236
128.248
129.116
135.769
142.417
144.625
154.802
156.095
161.264
181.116
ppm
110
120
130
140
150
160
179
181
183
FC-135

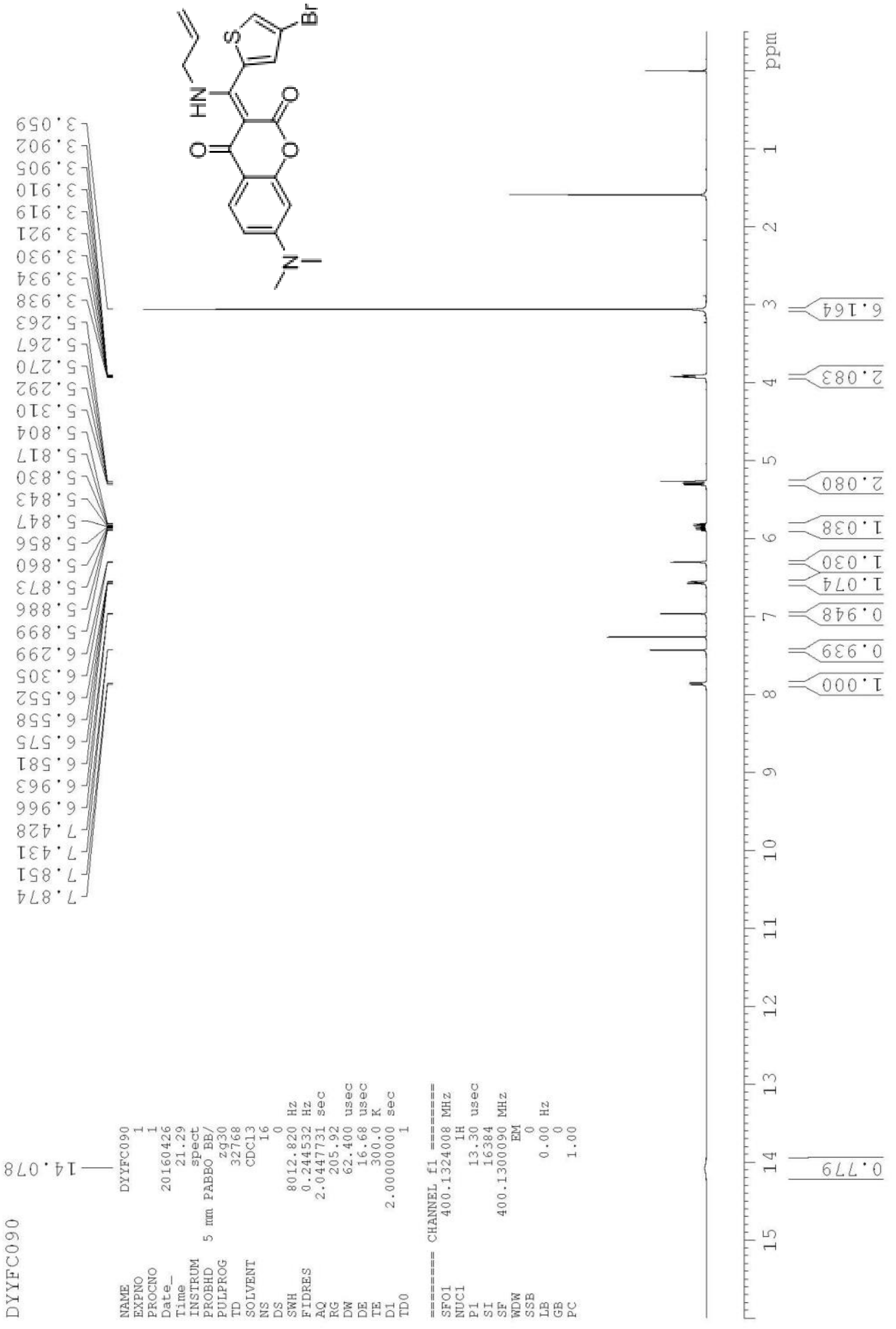

DYYFC090
14.078
3.059
3.902
3.905
3.910
3.919
3.921
3.930
3.934
3.938
5.263
5.267
5.270
5.292
5.310
5.804
5.817
5.830
5.843
5.847
5.856
5.860
5.873
5.886
5.899
6.299
6.305
6.552
6.558
6.575
6.581
6.963
6.966
7.428
7.431
7.851
7.874
NAME DYYFC090
EXPNO 1
PROCNO 1
Date_ 20160426
Time 21.29
INSTRUM spect
PROBHD 5 mm PABBO BB/
PULPROG zg30
TD 32768
SOLVENT CDCl3
NS 16
DS 0
SWH 8012.820 Hz
FIDRES 0.244532 Hz
AQ 2.0447731 sec
RG 205.92
DW 62.400 usec
DE 16.68 usec
TE 300.0 K
D1 2.00000000 sec
TD0 1
======== CHANNEL f1 ========
SFO1 400.1324008 MHz
NUC1 1H
P1 13.30 usec
SI 16384
SF 400.1300090 MHz
WDW EM
SSB 0
LB 0.00 Hz
GB 0
PC 1.00
HN
S
Br
O
N
0.779
1.000
0.939
0.948
1.074
1.030
1.038
2.080
2.083
6.164
ppm

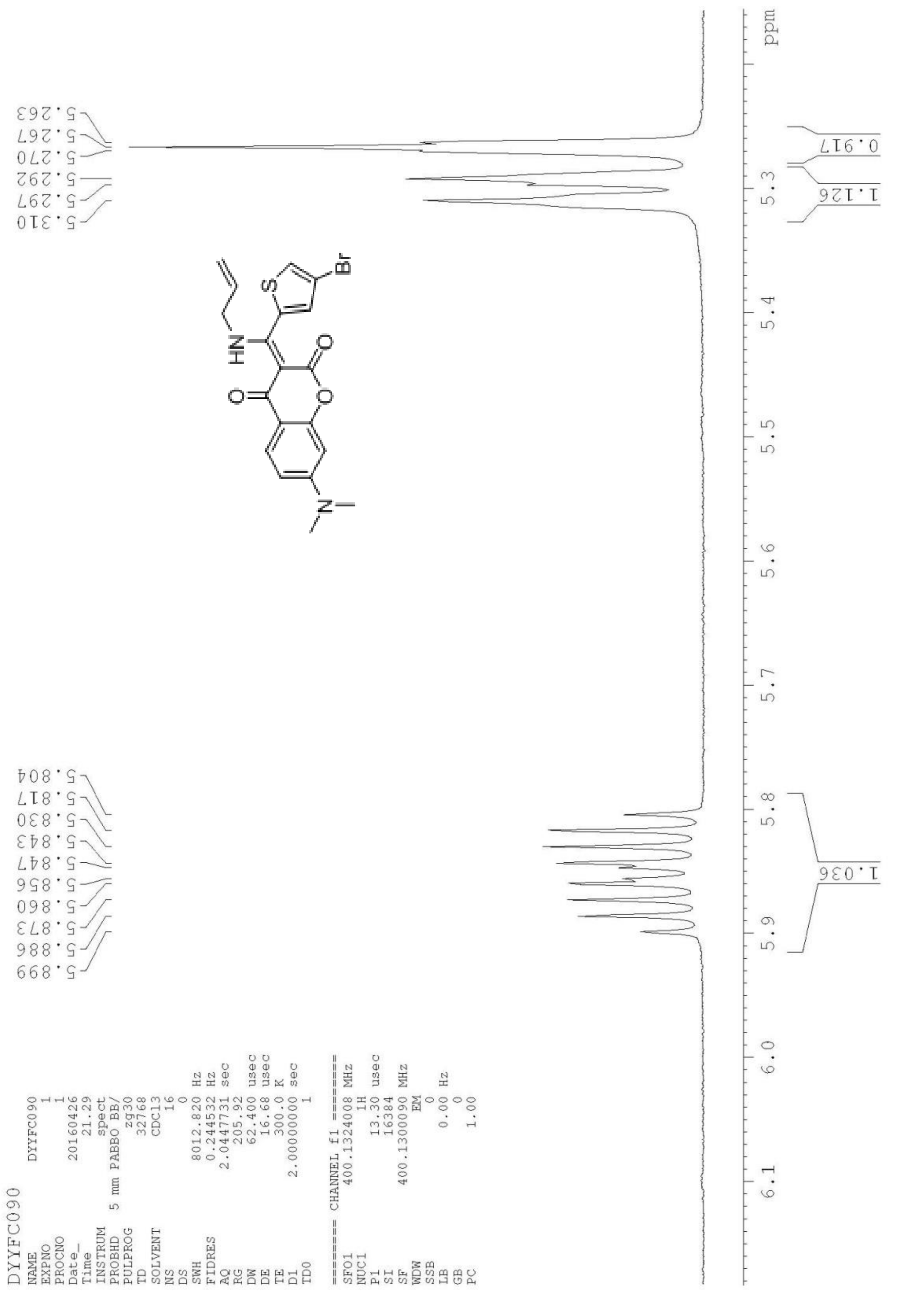
DYYFC090
NAME DYYFC090
EXPNO 1
PROCNO 1
Date_ 20160426
Time 21.29
INSTRUM spect
PROBHD 5 mm PABBO BB/
PULPROG zg30
TD 32768
SOLVENT CDCl3
NS 16
DS 0
SWH 8012.820 Hz
FIDRES 0.244532 Hz
AQ 2.0447731 sec
RG 205.92
DW 62.400 usec
DE 16.68 usec
TE 300.0 K
D1 2.00000000 sec
TD0 1
======== CHANNEL f1 ========
SFO1 400.1324008 MHz
NUC1 1H
P1 13.30 usec
SI 16384
SF 400.1300090 MHz
WDW EM
SSB 0
LB 0.00 Hz
GB 0
PC 1.00
5.263
5.267
5.270
5.292
5.297
5.310
5.804
5.817
5.830
5.843
5.847
5.856
5.860
5.873
5.886
5.899
HN
S
Br
O
N
0.917
1.126
1.036
5.3
5.4
5.5
5.6
5.7
5.8
5.9
6.0
6.1
ppm

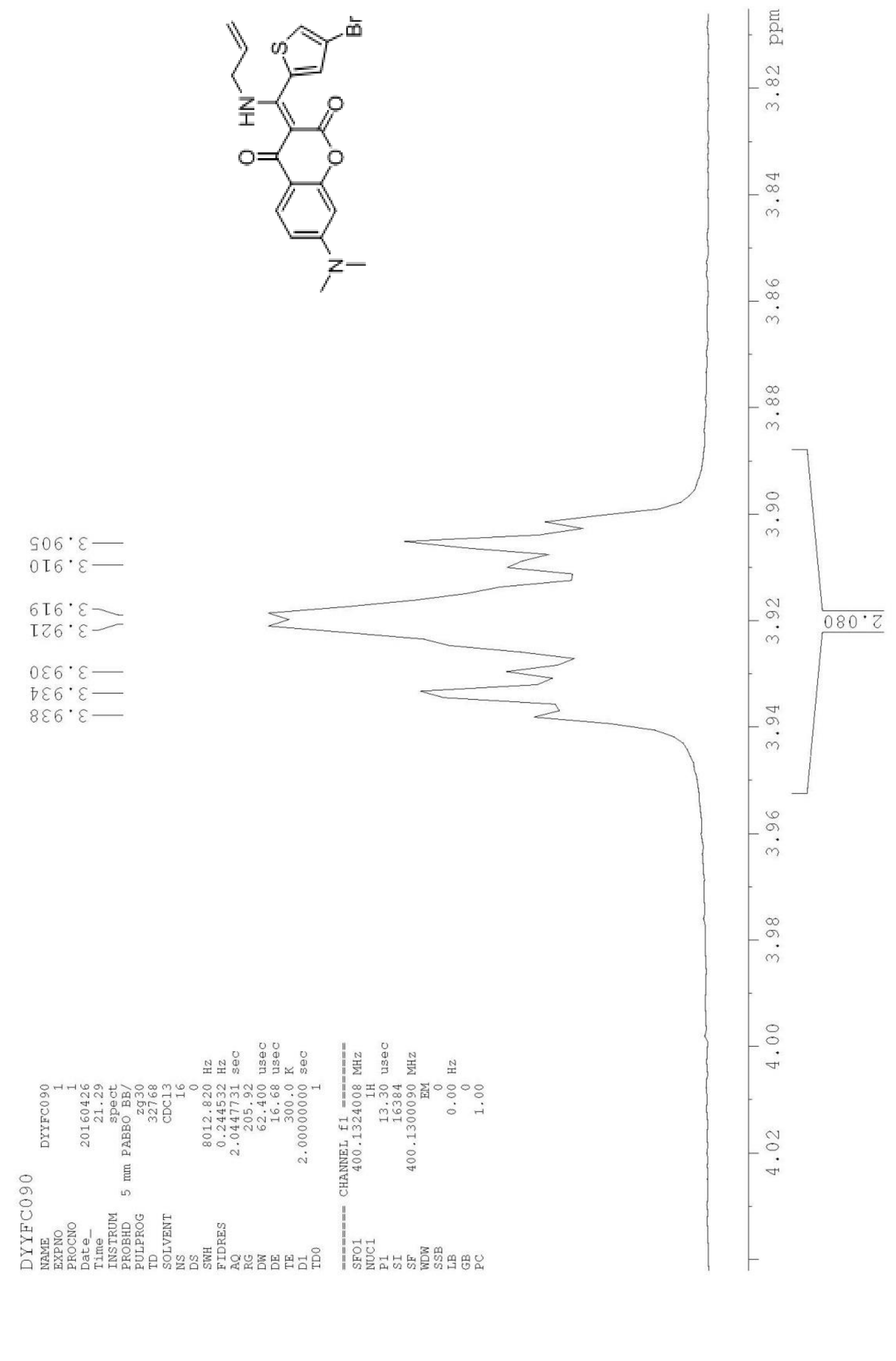
DYYFC090
NAME DYYFC090
EXPNO 1
PROCNO 1
Date_ 20160426
Time 21.29
INSTRUM spect
PROBHD 5 mm PABBO BB/
PULPROG zg30
TD 32768
SOLVENT CDCl3
NS 16
DS 0
SWH 8012.820 Hz
FIDRES 0.244532 Hz
AQ 2.0447731 sec
RG 205.92
DW 62.400 usec
DE 16.68 usec
TE 300.0 K
D1 2.00000000 sec
TD0 1
======== CHANNEL f1 ========
SFO1 400.1324008 MHz
NUC1 1H
P1 13.30 usec
SI 16384
SF 400.1300090 MHz
WDW EM
SSB 0
LB 0.00 Hz
GB 0
PC 1.00
3.905
3.910
3.919
3.921
3.930
3.934
3.938
HN
S
Br
O
O
O
N
2.080
3.82
3.84
3.86
3.88
3.90
3.92
3.94
3.96
3.98
4.00
4.02
ppm

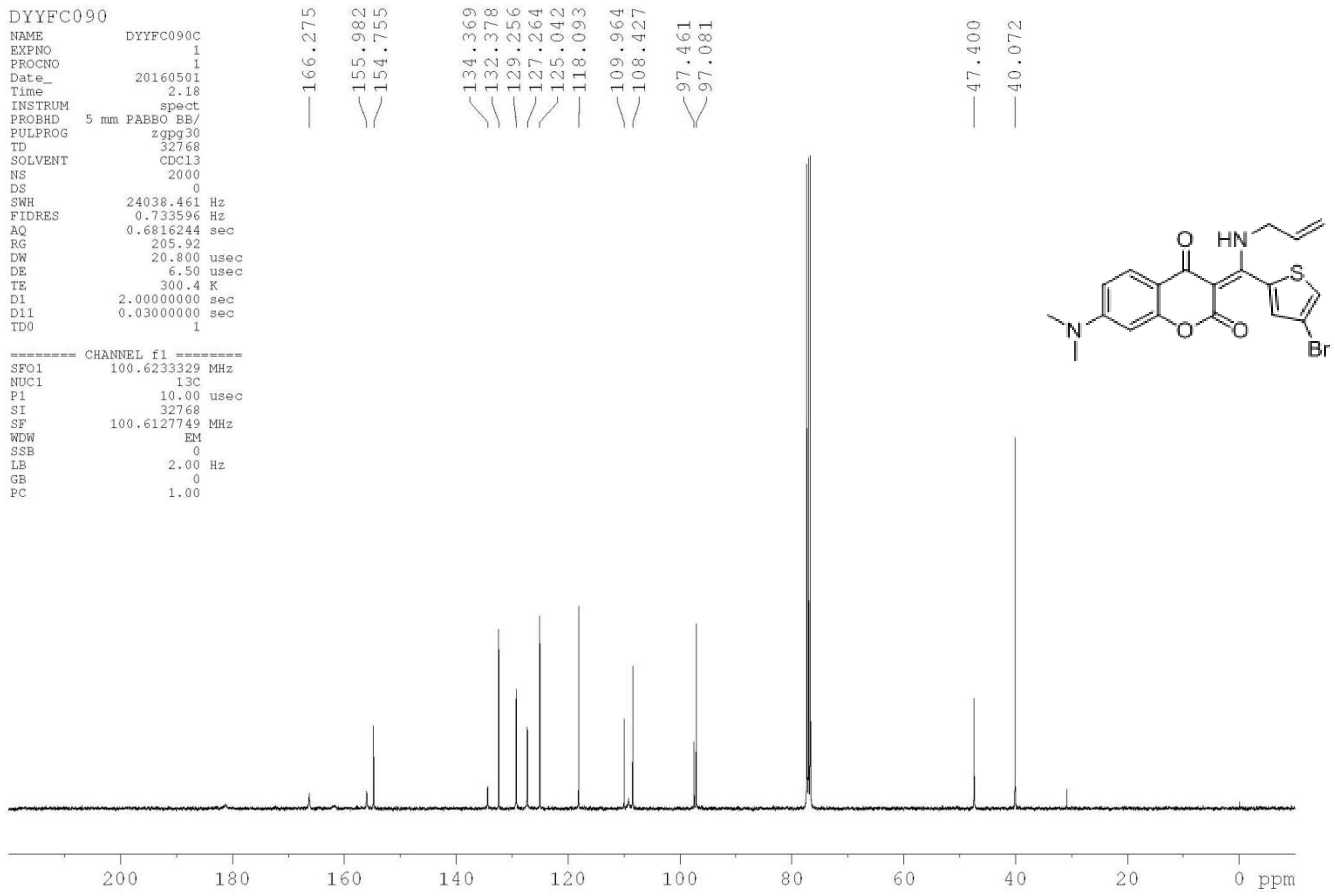
DYYFC090
NAME DYYFC090C
EXPNO 1
PROCNO 1
Date_ 20160501
Time 2.18
INSTRUM spect
PROBHD 5 mm PABBO BB/
PULPROG zgpg30
TD 32768
SOLVENT CDCl3
NS 2000
DS 0
SWH 24038.461 Hz
FIDRES 0.733596 Hz
AQ 0.6816244 sec
RG 205.92
DW 20.800 usec
DE 6.50 usec
TE 300.4 K
D1 2.00000000 sec
D11 0.03000000 sec
TD0 1
======== CHANNEL f1 ========
SFO1 100.6233329 MHz
NUC1 13C
P1 10.00 usec
SI 32768
SF 100.6127749 MHz
WDW EM
SSB 0
LB 2.00 Hz
GB 0
PC 1.00
166.275
155.982
154.755
134.369
132.378
129.256
127.264
125.042
118.093
109.964
108.427
97.461
97.081
47.400
40.072
O
HN
S
N
O
O
Br
200
180
160
140
120
100
80
60
40
20
0 ppm

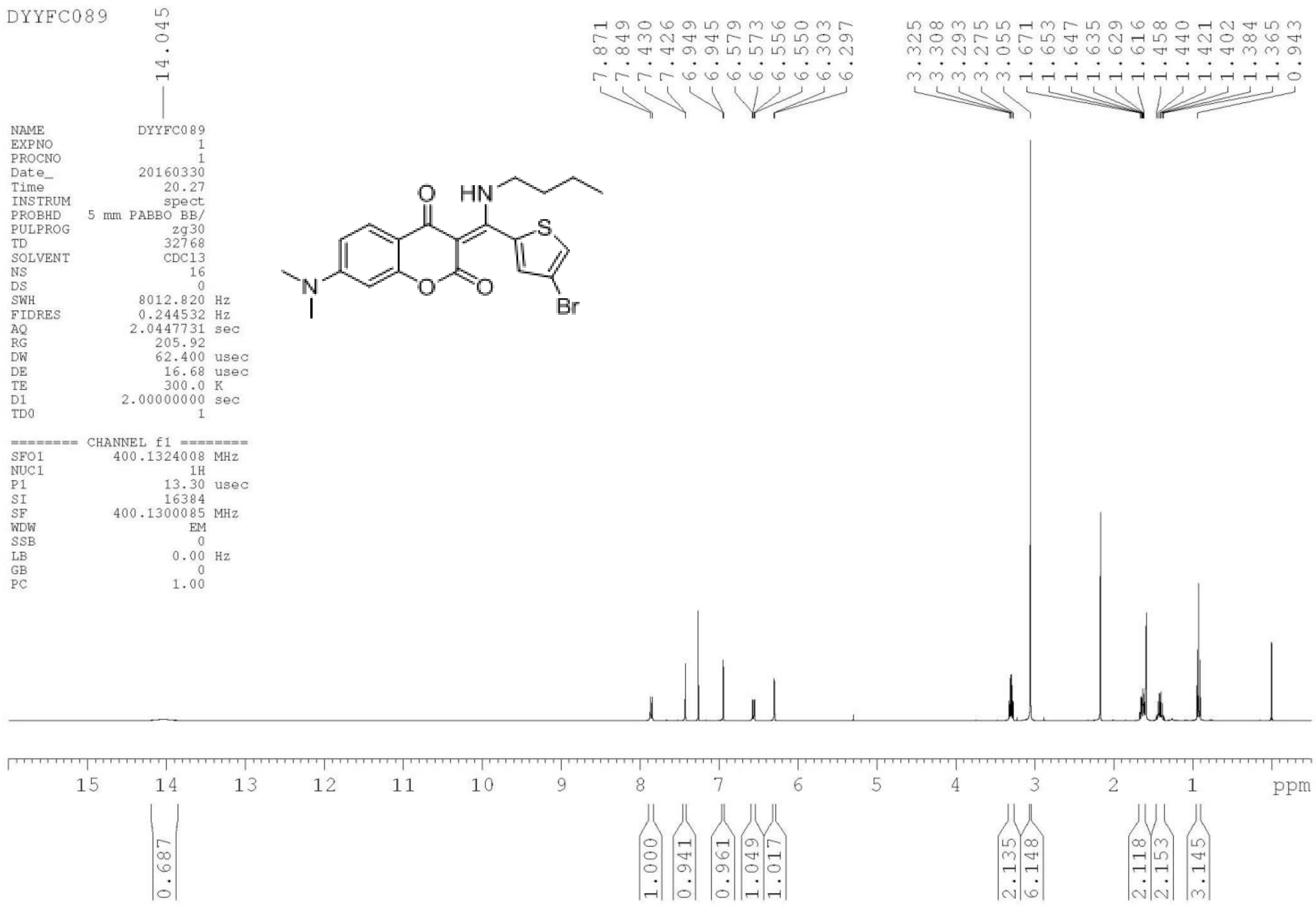
DYYFC089
14.045
7.871
7.849
7.430
7.426
6.949
6.945
6.579
6.573
6.556
6.550
6.303
6.297
3.325
3.308
3.293
3.275
3.055
1.671
1.653
1.647
1.635
1.629
1.616
1.458
1.440
1.421
1.402
1.384
1.365
0.943
NAME DYYFC089
EXPNO 1
PROCNO 1
Date_ 20160330
Time 20.27
INSTRUM spect
PROBHD 5 mm PABBO BB/
PULPROG zg30
TD 32768
SOLVENT CDCl3
NS 16
DS 0
SWH 8012.820 Hz
FIDRES 0.244532 Hz
AQ 2.0447731 sec
RG 205.92
DW 62.400 usec
DE 16.68 usec
TE 300.0 K
D1 2.00000000 sec
TD0 1
======== CHANNEL f1 ========
SFO1 400.1324008 MHz
NUC1 1H
P1 13.30 usec
SI 16384
SF 400.1300085 MHz
WDW EM
SSB 0
LB 0.00 Hz
GB 0
PC 1.00
O
HN
S
N
O
O
Br
15
14
13
12
11
10
9
8
7
6
5
4
3
2
1
ppm
0.687
1.000
0.941
0.961
1.049
1.017
2.135
6.148
2.118
2.153
3.145

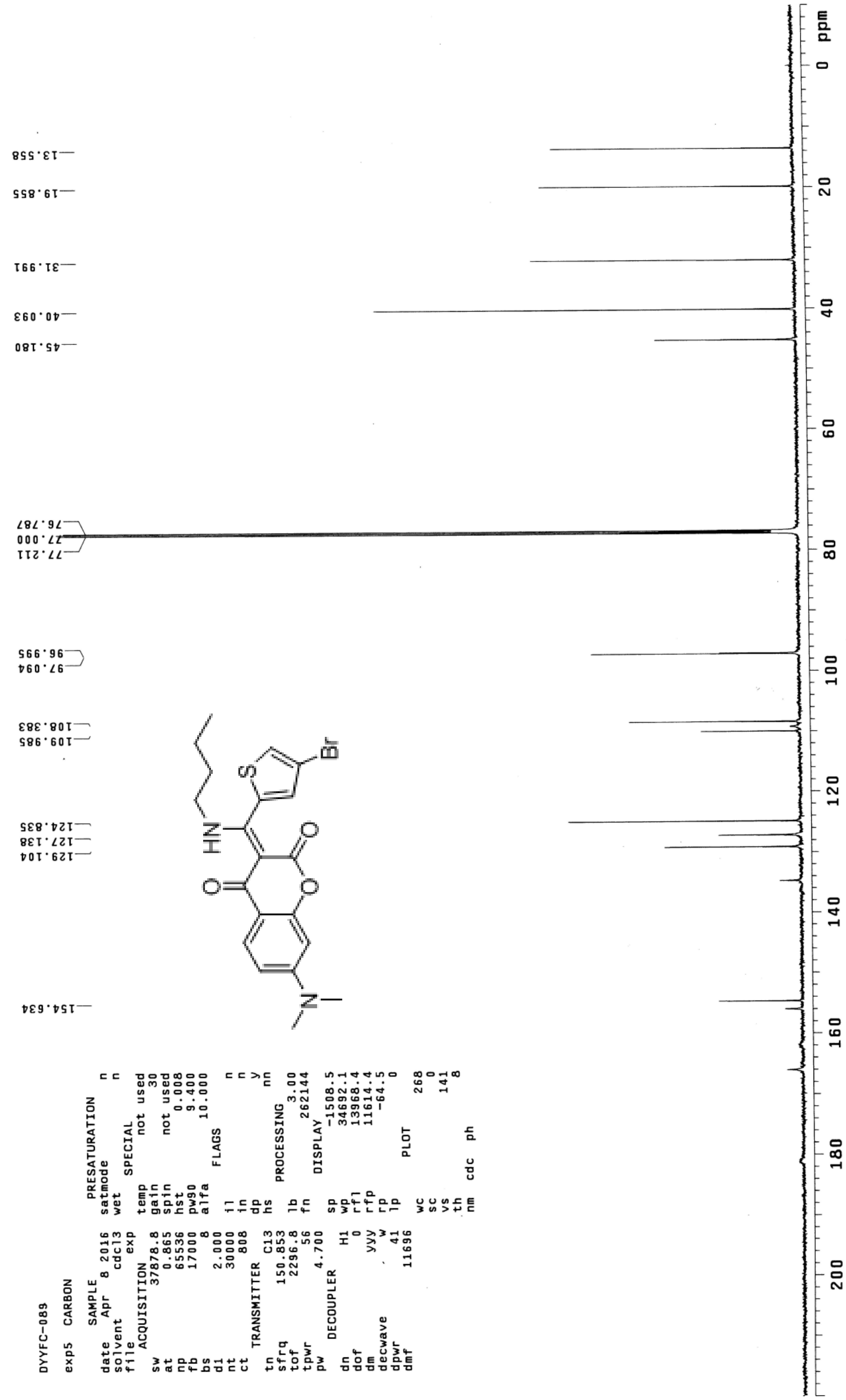
DYYFC-089
exp5 CARBON
SAMPLE
date Apr 8 2016
solvent cdcl3
file exp
ACQUISITION
sw 37878.8
at 0.865
np 65536
fb 17000
bs 8
d1 2.000
nt 30000
ct 808
TRANSMITTER
tn C13
sfrq 150.853
tof 2296.8
tpwr 56
pw 4.700
DECOUPLER
dn H1
dof 0
dm yyy
decwave w
dpwr 41
dmf 11696
PRESATURATION
satmode n
wet n
SPECIAL
temp not used
gain 30
spin not used
hst 0.008
pw90 9.400
alfa 10.000
FLAGS
il n
in n
dp y
hs nn
PROCESSING
lb 3.00
fn 262144
DISPLAY
sp -1508.5
wp 34692.1
rfl 13968.4
rfp 11614.4
rp -64.5
lp 0
PLOT
wc 268
sc 0
vs 141
th 8
nm cdc ph
154.634
129.104
127.138
124.835
109.985
108.383
97.094
96.995
77.211
77.000
76.787
45.180
40.093
31.991
19.855
13.558
HN
S
Br
O
N
200
180
160
140
120
100
80
60
40
20
0 ppm

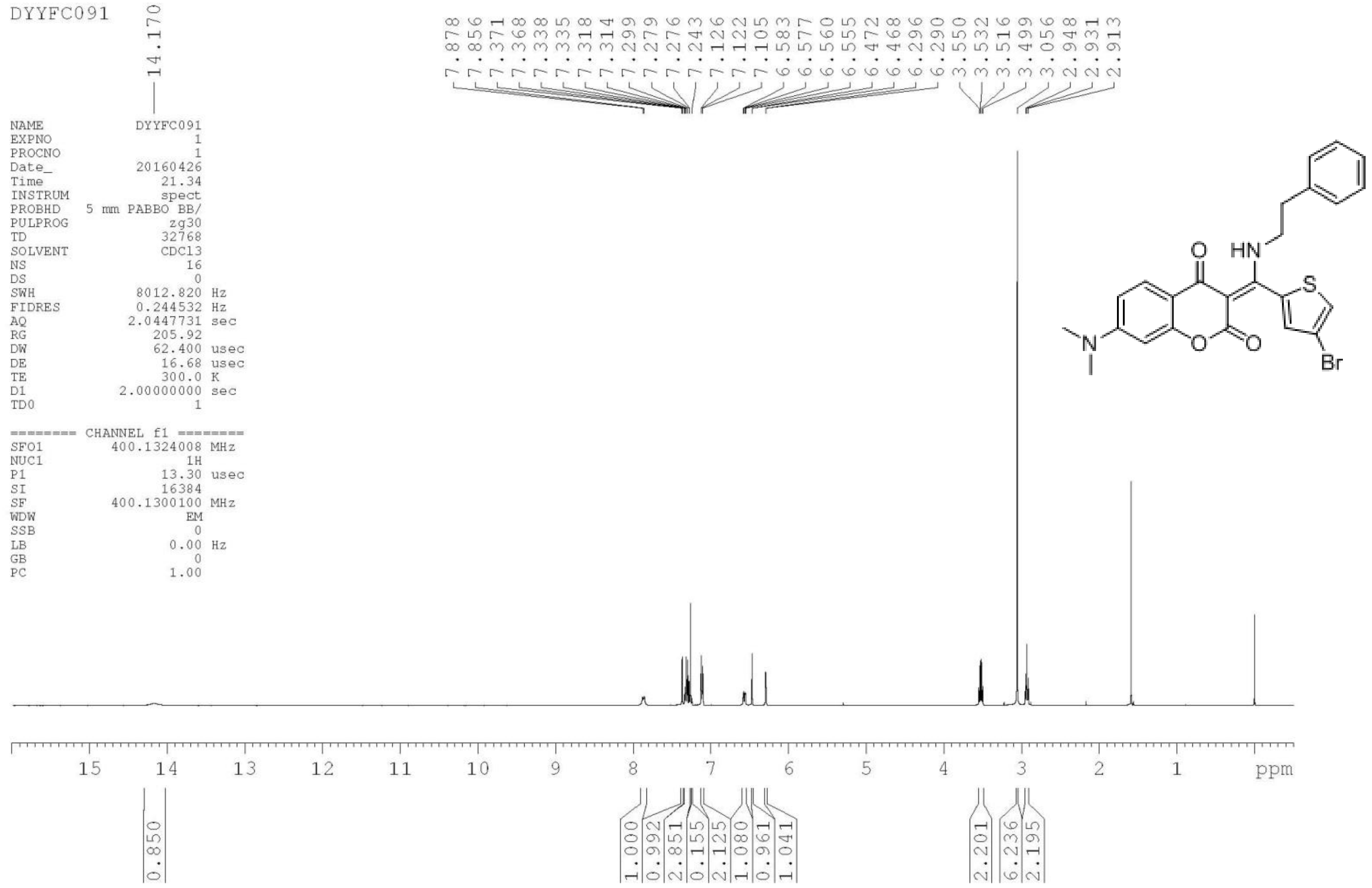
DYYFC091
14.170
7.878 7.856 7.371 7.368 7.338 7.335 7.318 7.314 7.299 7.279 7.276 7.243 7.126 7.122 7.105 6.583 6.577 6.560 6.555 6.472 6.468 6.296 6.290 3.550 3.532 3.516 3.499 3.056 2.948 2.931 2.913
NAME DYYFC091
EXPNO 1
PROCNO 1
Date_ 20160426
Time 21.34
INSTRUM spect
PROBHD 5 mm PABBO BB/
PULPROG zg30
TD 32768
SOLVENT CDCl3
NS 16
DS 0
SWH 8012.820 Hz
FIDRES 0.244532 Hz
AQ 2.0447731 sec
RG 205.92
DW 62.400 usec
DE 16.68 usec
TE 300.0 K
D1 2.00000000 sec
TD0 1
======== CHANNEL f1 ========
SFO1 400.1324008 MHz
NUC1 1H
P1 13.30 usec
SI 16384
SF 400.1300100 MHz
WDW EM
SSB 0
LB 0.00 Hz
GB 0
PC 1.00
O
HN
S
N
O
O
Br
15 14 13 12 11 10 9 8 7 6 5 4 3 2 1 ppm
0.850
1.000 0.992 2.851 0.155 2.125 1.080 0.961 1.041
2.201 6.236 2.195

DYYFC091C

NAME	DYYFC091C
EXPNO	1
PROCNO	1
Date_	20160501
Time	3.54
INSTRUM	spect
PROBHD	5 mm PABBO BB/
PULPROG	zgpg30
TD	32768
SOLVENT	CDCl3
NS	2000
DS	0
SWH	24038.461 Hz
FIDRES	0.733596 Hz
AQ	0.6816244 sec
RG	205.92
DW	20.800 usec
DE	6.50 usec
TE	300.4 K
D1	2.00000000 sec
D11	0.03000000 sec
TD0	1

======== CHANNEL f1 ========

SFO1	100.6233329 MHz
NUC1	13C
P1	10.00 usec
SI	32768
SF	100.6127757 MHz
WDW	EM
SSB	0
LB	2.00 Hz
GB	0
PC	1.00

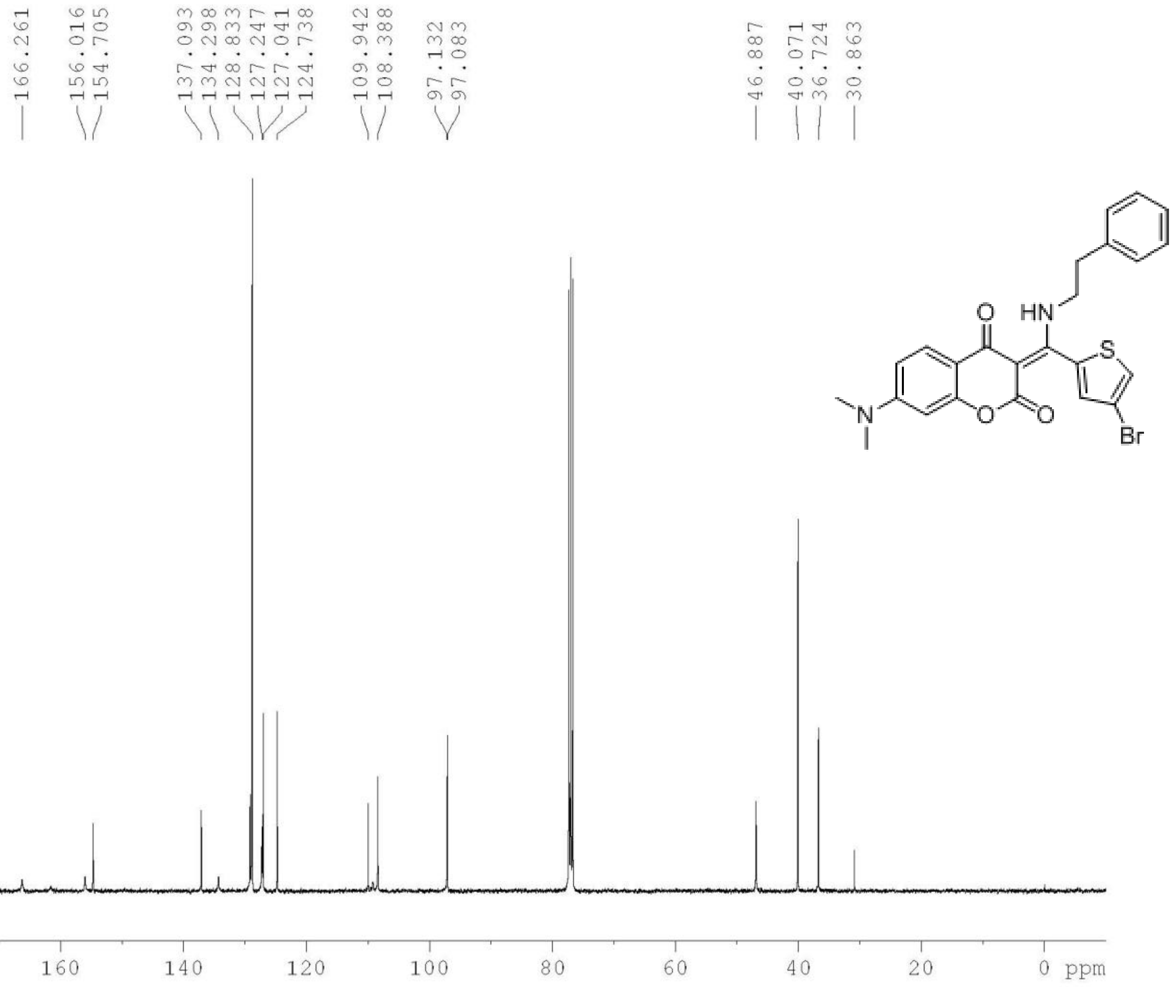

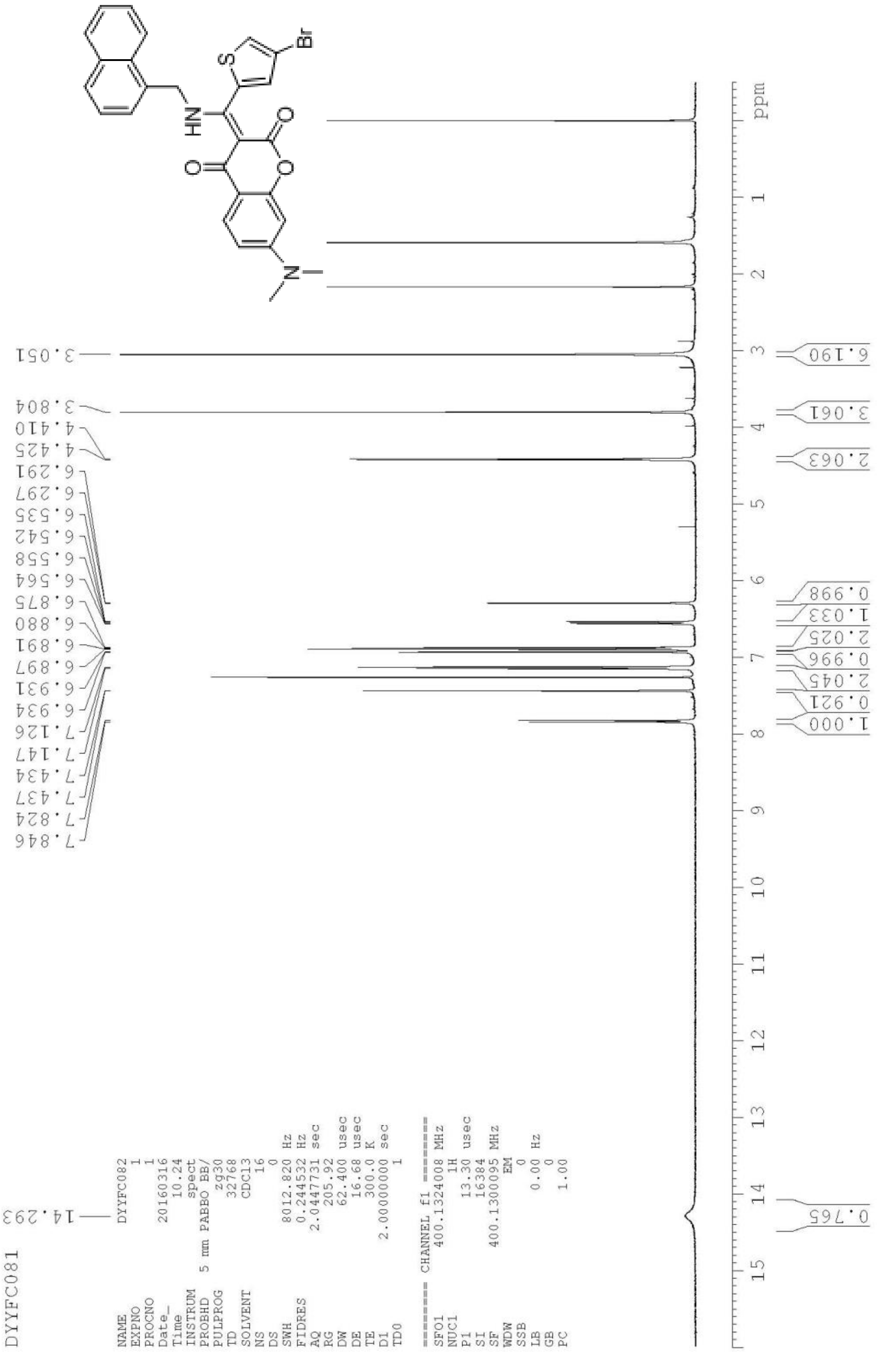

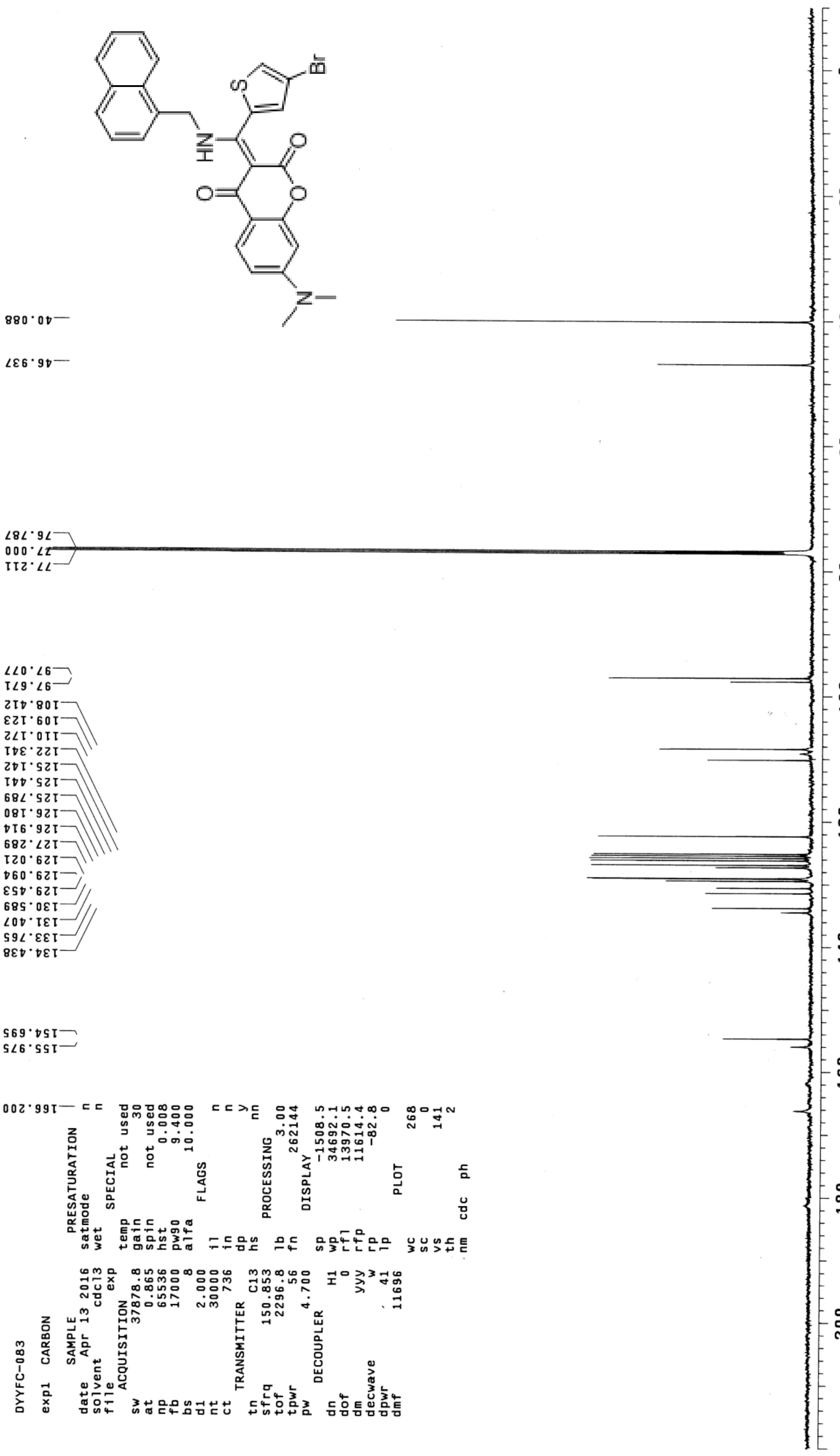
DYYFC-083
exp1 CARBON
SAMPLE
date Apr 13 2016
solvent cdcl3
file exp
ACQUISITION
sw 37878.8
at 0.865
np 65536
fb 17000
bs 8
d1 2.000
nt 30000
ct 736
TRANSMITTER
tn C13
sfrq 150.853
tof 2296.8
tpwr 56
pw 4.700
DECOUPLER
dn H1
dof 0
dm yyy
decwave w
dpwr 41
dmf 11696
PRESATURATION
satmode nn
wet n
SPECIAL
temp not used
gain 30
spin not used
hst 0.008
pw90 9.400
alfa 10.000
FLAGS
il n
in n
dp y
hs nn
PROCESSING
lb 3.00
fn 262144
DISPLAY
sp -1508.5
wp 34692.1
rfl 13970.5
rfp 11614.4
rp -82.8
lp 0
PLOT
wc 268
sc 0
vs 141
th 2
nm cdc ph
166.200
155.975
154.695
134.438
133.765
131.407
130.589
129.453
129.094
129.021
127.289
126.914
126.180
125.789
125.441
125.142
122.341
110.172
109.123
108.412
97.671
97.077
77.211
77.000
76.787
46.937
40.088
HN
O
O
O
S
Br
N
200
180
160
140
120
100
80
60
40
20
0
ppm

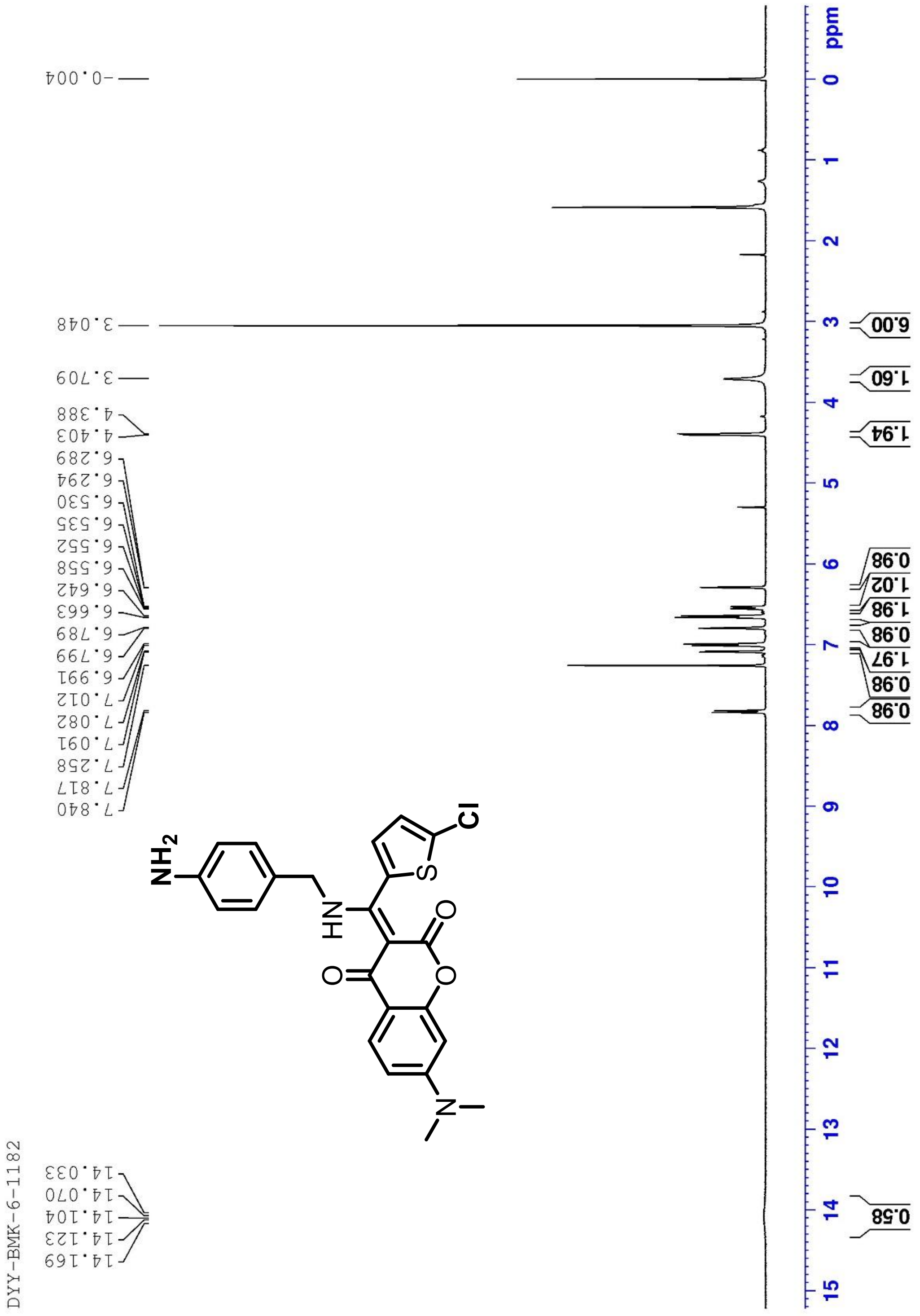
DYY-BMK-6-1182
-0.004
3.048
3.709
4.388
4.403
6.289
6.294
6.530
6.535
6.552
6.558
6.642
6.663
6.789
6.799
6.991
7.012
7.082
7.091
7.258
7.817
7.840
14.033
14.070
14.104
14.123
14.169
6.00
1.60
1.94
0.98
1.02
1.98
0.98
1.97
0.98
0.98
0.58
ppm
NH2
HN
S
Cl
O
N

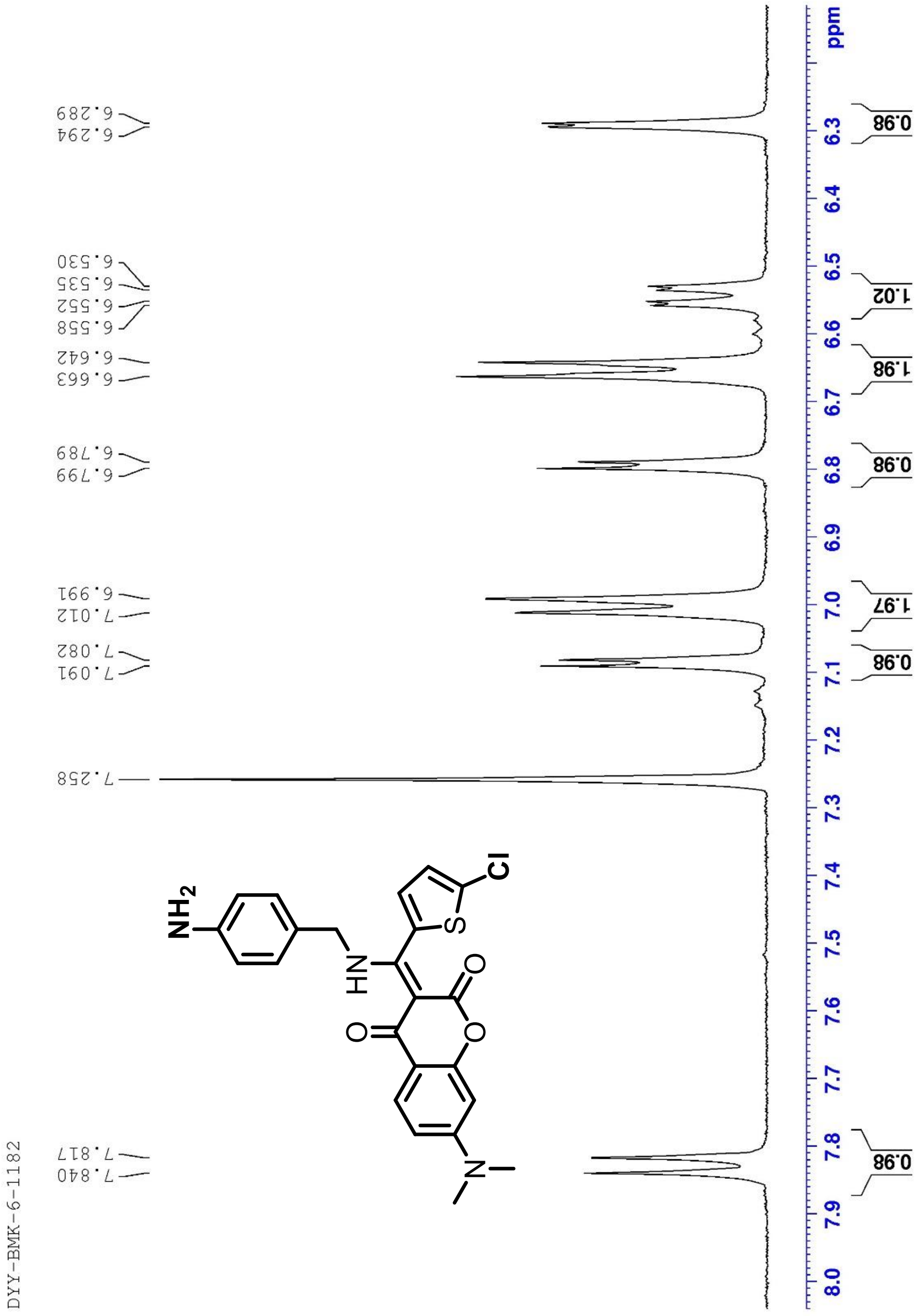
DYY-BMK-6-1182
6.289
6.294
6.530
6.535
6.552
6.558
6.642
6.663
6.789
6.799
6.991
7.012
7.082
7.091
7.258
7.817
7.840
0.98
1.02
1.98
0.98
1.97
0.98
0.98
ppm
6.3
6.4
6.5
6.6
6.7
6.8
6.9
7.0
7.1
7.2
7.3
7.4
7.5
7.6
7.7
7.8
7.9
8.0
NH2
HN
S
Cl
O
O
O
N

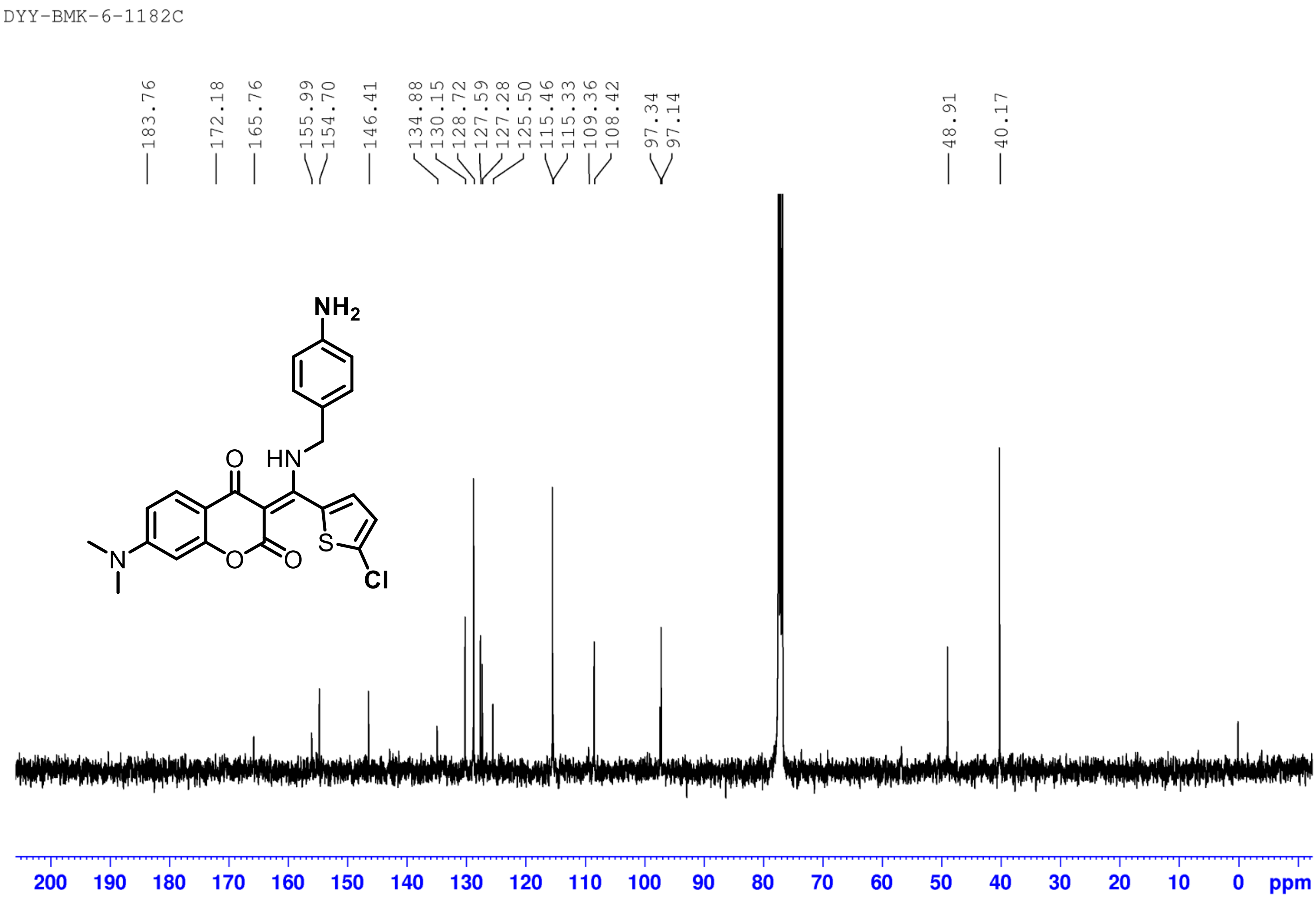
DYY-BMK-6-1182C
183.76
172.18
165.76
155.99
154.70
146.41
134.88
130.15
128.72
127.59
127.28
125.50
115.46
115.33
109.36
108.42
97.34
97.14
48.91
40.17
NH2
HN
O
N
S
Cl
200 190 180 170 160 150 140 130 120 110 100 90 80 70 60 50 40 30 20 10 0 ppm

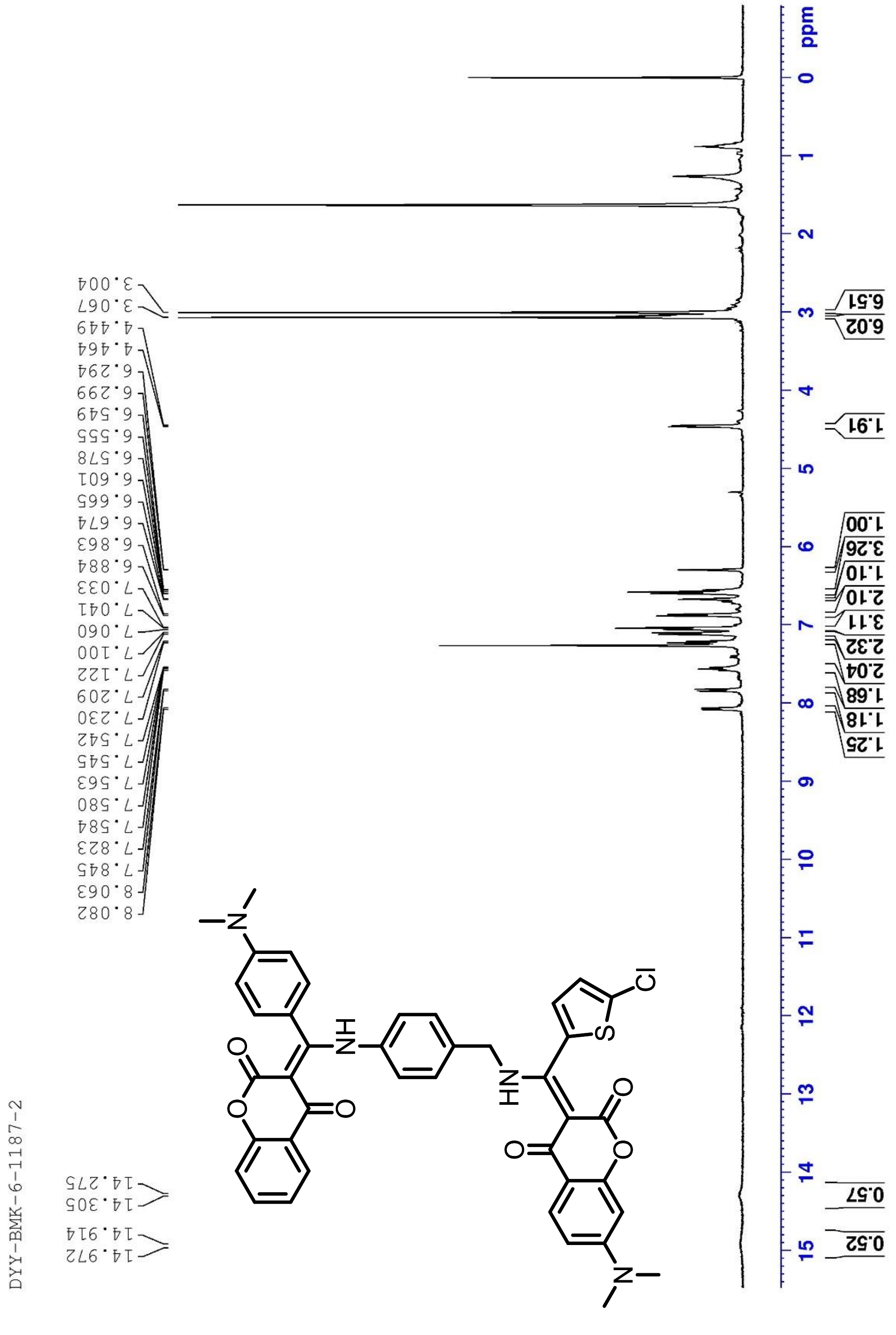
DYY-BMK-6-1187-2
14.972
14.914
14.305
14.275
8.082
8.063
7.845
7.823
7.584
7.580
7.563
7.545
7.542
7.230
7.209
7.122
7.100
7.060
7.041
7.033
6.884
6.863
6.674
6.665
6.601
6.578
6.555
6.549
6.299
6.294
4.464
4.449
3.067
3.004
0.52
0.57
1.25
1.18
1.68
2.04
2.32
3.11
2.10
1.10
3.26
1.00
1.91
6.02
6.51
15
14
13
12
11
10
9
8
7
6
5
4
3
2
1
0
ppm
NH
HN
O
S
Cl
N

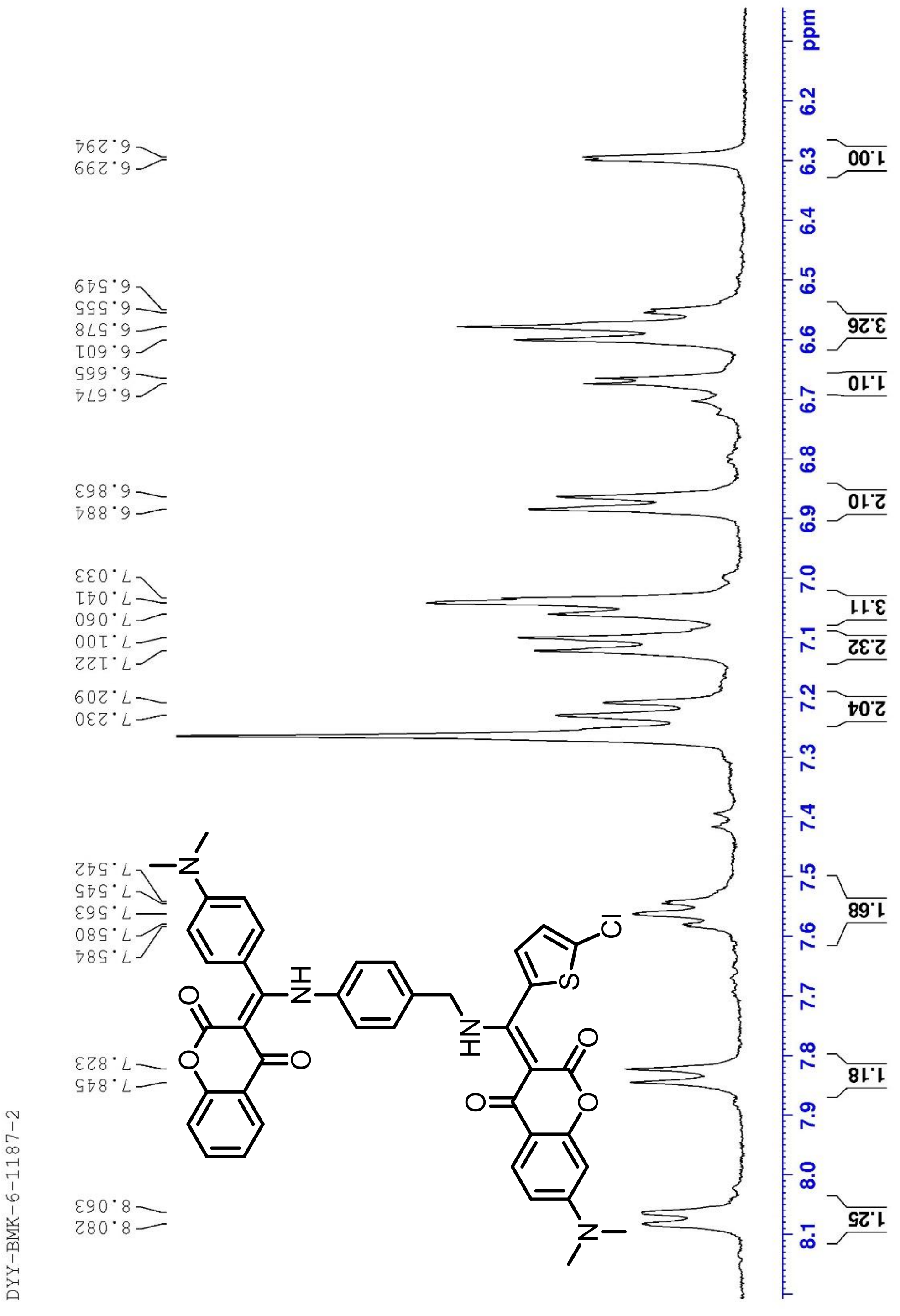
DYY-BMK-6-1187-2
ppm
1.00
3.26
1.10
2.10
3.11
2.32
2.04
1.68
1.18
1.25

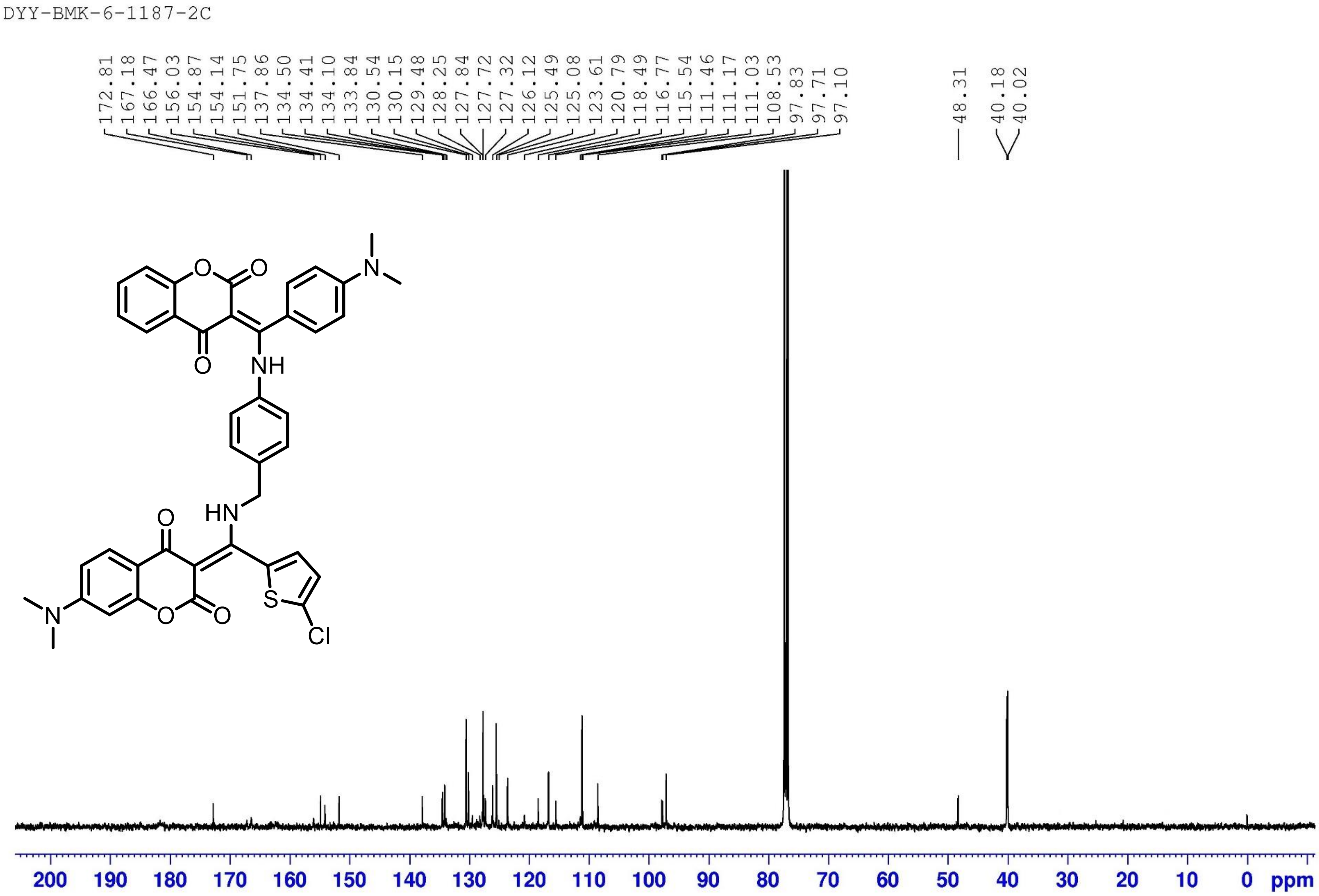
DYY-BMK-6-1187-2C
172.81
167.18
166.47
156.03
154.87
154.14
151.75
137.86
134.50
134.41
134.10
133.84
130.54
130.15
129.48
128.25
127.84
127.72
127.32
126.12
125.49
125.08
123.61
120.79
118.49
116.77
115.54
111.46
111.17
111.03
108.53
97.83
97.71
97.10
48.31
40.18
40.02
200 190 180 170 160 150 140 130 120 110 100 90 80 70 60 50 40 30 20 10 0 ppm
